# DAZAP1 maintains gastric cancer stemness by inducing mitophagy

**DOI:** 10.1172/jci.insight.175422

**Published:** 2025-05-22

**Authors:** Peiling Zhang, Wei Wang, Hong Xiang, Yun Zhou, Qian Peng, Guolong Liu, Zhi-Xiang Xu, Lin Lu

**Affiliations:** 1 Department of Medical Oncology, Guangzhou First People’s Hospital, South China University of Technology, Guangzhou, China.; 2 Department of Medical Oncology, Guangzhou First People’s Hospital, Guangzhou Medical University, Guangzhou, China.; 3 Department of Gastric Surgery, Sun Yat-sen University Cancer Center, Guangzhou, Guangdong, China.; 4 School of Life Sciences, Henan University, Kaifeng, Henan Province, China.

**Keywords:** Cell biology, Oncology, Stem cells, Autophagy, Gastric cancer, Mitochondria

## Abstract

Stem cells play a pivotal role in the malignant behavior of gastric cancer (GC), complicating its treatment and prognosis. However, the regulatory mechanisms of GC stem cells (GCSCs) remain poorly understood. DAZ-associated protein 1 (DAZAP1), a splicing regulator linked to various malignancies, has an unclear role in GC. This study investigated DAZAP1’s impact on GC stemness and its mechanisms. DAZAP1 promoted tumor progression in GCSCs, as shown by sphere formation assays and stemness marker analysis. Functional enrichment analysis suggested that DAZAP1 enhanced tumor stemness by promoting oxidative phosphorylation (OXPHOS), which was validated through Seahorse assays and measurements of mitochondrial potential. Transmission electron microscopy and immunofluorescence analyses demonstrated that DAZAP1 promoted mitophagy. RNA immunoprecipitation and PCR analysis revealed that DAZAP1 regulated the splicing and expression of the mitophagy-related gene *ULK1* through nonsense-mediated mRNA decay. Rescue experiments showed that overexpression of ULK1 reversed the suppression of GC stemness and OXPHOS levels induced by DAZAP1 silencing. Our findings indicate that DAZAP1 reduces *ULK1* decay, thereby activating mitophagy and enhancing OXPHOS to fulfill the metabolic demands of cancer stem cells. These findings highlight the therapeutic potential of DAZAP1 as a target for treating GC.

## Abstract 2

DAZAP1 upregulation in gastric cancer promotes cell stemness through ULK1-mediated mitophagy and oxidative phosphorylation, revealing a key molecular mechanism in GC progression.

## Introduction

Gastric cancer (GC) is the fifth most common cancer and the fifth leading cause of cancer-related mortality globally, marked by substantial heterogeneity (1, 2). The lack of effective targeted therapies, coupled with the limited response of GC to standard chemotherapy and immunotherapy, leads to a poor prognosis (3, 4). Cancer stem cells (CSCs) represent a distinct subset of cells capable of self-renewal and possessing multipotency, which drive tumor recurrence, metastasis, and resistance to therapy (5). Compared with regular cancer cells, CSCs demonstrate enhanced survival capacity and adaptability within the tumor microenvironment, rendering them highly resistant to conventional treatments such as chemotherapy and radiotherapy (6). This resistance makes CSCs a critical target in current cancer research and therapeutic strategies. Research has demonstrated that CSCs differ from conventional cancer cells in terms of surface markers and signaling pathways. For instance, markers like *POU5F1*, *NANOG*, *SOX2*, *PROM1*, and *ALDH1A1* are commonly utilized to identify CSCs in GC (7, 8). Moreover, signaling pathways such as Notch, Wnt, and Hedgehog are essential for the maintenance of CSC characteristics (9). However, the mechanisms that regulate stemness in GC are still not well understood. Hypothetically, targeting and eliminating this cell subpopulation could potentially lead to complete tumor eradication (10, 11). Nonetheless, research on GC stem cells (GCSCs) remains limited, leading to an absence of well-defined biomarkers for their identification. Furthermore, the pathological factors and genes involved in promoting or sustaining the stemness of GC cells require further investigation.

CSCs play a critical role in tumor initiation and progression, with unique metabolic characteristics enabling them to survive and proliferate in the harsh tumor microenvironment. In contrast with conventional cancer cells that predominantly depend on glycolysis, CSCs have the flexibility to switch between glycolysis and oxidative phosphorylation (OXPHOS) (12–15). This metabolic plasticity enables CSCs to choose the most advantageous energy pathways in response to microenvironmental changes, thereby preserving their stemness and survival capacity. Mitochondria play a central role in CSC metabolism, with their functional state and dynamic changes — such as mitophagy and mitochondrial fusion and fission — directly influencing the metabolic reprogramming and energy production of CSCs (16, 17). Notably, CSCs display heterogeneous metabolic profiles that are heavily reliant on mitochondrial function (18, 19). Mitochondrial balance is maintained through a dynamic interplay between mitochondrial biogenesis and mitophagy. Mitophagy removes damaged or excessive mitochondrial fragments, preserving mitochondrial homeostasis via the autophagy/lysosome pathway (20). Extensive research indicates that increased mitophagy is essential for maintaining the metabolic plasticity and stemness of cancer cells (21, 22). For instance, Feng et al. demonstrated that mitophagy upregulates the expression of stemness biomarkers and promotes the differentiation of bone marrow mesenchymal stem cells (23). Inhibition of autophagy has been shown to suppress CSC activity in pancreatic cancer (24). In summary, existing evidence underscores the importance of mitophagy in regulating GCSCs. Given that mitochondria play a central role in CSC metabolism, their dynamic regulation through processes such as OXPHOS and mitophagy is crucial for sustaining the metabolic flexibility and survival of CSCs. However, the specific mechanisms through which GC cells regulate mitophagy to sustain stemness remain insufficiently understood. Currently, research on mitophagy in GC remains inconclusive. Xiao et al. demonstrated that AMP-activated protein kinase (AMPK) promotes PINK1/Parkin-dependent mitophagy, thereby increasing the resistance of GC cells to cisplatin (25). In contrast, Wang et al. reported that GGT7 exerts a crucial tumor-suppressive effect in GC by inducing mitophagy and inhibiting reactive oxygen species (ROS) as well as the MAPK cascade (26). These findings indicate that the role of mitophagy in GC is intricate and multifaceted, necessitating further investigation into its potential as a therapeutic target.

DAZ-associated protein 1 (DAZAP1), an RNA-binding protein and a regulator of alternative splicing, is involved in mammalian development and tumorigenesis (27–30). It has been reported that DAZAP1 functions as a driver gene in recurrent mantle cell lymphoma (31). High DAZAP1 expression is associated with a poor prognosis in hepatocellular carcinoma (27, 32). However, Chen et al. reported that DAZAP1 functions as a tumor suppressor in esophageal squamous cell carcinoma (28). Moreover, DAZAP1 facilitates splicing control in MEK/ERK-regulated cell proliferation and migration, linking DAZAP1 to MEK inhibitors (30). While the various roles of DAZAP1 in carcinogenesis have been identified, its function in CSCs, particularly in how it maintains CSC biology by regulating metabolic and metabolic plasticity, remains unclear. Notably, emerging evidence suggests that DAZAP1 is a key regulator of mitochondrial energy dynamics. In oral squamous cell carcinoma, DAZAP1 regulates the splicing of cytochrome *c* oxidase 16 (COX16) pre-mRNA, boosting COX16 expression to enhance mitochondrial respiration and promote tumor progression (33). These findings highlight the role of DAZAP1 in regulating mitochondrial metabolism, which is closely linked to the metabolic plasticity vital for CSC survival. Based on these observations, we hypothesize that DAZAP1 may maintain CSCs by modulating the splicing of mitophagy-related transcripts, which are critical for mitochondrial quality control and metabolic flexibility. This hypothesis sets the foundation for exploring involvement of DAZAP1 in CSC metabolism, particularly in the context of GC.

In this study, we investigated the role and underlying mechanisms of DAZAP1 in GC. We found that DAZAP1 markedly promotes mitophagy and OXPHOS by binding to Unc-51–like autophagy activating kinase 1 (ULK1). The upregulation of DAZAP1 is crucial for maintaining the stemness of GC cells. Mechanistically, DAZAP1 regulates the alternative splicing and expression of ULK1, which in turn induces mitophagy, promotes OXPHOS, and maintains GC cell stemness. Our findings reveal the important role of DAZAP1 in regulating GC stemness and suggest that targeting DAZAP1 could represent a promising strategy for treating GC stemness.

## Results

### DAZAP1 is highly expressed in GC and correlates with worse prognosis.

**Figure 1:**
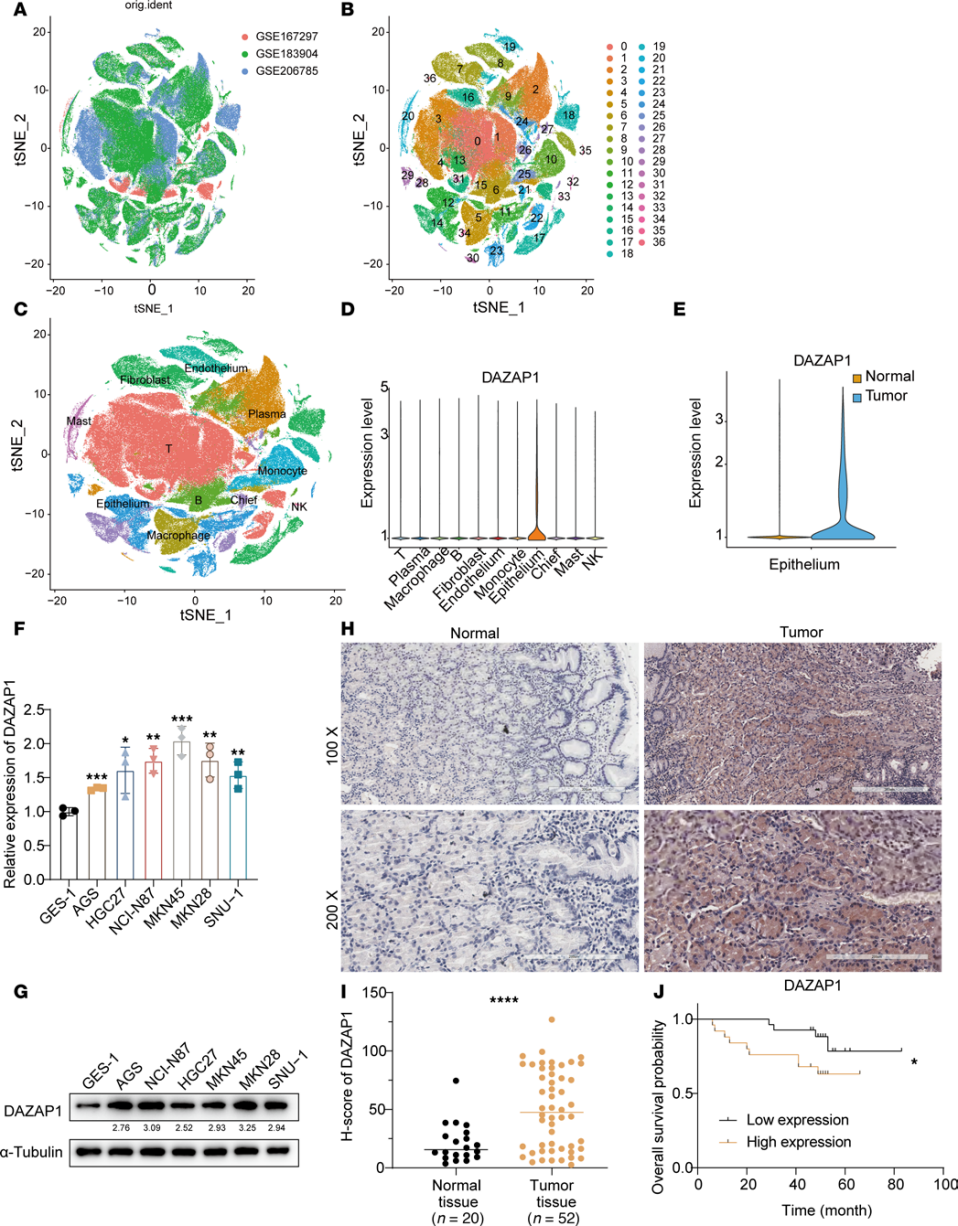
DAZAP1 expression and its association with prognosis in GC. (**A**) Integration of 3 scRNA-seq datasets (GSE167297, GSE183904, and GSE206785) yielded gene expression profiles from 280,130 cells across 58 primary GC samples and 39 normal tissues. (**B** and **C**) Cells were grouped into 37 clusters and further annotated into 11 cell types using the KNN algorithm. (**D** and **E**) DAZAP1 was predominantly expressed in epithelial cells, showing higher levels in malignant cells compared with normal tissues (*P* < 2 × 10^–16^). (**F** and **G**) Elevated DAZAP1 expression was observed in 6 GC cell lines relative to normal gastric mucosal epithelial cells. (**H**–**J**) IHC confirmed elevated DAZAP1 expression in GC (*n* = 52) compared with normal tissue (*n* = 20) (**H** and **I**), which correlated with poorer survival outcomes (*n* = 52) (**J**). Scale bars: 300 μm (top) and 200 μm (bottom). Data are presented as mean ± SD. Statistical analysis was by Wilcoxon’s rank-sum test (**E**), 1-way ANOVA followed by Tukey’s HSD post hoc test for multiple comparisons (**F**), log-rank (Mantel-Cox) test (**I**), or by unpaired Student’s *t* test (**J**). *P < 0.05, **P < 0.01, ***P < 0.001.

To elucidate the role of DAZAP1 in GC, we integrated 3 independent single-cell RNA sequencing (scRNA-seq) datasets (NCBI Gene Expression Omnibus [GEO] GSE167297, GSE183904, and GSE206785). We applied t-distributed stochastic neighbor embedding (t-SNE), a commonly used method for dimensionality reduction and clustering, to visualize high-dimensional scRNA-seq data. The t-SNE plot represents the data in a 2-dimensional space, where each point corresponds to a single cell, grouping similar cells based on their gene expression profiles. After quality control, we obtained gene expression profiles from 280,130 cells derived from 58 primary GC samples and 39 normal tissues (Figure 1A). By employing the KNN algorithm implemented in Seurat’s (version 4.4.0) FindNeighbors function (see Supplemental Methods; supplemental material available online with this article; https://doi.org/10.1172/jci.insight.175422DS1), these cells were categorized into 37 clusters and further annotated into 11 major cell types based on marker gene expression (Supplemental Figure 1A), including epithelial cells (nonmalignant and cancer cells), fibroblasts, endothelial cells, chief cells, T lymphocytes, B lymphocytes, plasma cells, natural killer (NK) cells, monocytes, macrophages, and mast cells (Figure 1, B and C, and Supplemental Figure 1B). The results showed that *DAZAP1* was predominantly expressed in epithelial cells, with higher levels in malignant cells compared with normal tissues (*P* < 2 × 10^–16^) (Figure 1, D and E). Additionally, we assessed DAZAP1 expression in various GC cell lines and normal gastric mucosal epithelial cells, finding that both *DAZAP1* transcript levels (Figure 1F) and DAZAP1 protein abundance (Figure 1G) were elevated in GC cells compared with normal cells. Immunohistochemical (IHC) analysis further confirmed that DAZAP1 was highly expressed in GC and that its elevated expression correlated with poorer survival outcomes (Figure 1, H–J). Analysis of the GEO database (GSE14210 and GSE15459 cohorts) indicated that GC patients with elevated *DAZAP1* mRNA had worse overall survival (Supplemental Figure 1C, *P* = 0.006) and progression-free survival (Supplemental Figure 1D, *P* = 0.007) compared with those with low expression. These findings suggest that DAZAP1 is associated with poor prognosis and adverse treatment outcomes in GC.

### DAZAP1 correlates with stemness in GC cells.

**Figure 2:**
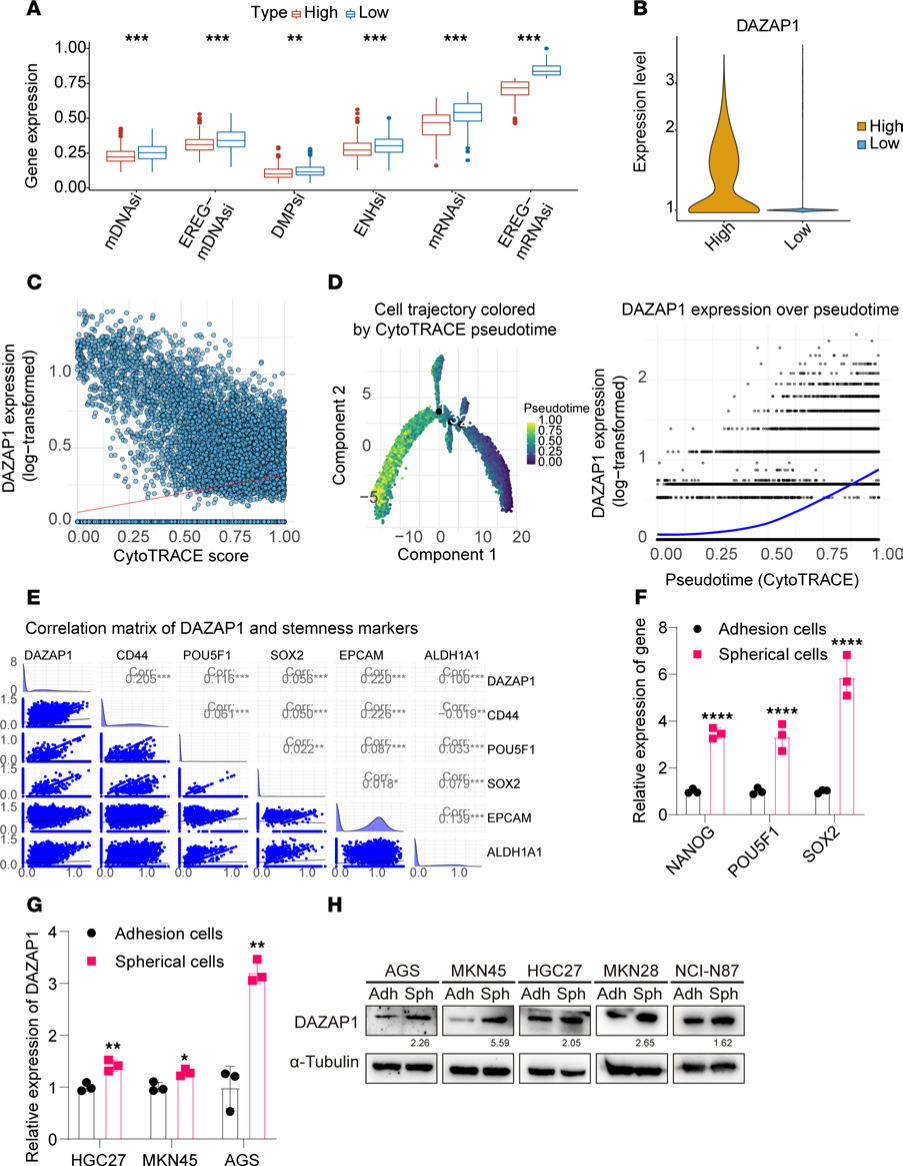
Elevated DAZAP1 expression in GCSCs. (**A**) Stemness indices of TCGA-STAD samples, calculated using the OCLR algorithm, were higher in the DAZAP1 high-expression group.(**B**) CytoTRACE analysis revealed higher DAZAP1 expression in high-stemness tumor cells (*P* < 2 × 10^–16^). (**C**) DAZAP1 expression was positively correlated with CytoTRACE scores in tumor epithelial cells (*r* = 0.38, *P* < 2 × 10^–16^). (**D**) Pseudotime analysis indicated that DAZAP1 expression increased progressively from undifferentiated to differentiated states. (**E**) DAZAP1 expression was positively correlated with the stem cell markers EPCAM and CD44. (**F** and **G**) Spheroid cultures enriched for GCSCs exhibited elevated levels of stemness genes and DAZAP1 mRNA compared with adherent cells. (**H**) Western blot analysis confirmed increased DAZAP1 protein levels in spheroid cells. Data are presented as mean ± SD. Statistical analysis was by unpaired Student’s *t* test (**A**, **F**, and **G**), Wilcoxon’s rank-sum test (**B**), or Spearman’s correlation analysis (**C** and **E**). *P < 0.05; **P < 0.01; ***P < 0.001; ****P < 0.0001.

To further explore the role of DAZAP1 in GCSCs, we calculated the stemness index of The Cancer Genome Atlas stomach adenocarcinoma (TCGA-STAD) samples using the one-class logistic regression (OCLR) algorithm (see Supplemental Methods). The results revealed that the stemness index (including mRNAsi, EREG-mRNAsi, mDNAsi, EREG-mDNAsi, DMPsi, and ENHsi scores) was markedly elevated in the high-DAZAP1-expression group (Figure 2A). Cytological tracing of regulatory activity in cancer evolution (CytoTRACE; see Supplemental Methods) predicts and quantifies the differentiation state of single cells to evaluate their differentiation potential (Supplemental Figure 1E). Cells were classified into high and low stemness groups based on the median CytoTRACE score, and the results demonstrated that *DAZAP1* mRNA was higher in high-stemness tumor cells (*P* < 2.2 × 10^–16^) (Figure 2B). In tumor epithelial cells, the CytoTRACE score was substantially and positively correlated with *DAZAP1* expression (*R* = 0.38, *P* < 2.2 × 10^–16^) (Figure 2C). Pseudotime analysis further revealed that *DAZAP1* expression progressively increased as cells transitioned from an undifferentiated state (rich in stemness characteristics) to a differentiated state, suggesting that DAZAP1 plays a crucial role in maintaining cell stemness (Figure 2D). Additionally, we evaluated the association of DAZAP1 with GCSC markers (such as *CD44*, *NANOG*, *POU5F1*, *SOX2*, *EPCAM*, *PROM1*, and *ALDH1A1*) and discovered that *DAZAP1* was positively correlated with *EPCAM* and *CD44* (Figure 2E).

To enrich GCSCs, we cultured GC cells in serum-free DMEM/F12 supplemented with basic fibroblast growth factor (b-FGF), epidermal growth factor (EGF), and 1× B27 supplement. The results indicated that the mRNA levels of stemness genes (such as *NANOG*, *POU5F1*, and *SOX2*) were elevated in spheroid cells compared with adherent cells (Figure 2F). Additionally, *DAZAP1* mRNA levels were elevated in spheroid cells compared with corresponding adherent tumor cells (Figure 2G). Western blot analysis demonstrated that DAZAP1 protein expression was upregulated in spheroid cells (Figure 2H). These findings suggest that elevated DAZAP1 expression in GC is closely associated with tumor cell stemness and poor prognosis, implying its potential role in promoting GCSC characteristics and tumor progression.

### DAZAP1 promotes proliferation, migration, and invasiveness of GC cells.

**Figure 3:**
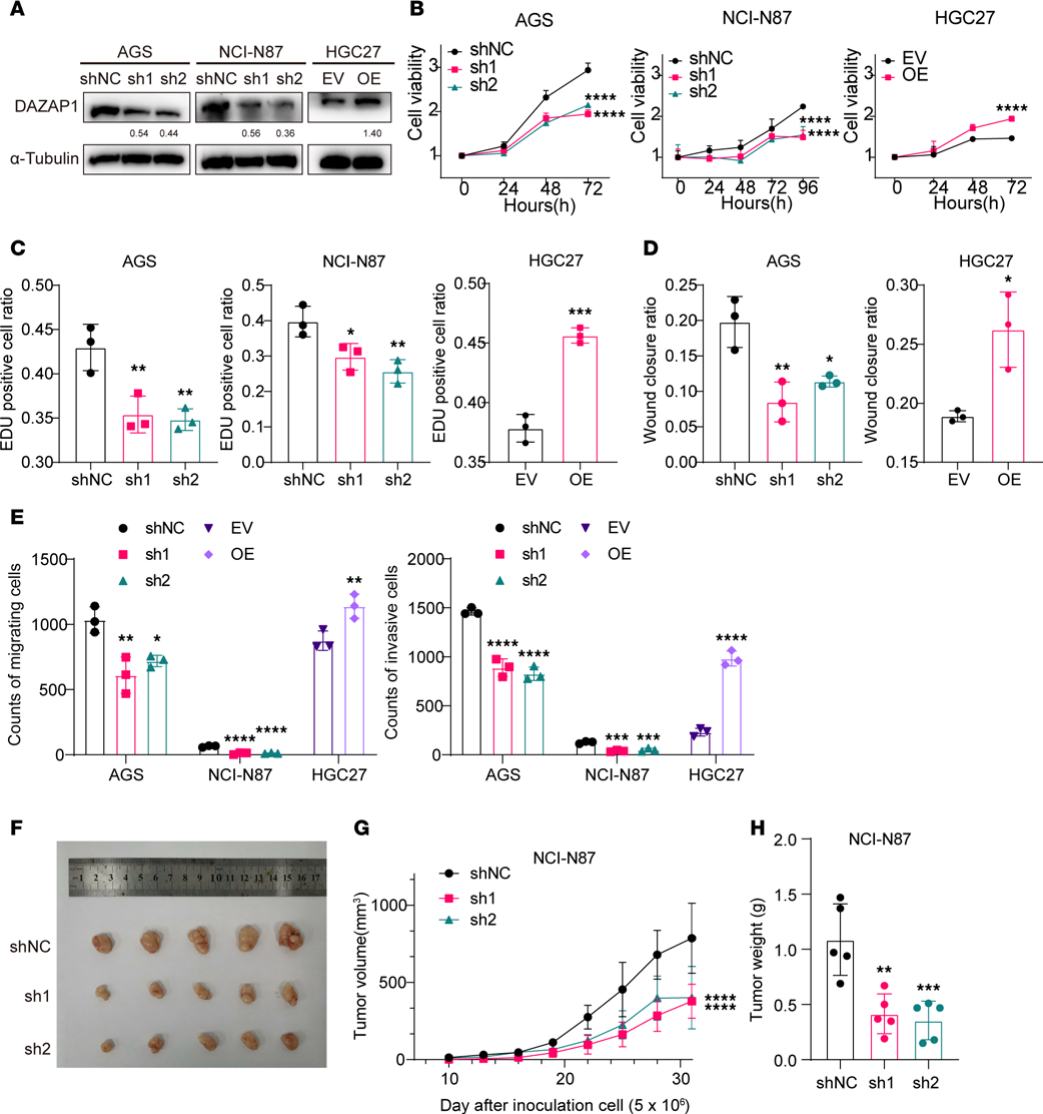
The impact of DAZAP1 on malignant phenotypes in GC. (**A**) DAZAP1 knockdown in AGS and NCI-N87 cells reduced its expression, while overexpression in HGC27 cells elevated DAZAP1 levels. EV, empty vector. (**B**) CCK8 assays demonstrated decreased cell viability with DAZAP1 knockdown and increased cell proliferation with its overexpression. (**C**) EdU assays confirmed that DAZAP1 enhances cell proliferation. (**D**) Wound healing assays demonstrated that DAZAP1 knockdown reduces cell motility, while DAZAP1 overexpression increases it. (**E**) Transwell assays showed that DAZAP1 knockdown significantly inhibited, while DAZAP1 overexpression enhanced, the migration and invasiveness of GC cells. (**F**–**H**) In vivo tumor formation assays with NCI-N87 cells revealed that DAZAP1 knockdown inhibited tumor growth rate and tumor weight (*n* = 5/group). Data are presented as mean ± SD. Statistical analysis was by 2-way ANOVA followed by Tukey’s HSD post hoc test for multiple comparisons (**B** and **G**), unpaired Student’s *t* test for comparisons between 2 groups and 1-way ANOVA followed by Tukey’s HSD post hoc test for comparisons among 3 groups (**C**–**E**), or 1-way ANOVA followed by Tukey’s HSD post hoc test (**H**). *P < 0.05; **P < 0.01; ***P < 0.001; ****P < 0.0001.

To further elucidate the role of DAZAP1 in regulating GC stemness, we knocked down *DAZAP1* in GC cell lines AGS and NCI-N87 using 2 independent short hairpin RNAs (shRNAs). Compared with the negative control, DAZAP1 expression was substantially reduced in stable sh-DAZAP1–transfected cells (Figure 3A). A stable DAZAP1-overexpressing (DAZAP1-OE) model was established in HGC27 cells, demonstrating a marked increase in DAZAP1 expression in DAZAP1-OE cells (Figure 3A). CCK8 assays indicated that DAZAP1 depletion reduced cell viability, while DAZAP1 overexpression promoted cell proliferation (Figure 3B). EdU assays further confirmed the proliferative effect of DAZAP1, with DAZAP1 knockdown inhibiting cell proliferation and DAZAP1 overexpression markedly enhancing it (Figure 3C and Supplemental Figure 2A). Gene set enrichment analysis (GSEA) of scRNA-seq data revealed that DAZAP1 is highly enriched in various metastatic signaling pathways and stemness-related pathways, such as ALONSO_METASTASIS_UP, WINNEPENNINCKX_MELANOMA_METASTASIS_UP, PECE_MAMMARY_STEM_CELL_UP, WONG_EMBRYONIC_STEM_CELL_CORE, and BHATTACHARYA_EMBRYONIC_STEM_CELL (Supplemental Figure 2B). Wound healing assays demonstrated that DAZAP1 knockdown reduced cell motility, whereas DAZAP1 overexpression increased it (Figure 3D and Supplemental Figure 2C). Transwell assays further indicated that DAZAP1 knockdown markedly inhibited the migration and invasiveness of GC cells, while DAZAP1 overexpression enhanced these abilities (Figure 3E and Supplemental Figure 2D).

In vivo experiments using NCI-N87 cells for tumor formation assays were conducted to assess the impact of DAZAP1 on tumor growth. Compared with the control group, the results showed that DAZAP1 knockdown substantially inhibited the tumor growth rate and weight (Figure 3, F–H).

### DAZAP1 regulates stem cell–like properties in GC cells.

**Figure 4:**
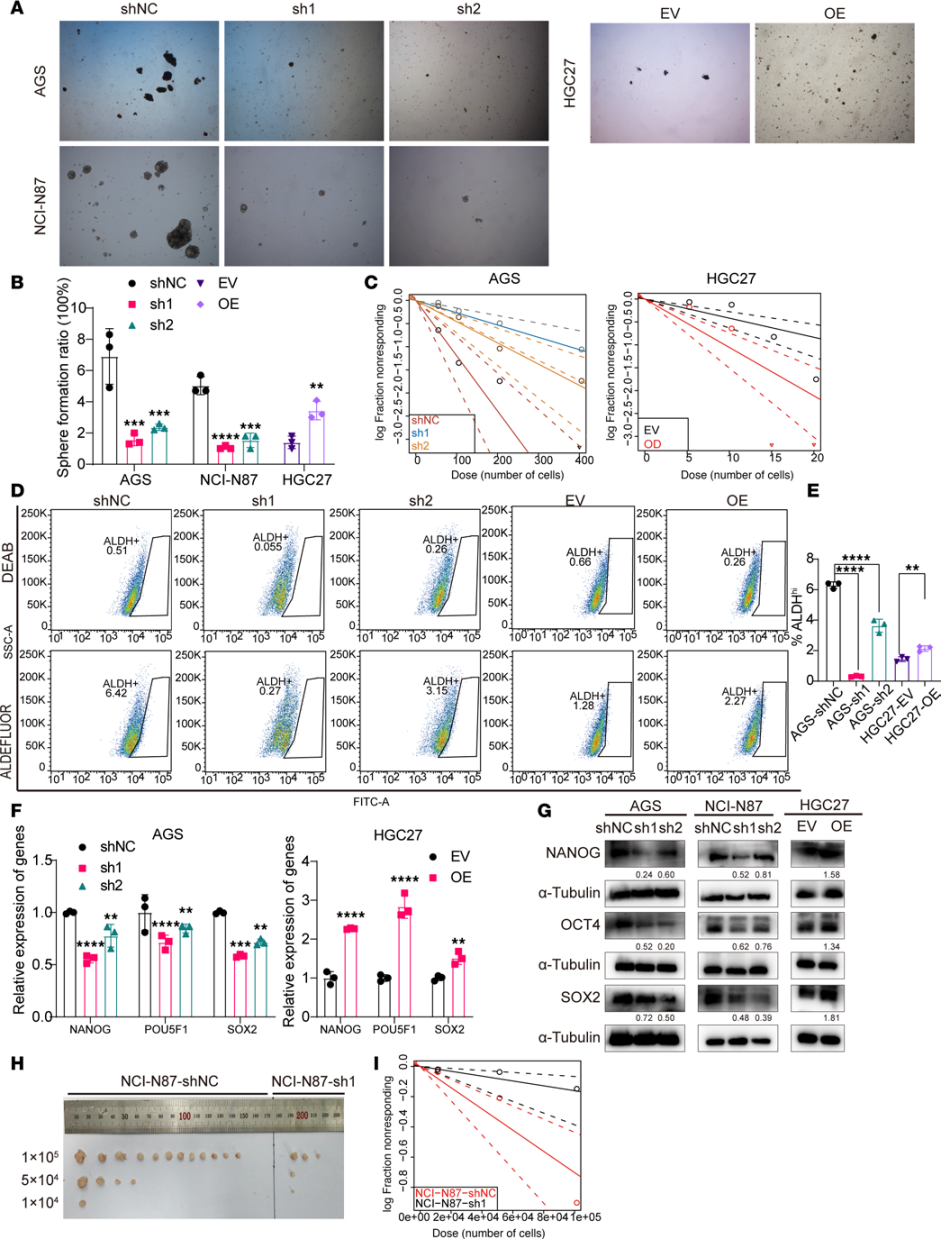
DAZAP1 promotes stemness properties in GC. (**A** and **B**) Sphere formation assays indicated that DAZAP1 knockdown reduced the number of spheres formed, while overexpression increased sphere-forming ability. (**C**) Extreme limiting dilution assay (ELDA) indicated that DAZAP1 knockdown decreases self-renewal capacity, while DAZAP1 overexpression enhances it. (**D** and **E**) ALDEFLUOR assays showed a decrease in ALDH-high cells with DAZAP1 knockdown and an increase with DAZAP1 overexpression. (**F** and **G**) Quantitative PCR (qPCR) and Western blot analyses revealed that DAZAP1 overexpression upregulated stemness markers SOX2, OCT4, and NANOG, while knockdown downregulated these markers. (**H** and **I**) In vivo ELDA using DAZAP1-knockdown NCI-N87 cells demonstrated a substantially reduced tumorigenic capacity, requiring higher cell numbers to form detectable tumors compared with control cells. All quantitative data are presented as the mean ± SD of at least 3 independent experiments. Statistical analysis was by unpaired Student’s *t* test for comparisons between 2 groups and 1-way ANOVA followed by Tukey’s HSD post hoc test for comparisons among 3 groups (**B**, **E**, and **F**). *P < 0.05; **P < 0.01; ***P < 0.001; ****P < 0.0001.

To determine the role of DAZAP1 in promoting stemness, we assessed sphere formation in cells with altered DAZAP1 expression. DAZAP1 knockdown reduced the number of spheres formed, whereas DAZAP1 overexpression enhanced sphere formation in GC cells (Figure 4, A and B). Extreme limiting dilution assay (ELDA) showed that the self-renewal capacity of DAZAP1-knockdown cells decreased, whereas that of DAZAP1-OE cells increased (Figure 4C). Pairwise tests for differences in stem cell frequencies are shown in Supplemental Table 1 Additionally, ALDEFLUOR assays were performed to detect aldehyde dehydrogenase (ALDH) activity. The results showed a substantial decrease in the proportion of ALDH-high cells in DAZAP1-silenced cells, whereas the number of ALDH-high cells increased in DAZAP1-OE cells (Figure 4, D and E).

Quantitative PCR (qPCR) and Western blot analyses indicated that DAZAP1 overexpression markedly upregulated the expression levels of GC stemness markers SOX2, OCT4, and NANOG, while DAZAP1 knockdown downregulated these markers (Figure 4, F and G). To further validate the impact of DAZAP1 on stemness characteristics, we knocked down DAZAP1 in NCI-N87 cells and conducted in vivo ELDA. We injected DAZAP1-knockdown NCI-N87 cells and control cells at different cell concentrations (1 × 10^4^, 5 × 10^4^, and 1 × 10^5^ cells) into immunodeficient mice and observed the tumor formation rates. The results showed that DAZAP1-knockdown cells had substantially reduced tumorigenic ability at all concentrations compared with the control group, requiring a higher number of cells to form detectable tumors (Figure 4, H and I). Pairwise tests for differences in stem cell frequencies are shown in Supplemental Table 2.

These results suggest that DAZAP1 knockdown substantially reduces the self-renewal capacity and tumorigenicity of GCSCs, further confirming the crucial role of DAZAP1 in maintaining tumor stemness. In summary, these data indicate that DAZAP1 not only markedly promotes the proliferation and migration of GC cells but also drives tumor progression and invasion by enhancing stemness characteristics.

### DAZAP1 enhances OXPHOS activity.

**Figure 5:**
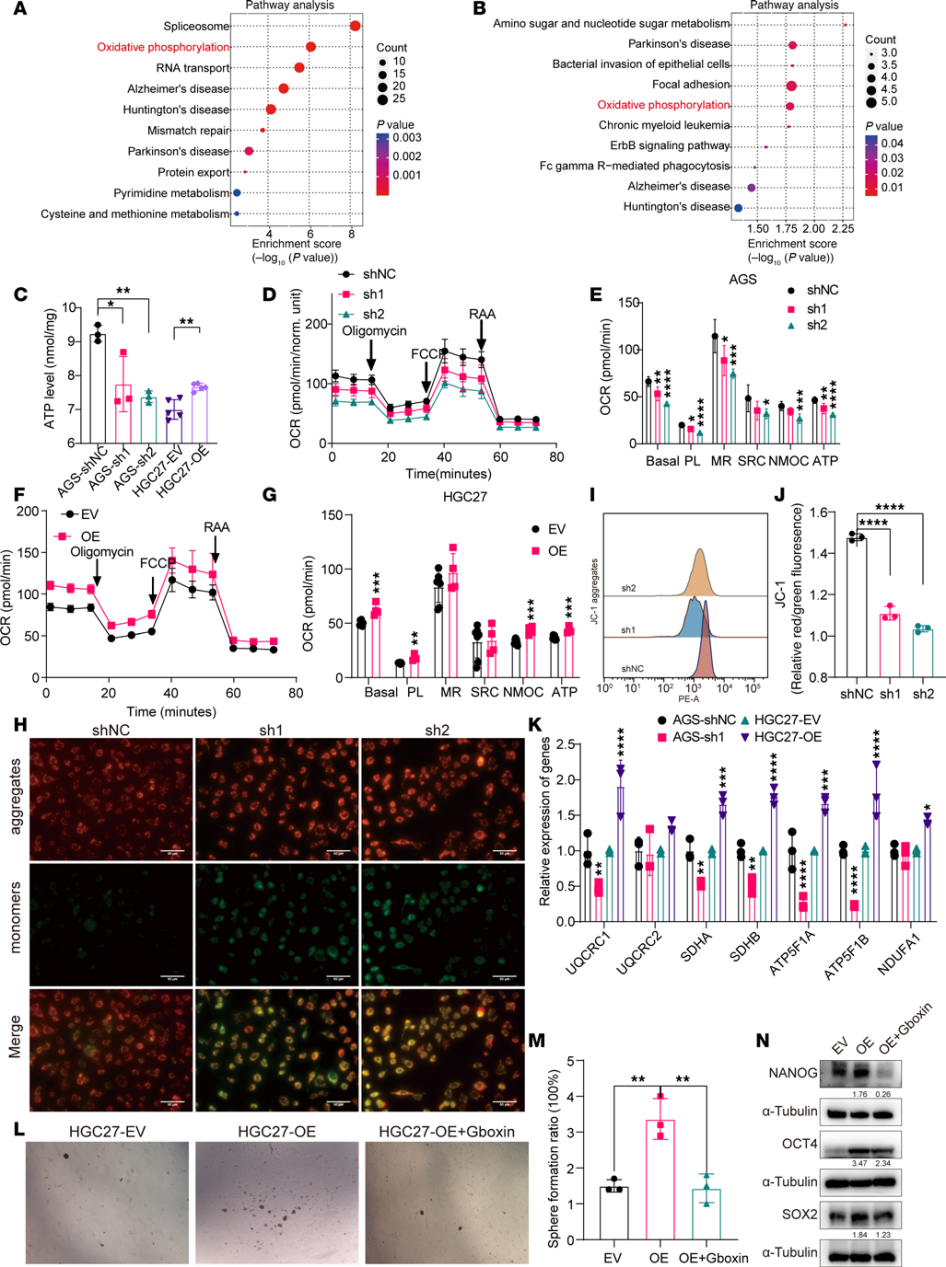
DAZAP1 enhances OXPHOS activity and GC cell stemness. (**A** and **B**) KEGG functional enrichment analysis revealed that differentially expressed genes were markedly involved in OXPHOS processes following DAZAP1 knockdown or overexpression. (**C**) Measurement of ATP content indicated reduced ATP levels with DAZAP1 knockdown and increased levels with overexpression. (**D**) Seahorse XF Analyzer data confirmed that DAZAP1 knockdown decreased OXPHOS activity, as evidenced by a reduction in OCR. (**E**) Specific inhibitor and substrate assays revealed suppressed basal OCR, spare respiratory capacity, and ATP production in DAZAP1-knockdown cells, with no change in proton leak. PL, proton leak; MR, maximal respiration; SRC, spare respiratory capacity; NMOC, non-mitochondrial oxygen consumption. (**F** and **G**) DAZAP1 overexpression increased basal OCR, proton leak, spare respiratory capacity, and ATP production. (**H**–**J**) JC-1 assay demonstrated decreased mitochondrial membrane potential in DAZAP1-knockdown cells, indicated by a lower red/green fluorescence ratio. Scale bars: 50 μm. (**K**) Gene expression analysis showed a decrease in the expression of OXPHOS complex subunit genes in DAZAP1-knockdown cells and an increase in these genes with overexpression. (**L** and **M**) Sphere formation assays demonstrated that the OXPHOS inhibitor Gboxin weakened the sphere-forming capacity enhanced by DAZAP1 overexpression. (**N**) Western blot analysis revealed that the elevation of stemness markers (OCT4, NANOG, and SOX2) induced by DAZAP1 overexpression was counteracted by the OXPHOS inhibitor Gboxin. Quantitative data are expressed as the mean ± SD from a minimum of 3 independent experiments. Statistical analysis by unpaired Student’s *t* test (HGC27-EV vs. HGC27-OE) for comparisons between 2 groups and 1-way ANOVA followed by Tukey’s HSD post hoc test (AGS-shNC vs. AGS-sh1 and AGS-sh2) for comparisons among 3 groups (**C**), 1-way ANOVA followed by Tukey’s HSD post hoc test (**E**, **J**, and **M**), or unpaired Student’s *t* test (**G** and **K**). *P < 0.05; **P < 0.01; ***P < 0.001; ****P < 0.0001.

To investigate the role of DAZAP1 in GC, we initially conducted a proteomic analysis to examine the functional changes in cells following DAZAP1 knockdown or overexpression, aiming to elucidate its biological functions. Kyoto Encyclopedia of Genes and Genomes (KEGG) functional enrichment analysis showed that, compared with the control group, differentially expressed genes in DAZAP1-knockdown (Figure 5A) or DAZAP1-OE (Figure 5B) cells were markedly involved in the regulation of OXPHOS processes. Volcano plots further revealed substantial changes in the expression levels of genes related to the OXPHOS pathway, including *SDHB*, *TIMM50*, and *NDUFA2*, in DAZAP1-silenced or -OE cells (Supplemental Figure 3, A and B). To evaluate mitochondrial function, ATP levels were quantified using a luminometric assay. Additionally, the oxygen consumption rate (OCR) was measured using a Seahorse XF96 Analyzer with the XF Cell Mito Stress Test Kit. Mitochondrial membrane potential was assessed with JC-1 staining. The expression of OXPHOS-related genes was assessed by qPCR. The results indicated that DAZAP1 knockdown substantially reduced ATP levels, while DAZAP1 overexpression increased ATP levels (Figure 5C). Measurement of the OCR using the Seahorse XF Analyzer further confirmed that DAZAP1 knockdown substantially inhibited OXPHOS activity, as evidenced by the decreased OCR (Figure 5D). Evaluation of OXPHOS complex function using specific inhibitors and substrates revealed that basal OCR, spare respiratory capacity, proton leak, and ATP production were all suppressed in DAZAP1-knockdown cells (Figure 5E). Conversely, DAZAP1 overexpression substantially increased basal OCR, proton leak, spare respiratory capacity, and ATP production (Figure 5, F and G). Moreover, JC-1 assay results showed that DAZAP1 knockdown increased the JC-1 monomer fluorescence intensity, thus markedly decreasing the JC-1 aggregate/monomer (red/green) ratio (Figure 5, H–J), further confirming DAZAP1’s critical role in maintaining mitochondrial membrane potential and OXPHOS activity. Additional gene expression analysis indicated that DAZAP1 knockdown decreased the expression of OXPHOS complex subunit genes, including *UQCRC1*, *UQCRC2*, *SDHA*, *SDHB*, *ATP5F1A*, *ATP5F1B*, and *NDUFA1*, while DAZAP1 overexpression increased the expression of these genes, suggesting that DAZAP1 enhances cellular OXPHOS levels (Figure 5K). Finally, we treated GC cells with the OXPHOS uncoupler Gboxin and performed sphere formation assays and Western blot analysis of stemness markers. Sphere formation assays showed that DAZAP1 overexpression substantially increased the sphere-forming ability of GCSCs, while this ability was markedly suppressed following the addition of the OXPHOS inhibitor, highlighting the importance of OXPHOS levels in GCSCs (Figure 5, L and M). Furthermore, Western blot analysis revealed that DAZAP1 overexpression substantially upregulated the expression of stemness markers, including OCT4, NANOG, and SOX2, while their expression markedly decreased following Gboxin treatment (Figure 5N).

In conclusion, these data indicate that DAZAP1 substantially enhances OXPHOS activity by regulating mitochondrial function, thereby influencing the stemness and sphere-forming ability of GCSCs.

### DAZAP1 enhances OXPHOS by inducing mitophagy to maintain stemness in GC cells.

To explore the role of DAZAP1 in GCSCs, we further investigated its potential mechanisms, focusing on its high expression and enhancement of mitochondrial OXPHOS activity. Studies suggest that mitophagy plays a crucial role in enhancing OXPHOS function. Mitophagy is a process that maintains mitochondrial quality and function by selectively degrading damaged or unnecessary mitochondria, thereby preventing the generation of harmful ROS and boosting OXPHOS activity. This process supports cellular metabolism and energy production by preserving mitochondrial function (34, 35). Additionally, the interaction between mitophagy and mitochondrial biogenesis is critical for maintaining mitochondrial homeostasis and contributes to enhanced OXPHOS efficiency (36). Therefore, we hypothesized that DAZAP1 may enhance OXPHOS and maintain stemness in GC cells by inducing mitophagy.

**Figure 6:**
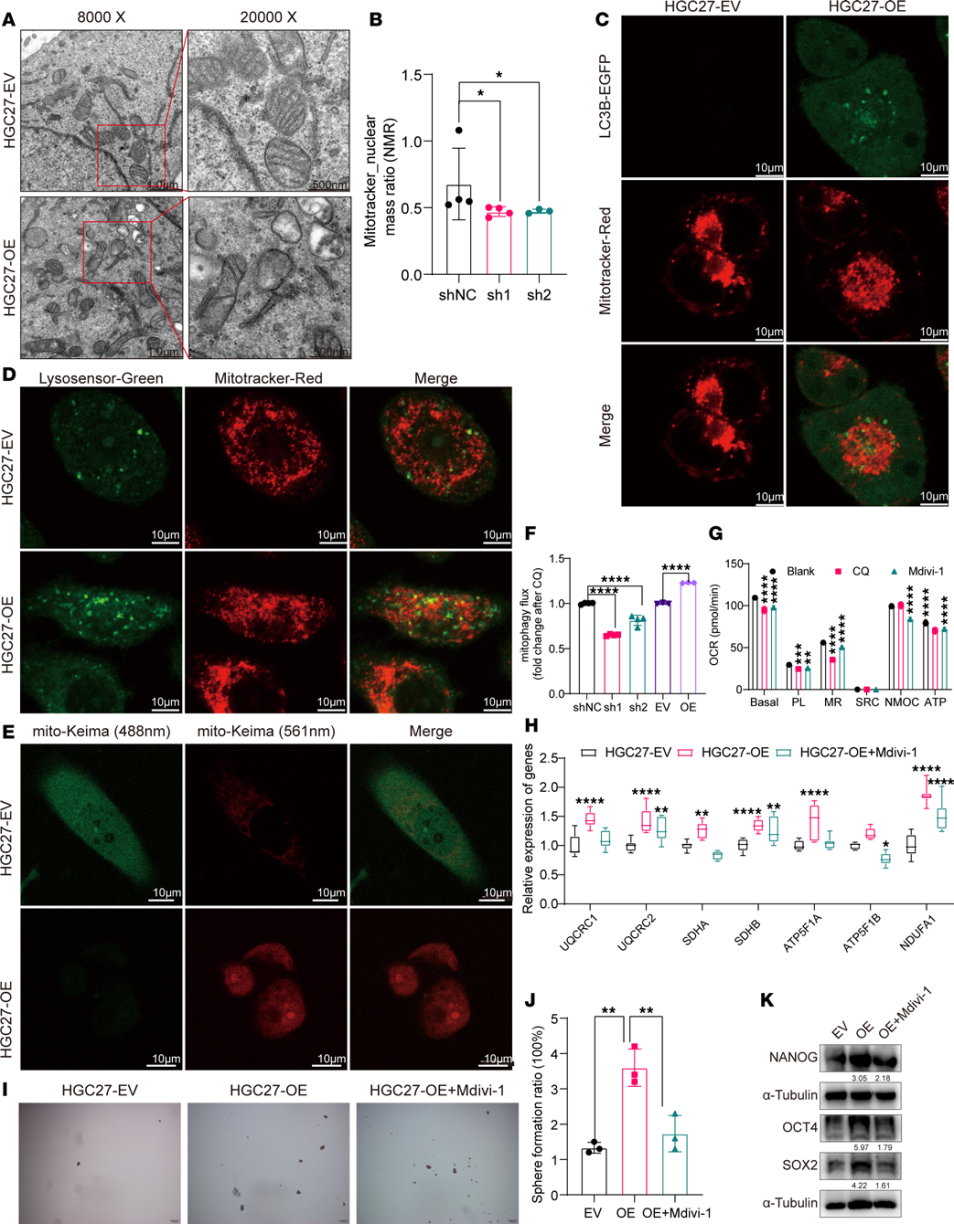
DAZAP1 enhances OXPHOS and maintains cell stemness in GC by inducing mitophagy. (**A**) TEM images show an increased number of mitophagosomes in DAZAP1-overexpressing (DAZAP1-OE) cells, indicating induced mitophagy. Scale bars: 1 μm (left) and 500 nm (right). (**B**) Confocal microscopy using MitoTracker Red reveals mitochondrial fragmentation and a reduced mitochondria-to-nucleus ratio following DAZAP1 knockdown (*n* = 3). (**C**) Immunofluorescence (IF) analysis shows increased colocalization of mitochondria with LC3B in DAZAP1-OE cells, indicating enhanced mitophagy. (**D**) DAZAP1 overexpression results in increased colocalization of LC3B with lysosomes, promoting autophagosome-lysosome fusion. (**E**) Mito-Keima plasmid labeling demonstrates increased mitophagy in DAZAP1-OE cells. Scale bars (**C**–**E**): 10 μm. (**F**) MitoTracker Deep Red (MTDR) dye analysis confirms impaired mitophagy in DAZAP1-knockdown cells and enhanced mitophagy in DAZAP1-OE cells. (**G**) Oxygen consumption rate (OCR) analysis shows reduced basal OCR, proton leak, and ATP production in GC cells treated with the autophagy inhibitor chloroquine (CQ) and the mitophagy inhibitor Mdivi-1, indicating the role of mitophagy in regulating OXPHOS. (**H**) Gene expression analysis reveals that Mdivi-1 restores the expression of OXPHOS complex subunit genes (*UQCRC1*, *UQCRC2*, *SDHA*, *SDHB*, *ATP5F1A*, *ATP5F1B*, and *NDUFA1*) elevated by DAZAP1 overexpression. (**I** and **J**) Sphere formation assay shows that Mdivi-1 treatment substantially reduces the sphere-forming ability of DAZAP1-OE cells. (**K**) Western blot analysis demonstrates that Mdivi-1 treatment decreases the expression of stemness markers SOX2, OCT4, and NANOG in DAZAP1-OE cells. Quantitative data are presented as the mean ± SD from at least 3 independent experiments. Statistical analysis by 1-way ANOVA followed by Tukey’s HSD post hoc test for multiple comparisons (**B**, **G**, **H**, and **J**) or unpaired Student’s *t* test (EV vs. OE) for comparisons between 2 groups and 1-way ANOVA followed by Tukey’s HSD post hoc test for multiple comparisons (shNC vs. sh1 and sh2) (**F**). *P < 0.05; **P < 0.01; ***P < 0.001; ****P < 0.0001.

To validate this hypothesis, we first examined the effect of DAZAP1 on mitophagy. Mitophagy levels were evaluated using several experimental methods. Transmission electron microscopy (TEM) was used to observe mitochondrial morphology and identify autophagic structures. In addition, mitochondria-targeted Keima (mito-Keima) detection was employed to track mitophagy in live cells, while colocalization of LC3B with mitochondria and colocalization of lysosomes with mitochondria were assessed via immunofluorescence (IF). The fluorescence ratio of MitoTracker Deep Red (MTDR) in the presence of chloroquine (CQ) versus without CQ was used to assess the mitophagic flux. TEM images showed an increased number of mitophagosomes in DAZAP1-OE cells compared with control cells (Figure 6A), suggesting that DAZAP1 may induce mitophagy. Using MTDR to observe mitochondrial quality, confocal microscopy revealed mitochondrial fragmentation and a reduction in the mitochondria-to-nucleus ratio following DAZAP1 gene knockdown (Figure 6B and Supplemental Figure 4A). RNA-seq analysis suggested that mitophagy might be a downstream signaling pathway of DAZAP1 (Supplemental Figure 4B). IF analysis indicated increased colocalization of mitochondria with LC3B in DAZAP1-OE cells, suggesting enhanced mitophagy (Figure 6C). Additionally, DAZAP1 overexpression led to increased colocalization of LC3B with lysosomes, promoting autophagosome-lysosome fusion in GC cells (Figure 6D). Furthermore, using a mito-Keima plasmid to label mitophagy, results showed increased mitophagy in DAZAP1-OE cells (Figure 6E). MTDR dye analysis confirmed that DAZAP1 knockdown impaired mitophagy, whereas DAZAP1 overexpression enhanced it (Figure 6F). These results suggest that DAZAP1 induces mitophagy.

Moreover, we confirmed the role of mitophagy in regulating OXPHOS. Treatment with the autophagy inhibitor CQ and the mitophagy inhibitor Mdivi-1 both reduced the OCR of GC cells, as evidenced by decreased basal OCR, proton leak, and ATP production (Figure 6G). Gene expression analysis further revealed that the mitophagy inhibitor Mdivi-1 restored the expression of OXPHOS complex subunit genes, including *UQCRC1*, *UQCRC2*, *SDHA*, *SDHB*, *ATP5F1A*, *ATP5F1B*, and *NDUFA1*, which had been elevated by DAZAP1 overexpression (Figure 6H). This suggests that DAZAP1 enhances cellular OXPHOS levels.

To investigate the role of mitophagy in DAZAP1-regulated cell stemness, we conducted a sphere formation assay. The results showed that the mitophagy inhibitor Mdivi-1 markedly reduced the sphere-forming ability of DAZAP1-OE cells (Figure 6, I and J). Western blot analysis also revealed that the expression levels of stemness markers SOX2, OCT4, and NANOG markedly decreased following Mdivi-1 treatment (Figure 6K).

In conclusion, these findings demonstrate that DAZAP1 enhances OXPHOS by inducing mitophagy, thereby maintaining the stemness of GC cells.

### DAZAP1 regulates alternative splicing of ULK1 RNA to activate mitophagy.

**Figure 7:**
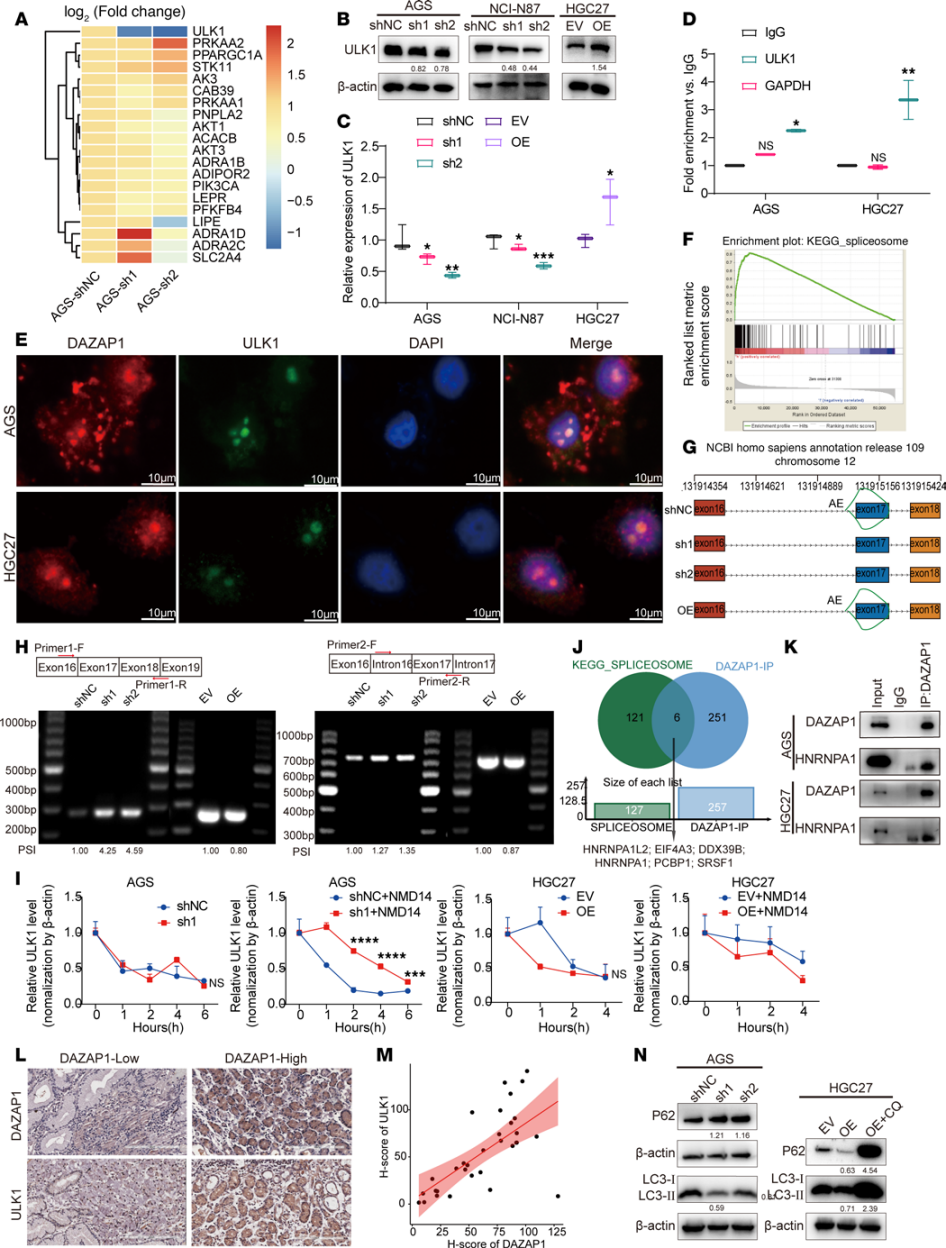
DAZAP1 regulates alternative splicing of *ULK1* to activate mitophagy. (**A**) PCR array results demonstrated a marked reduction in *ULK1* expression in shDAZAP1 cells. (**B** and **C**) qPCR and Western blot analyses indicated that DAZAP1 overexpression upregulated ULK1 mRNA and protein levels, while knockdown downregulated them. (**D**) RIP-qPCR analysis confirmed DAZAP1 binding to *ULK1* RNA. (**E**) FISH-IF staining indicated colocalization of DAZAP1 and *ULK1* RNA. Scale bars: 10 μm. (**F**) GSEA demonstrated substantial enrichment of the spliceosome pathway in DAZAP1-OE cells. (**G**) RNA-seq analysis identified alternative splicing sites within *ULK1* RNA, showing that DAZAP1 promotes exon 17 skipping. (**H**) PCR and agarose gel electrophoresis confirmed DAZAP1 regulation of *ULK1* RNA splicing. (**I**) Actinomycin D assays demonstrated that DAZAP1 increases *ULK1* mRNA stability by reducing the production of premature termination codons and subsequent nonsense-mediated decay (NMD). (**J**) Co-IP experiments confirmed that DAZAP1 binds to several alternative splicing factors, including HNRNPC, DDX39B, HNRNPA1L2, HNRNPM, HNRNPA1, EIF4A3, PCBP1, RBMX, and HNRNPA3. (**K**) Co-IP confirmed the interaction between DAZAP1 and HNRNPA1, reinforcing DAZAP1’s role in alternative splicing regulation. (**L** and **M**) IHC analysis revealed a positive correlation between DAZAP1 and ULK1 expression levels (*r* = 0.69, *P* = 1.9 × 10^–5^), *n* = 32. Scale bars: 200 μm. (**N**) Western blot analysis indicated that DAZAP1 overexpression upregulated mitophagy markers LC3B and P62, while knockdown downregulated them. Quantitative data are shown as the mean ± SD from a minimum of 3 independent experiments. Statistical analysis by unpaired Student’s *t* test (EV vs. OE) for comparisons between 2 groups and 1-way ANOVA followed by Dunnett’s multiple-comparison test (shNC vs. sh1 and sh2) (**C**), 1-way ANOVA followed by Dunnett’s multiple-comparison test (**D**), 2-way ANOVA followed by Šidák’s multiple-comparison test (**I**), or Spearman’s correlation analysis (**M**). *P < 0.05; **P < 0.01; ***P < 0.001; ****P < 0.0001. NS indicates no statistically significant difference.

To determine the mechanism by which DAZAP1 activates mitophagy, we utilized a PCR array containing genes related to mitochondrial dynamics. The results showed that ULK1 exhibited the most substantial reduction in shDAZAP1 cells (Figure 7A). ULK1 is a critical component in mitophagy, forming a complex with FIP200, ATG13, and ATG101 to initiate the mitophagic response and interact with selective autophagy receptors such as P62/SQSTM1, ensuring the encapsulation and degradation of damaged mitochondria (37–39).

We first examined the effect of DAZAP1 on ULK1 expression. qPCR and Western blot analyses revealed that DAZAP1 overexpression substantially upregulated the mRNA and protein levels of ULK1, whereas DAZAP1 knockdown markedly downregulated ULK1 expression (Figure 7, B and C). RNA immunoprecipitation (RIP)–qPCR analysis demonstrated that DAZAP1 could bind to *ULK1* mRNA (Figure 7D), and fluorescence in situ hybridization–IF (FISH-IF) staining showed colocalization of DAZAP1 and *ULK1* mRNA (Figure 7E). GSEA indicated substantial enrichment of the spliceosome pathway in DAZAP1-OE cells (Figure 7F). Mass spectrometry analysis also supported the association of DAZAP1 with alternative splicing events. RNA-seq analysis further revealed alternative splicing sites within *ULK1* mRNA (Figure 7G). PCR and agarose gel electrophoresis confirmed that DAZAP1 regulates exon 17 skipping in *ULK1* mRNA (Figure 7H). We found that retention of exon 17 in *ULK1* led to aberrant mRNAs containing premature termination codons, which are degraded by the nonsense-mediated mRNA decay (NMD) pathway in vivo (40, 41). Up-frameshift 1 (UPF1) is a core RNA helicase involved in mRNA NMD (42). Using actinomycin D to assess *ULK1* mRNA stability, we observed that DAZAP1 knockdown alone did not substantially affect *ULK1* stability, but stability increased with the use of the UPF1 inhibitor NMD14. Conversely, DAZAP1 overexpression did not enhance *ULK1* stability, but its stability decreased upon the addition of NMD14. These results suggest that DAZAP1 increases normal *ULK1* expression by regulating its alternative splicing, reducing the production of aberrant mRNAs containing premature termination codons (Figure 7I).

As an RNA-binding protein and alternative splicing regulator, DAZAP1 modulates various mRNA splicing events. For instance, in esophageal squamous cell carcinoma, DAZAP1 knockdown led to marked changes in alternative splicing events (28). In multiple myeloma cells, DAZAP1 promoted cell proliferation by enhancing the alternative splicing of KITLG mRNA, activating the ERK signaling pathway (43). DAZAP1 primarily regulates alternative splicing by promoting exon skipping (43, 44). Moreover, coimmunoprecipitation (Co-IP) experiments confirmed that DAZAP1 can bind to several alternative splicing factors, including HNRNPC, DDX39B, HNRNPA1L2, HNRNPM, HNRNPA1, EIF4A3, PCBP1, RBMX, and HNRNPA3, exerting its splicing function (Figure 7J). Notably, DAZAP1 has been reported as a splicing enhancer, with its interaction with splicing factors HNRNPA1 and HNRNPA2 being a key determinant of exon exclusion (45, 46). Our Co-IP experiments further confirmed that DAZAP1 binds to HNRNPA1, reinforcing its pivotal role in alternative splicing regulation (Figure 7K).

IHC analysis was performed to evaluate the protein expression levels of DAZAP1 and ULK1. Representative microscopic images of DAZAP1 and ULK1 are shown in Figure 7L, demonstrating a positive correlation between their IHC scores (*r* = 0.69, *P* = 1.9 × 10^–5^) (Figure 7M). Furthermore, ULK1 levels in human GC samples were evaluated and correlated with clinical outcomes. As shown in Supplemental Figure 5A, ULK1 expression was substantially upregulated in tumor tissues compared with normal tissues (*P* = 0.0391). However, Kaplan-Meier analysis revealed no association between ULK1 expression and overall survival in patients with GC (Supplemental Figure 5, B and C). Notably, ULK1 expression was markedly correlated with clinical stage, with higher levels observed in patients with advanced-stage (stage III+IV) GC (Supplemental Figure 5D, *P* = 0.0154). To further verify the role of ULK1 in DAZAP1-regulated mitophagy, we conducted functional experiments. Western blot analysis showed that DAZAP1 overexpression substantially upregulated the protein levels of mitophagy markers LC3B and P62, whereas these markers were downregulated in DAZAP1-knockdown cells (Figure 7N).

In summary, DAZAP1 enhances mitophagy by regulating the alternative splicing of ULK1, thereby maintaining the stemness of GC cells.

### ULK1-mediated mitophagy is essential for DAZAP1-induced GC cell stemness maintenance.

**Figure 8:**
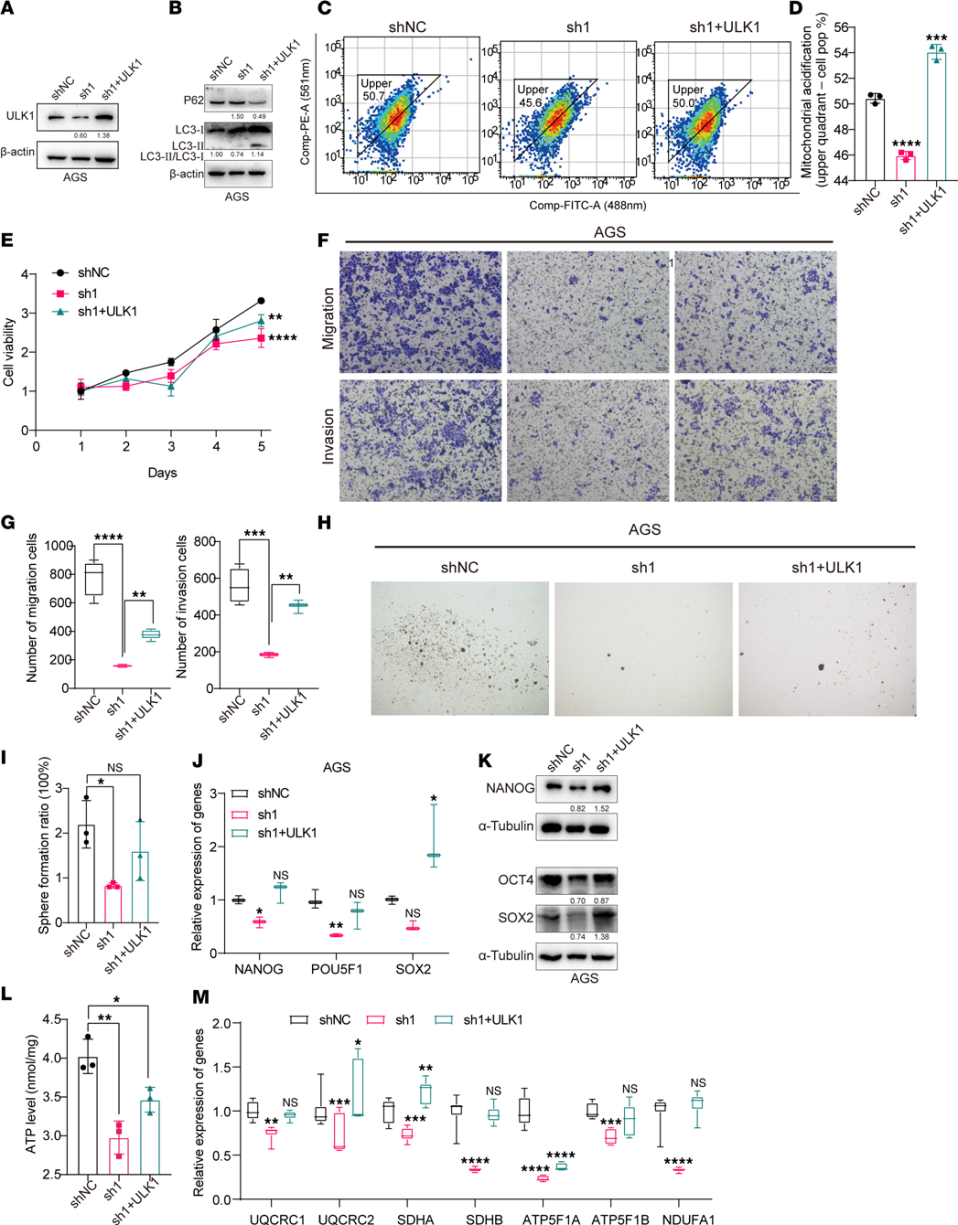
ULK1-mediated mitophagy is essential for DAZAP1-induced stemness in GC cells. (**A**) Western blot analysis showed that ULK1 protein levels markedly decrease following DAZAP1 knockdown, while reintroducing ULK1 restores its expression. (**B**) Western blot analysis demonstrated that ULK1 overexpression rescues the expression of autophagy markers LC3B and P62 in DAZAP1-knockdown cells. (**C** and **D**) Mito-Keima labeling indicated that ULK1 overexpression partially restores mitophagy in DAZAP1-knockdown cells. (**E**) CCK8 assay showed that ULK1 overexpression partially restores cell proliferation in DAZAP1-knockdown cells. (**F** and **G**) Transwell migration assay indicated that ULK1 overexpression rescues the cell migration impaired by DAZAP1 knockdown. Scale bars: 100 μm. (**H** and **I**) Sphere formation assay demonstrated that ULK1 overexpression increases the number of spheres, counteracting the inhibitory effect of DAZAP1 knockdown. (**J** and **K**) qPCR and Western blot analyses showed that restoring ULK1 in DAZAP1-knockdown cells rescues the expression of stemness markers SOX2, OCT4, and NANOG. (**L**) ATP production assay indicated that ULK1 overexpression partially restores OXPHOS activity in DAZAP1-knockdown cells. (**M**) Gene expression analysis showed that ULK1 overexpression increases the expression of key OXPHOS complex subunit genes (*UQCRC1*, *UQCRC2*, *SDHA*, *SDHB*, *ATP5F1A*, *ATP5F1B*, and *NDUFA1*), indicating enhanced OXPHOS in DAZAP1-knockdown cells. Quantitative data are shown as the mean ± SD from a minimum of 3 independent experiments. Statistical analysis by 1-way ANOVA followed by Dunnett’s multiple-comparison test (**D**, **G**, **I**, **J**, **L**, and **M**) or 2-way ANOVA followed by Tukey’s HSD post hoc test for multiple comparisons (**E**). *P < 0.05; **P < 0.01; ***P < 0.001; ****P < 0.0001. NS indicates no statistically significant difference.

To evaluate the role of ULK1 in the regulation of GCSC characteristics by DAZAP1, we used shRNA to knock down ULK1 in DAZAP1-OE cells and reintroduced properly spliced ULK1 after DAZAP1 knockdown. ULK1 protein levels substantially decreased following DAZAP1 knockdown, while reintroducing ULK1 in DAZAP1-knockdown cells restored ULK1 protein levels (Figure 8A). Additionally, overexpressing ULK1 in DAZAP1-knockdown cells rescued the expression of autophagy markers LC3B and P62 (Figure 8B). To further assess the impact of ULK1 on mitophagy, we utilized a mito-Keima plasmid to label mitochondria undergoing mitophagy. The results indicated that ULK1 overexpression partially restored mitophagy in DAZAP1-knockdown cells (Figure 8, C and D). Proliferation analysis using CCK8 assays showed that reintroducing ULK1 in DAZAP1-knockdown cells partially restored cell proliferation (Figure 8E). Similarly, Transwell migration assays demonstrated that ULK1 overexpression partially rescued cell migration impaired by DAZAP1 knockdown (Figure 8, F and G). Sphere formation assays revealed that ULK1 overexpression substantially increased the number of spheres, whereas DAZAP1 knockdown markedly inhibited this effect (Figure 8, H and I). Furthermore, qPCR and Western blot analyses showed that restoring ULK1 in DAZAP1-knockdown cells reinstated the expression of GC stemness markers SOX2, OCT4, and NANOG, which had been suppressed by DAZAP1 knockdown (Figure 8, J and K). To explore the role of ULK1 in DAZAP1-mediated regulation of OXPHOS, we overexpressed ULK1 in DAZAP1-knockdown cells. The results showed that ULK1 overexpression partially restored ATP production in these cells (Figure 8L). Additionally, gene expression analysis indicated that ULK1 overexpression substantially increased the expression of key OXPHOS complex subunit genes, including *UQCRC1*, *UQCRC2*, *SDHA*, *SDHB*, *ATP5F1A*, *ATP5F1B*, and *NDUFA1*, suggesting that ULK1 enhances OXPHOS activity in DAZAP1-knockdown cells (Figure 8M).

In conclusion, these data indicate that DAZAP1 enhances mitophagic activity by regulating the expression of the mitophagy-related gene *ULK1*, thereby promoting OXPHOS activity and cellular stemness. This suggests that ULK1-mediated mitophagy is essential for DAZAP1-induced maintenance of stemness characteristics, providing a potential therapeutic target.

## Discussion

CSCs are often considered the primary drivers of tumor recurrence and metastasis, leading to treatment failure and poor prognosis across various malignancies. Therefore, identifying important factors that enhance stemness is of substantial clinical importance. In this study, we provide evidence that high DAZAP1 expression is substantially associated with stemness characteristics in GC cells, highlighting its crucial role in maintaining the state of GCSCs, a link that has not been extensively studied. DAZAP1 not only promotes the proliferation, migration, and invasiveness of GC cells but also exacerbates tumor progression by enhancing cellular stemness characteristics. These findings have been thoroughly validated in both in vivo and in vitro experiments. Knockdown of DAZAP1 markedly reduced the proliferation and stemness characteristics of GC cells, while DAZAP1 overexpression markedly enhanced these properties. Additionally, high DAZAP1 expression is closely associated with the upregulation of stemness markers, including SOX2, OCT4, and NANOG, further confirming DAZAP1’s role in enhancing and maintaining GCSC characteristics.

RNA-seq and mass spectrometry analyses revealed that DAZAP1’s potential functions are primarily related to mitochondrial pathways, suggesting the need to further explore its effects on mitochondrial morphology and function. Our experimental results indicate that DAZAP1 can upregulate the expression of OXPHOS-related genes, increase ATP production, and enhance the OCR of cells. Treatment with the OXPHOS inhibitor Gboxin, which uncouples the mitochondrial respiratory chain, inhibited the DAZAP1-induced enhancement of stemness characteristics in GC cells, as evidenced by reduced sphere formation and downregulation of stemness-related gene expression. These results indicate that OXPHOS is necessary for DAZAP1-mediated stemness. This finding aligns with existing literature, which has widely demonstrated that enhanced OXPHOS promotes CSC stemness characteristics. For instance, in breast cancer, cells with elevated OXPHOS activity exhibit greater stemness and metastatic potential (47). Similarly, research on colon cancer has shown that high OXPHOS activity is closely associated with the maintenance of tumor stemness, emphasizing its crucial role in CSCs (48).

Our study further discovered that DAZAP1 promotes OXPHOS activity by inducing mitophagy. TEM and IF analyses showed an increased number of autophagosomes in DAZAP1-OE cells, with enhanced colocalization of mitochondria with LC3B and lysosomes, indicating that DAZAP1 increases mitophagy. Previous studies have demonstrated that mitophagy enhances OXPHOS activity by selectively eliminating damaged or dysfunctional mitochondria, preventing the production of harmful reactive oxygen species (35, 36). Li et al. further reported that mitophagy is crucial for supporting the switch of drug-tolerant persister cancer cells to OXPHOS and maintaining mitochondrial homeostasis (49). By inducing mitophagy, DAZAP1 effectively clears damaged mitochondria, maintains mitochondrial health and function, and thereby supports high levels of OXPHOS activity.

Notably, mitophagy is essential for the self-renewal and differentiation of acute myeloid leukemia stem cells and plays a critical role in maintaining the stemness phenotype of malignant tumors, such as liver cancer stem cells (50, 51). Feng et al. reported that mitophagy-mediated mitochondrial fission promotes the stemness of bone marrow mesenchymal stem cells (23). Inhibition of mitophagy using the inhibitor Mdivi-1 substantially reduced the stemness characteristics of GC cells, as evidenced by decreased sphere formation and self-renewal capacity. These findings suggest that mitophagy is crucial for DAZAP1-mediated maintenance of stemness, uncovering interactions between DAZAP1, mitophagy, and GCSCs that remain poorly understood.

Importantly, this study reveals that DAZAP1 activates mitophagy by regulating the alternative splicing of *ULK1*. As a key regulator of mitophagy, ULK1’s expression and function are influenced by DAZAP1-mediated alternative splicing. Specifically, DAZAP1 promotes exon 17 skipping in *ULK1*, preventing the production of aberrant mRNA with premature termination codons, thus increasing the normal expression and function of *ULK1*. ULK1-mediated phosphorylation of Parkin facilitates an early step in mitophagy (38). We found that DAZAP1 overexpression upregulated the levels of p-Parkin, P62, and LC3B, and the DAZAP1-induced OXPHOS and stemness characteristics depended on ULK1’s expression and function. The promotive effects of DAZAP1 overexpression on stemness characteristics were reversed by ULK1 knockdown in both in vitro and in vivo models. Conversely, reintroducing ULK1 in DAZAP1-knockdown cells partially restored OXPHOS activity and stemness characteristics. These results strongly suggest that ULK1 is essential for DAZAP1-induced GC cell stemness. These findings not only expand our understanding of DAZAP1’s functions but also provide a therapeutic insight targeting ULK1-mediated mitophagy.

In summary, this study elucidates the critical functions and mechanisms of DAZAP1 in GC. DAZAP1 promotes the maintenance and progression of GCSCs by enhancing OXPHOS activity through the induction of mitophagy. Given DAZAP1’s pivotal role in GC, future research should focus on developing therapeutic strategies targeting DAZAP1 and its downstream pathways to improve prognosis and treatment outcomes for patients with GC. This discovery identifies critical therapeutic targets for GC, advancing the development of targeted therapies.

## Methods

Further information can be found in Supplemental Methods. A comprehensive list of reagents and experimental kits utilized in this study is available in Supplemental Table 3.

### Sex as a biological variable

Male mice were used in this study due to the higher incidence and mortality rates of GC observed in males compared with females, as documented in epidemiological studies (52). Research suggests that sex hormones, particularly the protective effects of estrogen in females, and differences in immune responses may contribute to this male predominance (53). Although only male mice were used, we anticipate that the mechanistic findings are relevant to both sexes, as the fundamental pathways involved in GC progression are applicable to both male and female patients.

For human tissue specimens, sex was not considered a selection criterion, as the acquisition process did not include sex-based selection. Therefore, the findings from the human tissue analysis are expected to be applicable to both sexes.

### Cell culture and transfection

The gastric mucosal epithelial cell line GES-1 and GC cell lines AGS, NCI-N87, HGC27, MKN45, MKN28, and SNU-1 were obtained from Cellcook Biotech Co., Ltd. All cell lines underwent short tandem repeat authentication to confirm their identity. All cells were cultured in Dulbecco’s modified Eagle medium (DMEM; Gibco) supplemented with 10% fetal bovine serum (FBS) and 1% penicillin/streptomycin, maintained at 37°C in a 5% CO_2_ atmosphere. The shRNA sequences targeting DAZAP1 were CCCAGGAGCGATAACAGTAAA (sh-DAZAP1-1) and CCAGGAGCGATAACAGTAAAT (sh-DAZAP1-2), while those used to knock down ULK1 were ACAUCGAGAACGUCACCAAGU (sh-ULK1-1) and CCUGGUUAUGGAGUACUGCAA (sh-ULK1-2). The shRNA plasmids were designed and synthesized by IGE Biotechnology. The overexpression plasmid carrying the human DAZAP1 sequence (DAZAP1-OE) was obtained from Tsingke Biotechnology Co., Ltd. Plasmids for LC3B and mito-Keima were synthesized by Wuhan Miaoling Bioscience and Technology Co., Ltd. Lentiviruses containing scrambled sequences (shCtrl) and empty vectors (Ctrl) were used as controls following the manufacturer’s instructions. GC cells were transfected with the lentiviral vectors, and DAZAP1-knockdown or -OE cells were selected using 2 μg/mL puromycin for 1 week to establish stable cell lines. ULK1-knockdown cell lines were selected with 100 μg/mL G418 for 1 week, as they carried the neo-resistance gene.

### Sphere formation assay

Sphere formation is a widely accepted method for enriching CSCs (54, 55). GC cells were digested into a single-cell suspension at a density of 1 × 10^5^ cells/mL. For the assay, serum-free DMEM/F12 supplemented with 20 ng/mL b-FGF, 20 ng/mL EGF, 1× B27 supplement, and 4% bovine serum albumin was used. A total of 1,000 GC cells were seeded per well into ultra-low-adherence 24-well plates containing 3 mL of complete DMEM/F12 and cultured for 14 days. Spheres were monitored daily, and images were captured using microscopy on day 14. The diameter of the spheres (>50 μm) was measured for statistical analysis.

### RNA-seq

Total RNA was extracted from cell or tissue samples using TRIzol reagent (Thermo Fisher Scientific). The quality and concentration of the RNA were assessed using the NanoDrop 2000 (Thermo Fisher Scientific) and the Agilent 2100 Bioanalyzer (Agilent Technologies), ensuring that the OD260/280 ratio was between 1.8 and 2.0. The RNA-seq library preparation included mRNA enrichment, fragmentation, cDNA synthesis, end repair, A-tailing, adapter ligation, and PCR amplification. The quality of the libraries was evaluated on the Agilent 2100 Bioanalyzer, and sequencing was performed on the DNBSEQ-T7 platform. Quality control of the raw data was conducted using FastQC (https://www.bioinformatics.babraham.ac.uk/projects/fastqc/), including adapter trimming and removal of low-quality reads and undetermined bases. The high-quality reads were aligned to the human reference genome (GRCh38/hg38) using Hisat2 software (http://daehwankimlab.github.io/hisat2/). The KEGG functional enrichment analysis was performed on the Sanger website, utilizing the latest KEGG pathway gene annotations (56).

### Alternative splicing analysis

For alternative splicing analysis, the replicate Multivariate Analysis of Transcript Splicing (rMATS) software (version 4.1.2; https://rnaseq-mats.sourceforge.net/) was used to identify 5 types of splicing events: skipped exons, alternative 5′ splice sites, alternative 3′ splice sites, retained introns, and mutually exclusive exons. The splicing events were considered significant when the false discovery rate (FDR) was less than 0.05, and the absolute inclusion level difference (|IncLevelDifference|) was greater than 0.1. Specifically, the skipping of exon 17 of *ULK1* was identified as an alternative splicing event. To validate this splicing event, primers for PCR were designed using the Primer-BLAST website (https://www.ncbi.nlm.nih.gov/tools/primer-blast/). Primers were designed to span from exon 16 to intron 18 of the *ULK1* gene. Primer 1 was designed to amplify the region of *ULK1* mRNA containing exon 17, with a cross-exon design, while Primer 2-R spanned both intron 17 and exon 17. If exon 17 was skipped, PCR amplification would not occur. Total RNA was extracted using TRIzol, as previously above. After measuring the RNA concentration, 5,000 ng of RNA was used to synthesize single-stranded cDNA using the HiScript II 1st Strand cDNA Synthesis Kit (R201, Vazyme), followed by amplification using 2× Rapid Taq Master Mix (P222-01, Vazyme). PCR products were analyzed by electrophoresis in a 1.5% agarose gel, run at 120 V for 40 minutes, and imaged under UV light. All primer sequences used in this study are listed in Supplemental Table 4. By aligning the PCR products to the reference genome (GRCh38/hg38 for human), the intron and exon regions were identified, confirming that exon 17 of *ULK1* was skipped (event type: SE; event_start-event_end: 131915101–131915231; 131915083–131915231).

### Mitophagy assays

#### TEM.

Mitochondrial shape and autophagosomes were observed using TEM. Cells were fixed with 2.5% glutaraldehyde at 4°C overnight, washed with 0.1 mol/L phosphate buffer, postfixed with 1% osmium tetroxide, and embedded with epoxy resin Spurr for ultrathin sectioning. Mitochondrial ultrastructure was visualized at various magnifications using TEM.

#### Mito-Keima reporter assay.

Mitochondrial autophagy flux was quantitatively assessed using the pH-sensitive mito-Keima reporter system. Cells were transfected with the mito-Keima plasmid following the transfection protocol described above. After transfection, mitochondrial autophagic flux was analyzed using either flow cytometry or confocal microscopy, both of which involved excitation with 488 nm (neutral pH, non-lysosomal mitochondria) and 561 nm (acidic pH, lysosome-engulfed mitochondria) lasers. Flow cytometric data were analyzed using FlowJo v10.8 software (BD Biosciences). Mitochondrial autophagic flux was calculated as the ratio of the 561 nm to 488 nm fluorescence intensity, and the data were normalized to the untreated control group.

#### IF colocalization.

For colocalization of LC3B with mitochondria, cells were transfected with the LC3B plasmid. Mitochondria were labeled using MitoTracker Red CMXRos (Thermo Fisher Scientific, M7512; 100 nM final concentration), diluted 1:10,000 in serum-free medium prewarmed to 37°C. Cells at 70%–80% confluence were incubated with the staining solution for 45 minutes under 5% CO_2_ at 37°C. For colocalization of lysosomes with mitochondria, after mitochondrial staining, lysosomes were stained with LysoSensor Green DND-189 (Thermo Fisher Scientific, L7535; diluted 1:1000 in PBS) for 30 minutes under identical culture conditions. After staining, fluorescence was preserved using an anti-fade reagent, and cells were imaged using a laser confocal microscope.

#### Mitophagy flux.

Mitophagy levels were detected using the MTDR dye (Thermo Fisher Scientific, M22426). The MTDR staining buffer, at a final concentration of 100 nM, was prepared using serum-free medium prewarmed to 37°C. GC cells were cultured in the staining buffer for 45 minutes at 37°C. After incubation, the cells were washed twice with PBS, digested, centrifuged, and resuspended in 300 μL PBS. Mitophagy flux is defined as the ratio of MTDR fluorescence in the presence of CQ (20 μM, 24 hours) to fluorescence without the inhibitor, normalized to the corresponding value for control cells (55). Carbonyl cyanide *m*-chlorophenyl hydrazone (CCCP), which induces mitophagy, was used as a positive control, with cells treated at a low concentration (10 μM) for 12 hours.

### OXPHOS assays

#### ATP assay.

Intracellular ATP levels were measured using an ATP assay kit (Beyotime) according to the manufacturer’s instructions. Cells (approximately 2 × 10^5^) were lysed and centrifuged at 4°C for 5 minutes at 12,000*g*. An aliquot of 100 μL ATP working buffer was added to 96-well plates and incubated at room temperature (25°C) for 5 minutes. Equal volumes of samples and standards were then added to measure ATP concentration using a luminometer.

#### Mitochondrial energy metabolism.

The OCR was measured using a Seahorse XF96 Analyzer and an XF Cell Mito Stress Test Kit (Agilent Technologies). Approximately 0.8 × 10^4^ GC cells per well were cultured overnight in 96-well XF cell culture microtiter plates with DMEM. The cells were treated with 1 μM oligomycin, 1.5 μM carbonyl cyanide-*p*-trifluoromethoxyphenylhydrazone (FCCP), and 0.5 μM rotenone/antimycin A. Data were analyzed using the Seahorse Mito Stress Test Report Generator.

#### Mitochondrial membrane potential.

JC-1 staining was used to assess mitochondrial membrane potential. Cells were incubated with the fluorescent mitochondrial probe JC-1 (1 μg/mL) and examined by fluorescence microscopy and flow cytometry, following the manufacturer’s instructions. JC-1 forms aggregates (indicative of intact mitochondria) at high transmembrane potentials, fluorescing red under confocal microscopy, whereas it remains in its monomeric form at low transmembrane potentials, exhibiting green fluorescence. The red-to-green fluorescence ratio of JC-1 indicates the mitochondrial membrane potential, with a decrease in red fluorescence and an increase in green fluorescence signifying a reduction in the mitochondrial membrane potential.

### RIP analysis

Cells were grown to 90% confluence in a 10-cm dish, with approximately 1 × 10^7^ cells. After washing twice with PBS, the cells were collected and lysed in a cell lysis buffer on ice containing RNase and protease inhibitors to prevent RNA degradation. After 2 hours, the cell lysate was centrifuged, and the supernatant was transferred to a new RNase-free tube. IP was performed using 5 μg of anti-DAZAP1 antibody or standard rabbit immunoglobulin G (IgG) as a control, both bound to magnetic beads after incubation at 4°C for 6–8 hours. Then, 300 μL of cell lysate was added to the tubes separately and incubated overnight at 4°C. A 10% (v/v) aliquot of the cell lysate was used as the input group. After washing the IP complex, the precipitate was digested with proteinase K, and RNA was extracted for subsequent analysis. The purified RNA was analyzed by quantitative real-time PCR (qRT-PCR) to confirm the presence of the target gene *ULK1*. Details regarding all antibodies and their working concentrations are provided in Supplemental Table 5.

### FISH-IF

We performed colocalization analysis of *ULK1* RNA and DAZAP1 protein using FISH-IF double staining. Tumor cells (2 × 10^4^) were seeded into 24-well plates and incubated overnight. The cells were fixed with 4% paraformaldehyde and permeabilized with 0.5% Triton X-100 at 4°C for 10 minutes. Prehybridization solution was added dropwise, and the cells were incubated at 37°C for 1 hour. After removing the buffer, *ULK1* probe hybridization buffer was added and incubated overnight at 37°C. Following washing with 1× SSC buffer, 200 μL of diluted primary antibody (DAZAP1, 1:100) was added to each well and incubated overnight at 4°C. Fluorescent secondary antibodies were then applied for 1 hour at room temperature (25°C) while protecting the cells from light, followed by staining with DAPI for 10 minutes. Finally, the appropriate wavelength was selected for observation and image acquisition using laser confocal microscopy.

### Animal experiments

In this study, 5- to 6-week-old male BALB/c nude mice (Guangdong Sijia Jingda Biotechnology Co., Ltd.) were used for in vivo experiments, with mice randomly allocated to different groups. To investigate the proliferative effect of DAZAP1, NCI-N87 GC cells with DAZAP1 knockdown and control cells were injected into the left thighs of each mouse at a concentration of 5 × 10^6^ cells per mouse. Tumor volume was measured every 3 days for 1 month using calipers and calculated with the formula, (length × width^2^)/2. After 4 weeks, the mice were euthanized via cervical dislocation, and the tumors were excised, weighed, and photographed.

An in vivo ELDA was also conducted to evaluate the stemness mediated by DAZAP1. NCI-N87 GC cells with DAZAP1 knockdown and control cells were injected into both thighs of each mouse at increasing concentrations (1 × 10^4^, 5 × 10^4^, and 1 × 10^5^ cells), with at least 10 mice per concentration. The experiment lasted for 2 months. After 8 weeks, the mice were euthanized by cervical dislocation, and tumor tissues were extracted, weighed, and photographed. Tumor numbers in each group were analyzed using the ELDA website (https://bioinf.wehi.edu.au/software/elda/). Tumor growth measurements and data analysis were performed blind to group assignments.

Five- to six-week-old male BALB/c nude mice were housed under specific pathogen–free conditions at the Experimental Animal Center of the School of Medicine, South China University of Technology (Guangzhou, China). All mice were maintained in a controlled environment (22°C ± 2°C and 50% ± 10% humidity) with a 12-hour light/dark cycle, provided ad libitum access to standard chow and sterile water. Prior to experiments, mice were screened for health status, and only those without abnormal behavior or clinical signs of disease were included.

### Statistics

Statistical analysis was conducted using R software (version 4.0) and GraphPad Prism (version 8.3). High- and low-expression groups were defined based on the median value of DAZAP1 expression. For 2-group comparisons, unpaired, 2-tailed *t* tests (for parametric data) or Wilcoxon’s rank-sum tests (for nonparametric data) were used. Multiple group comparisons were analyzed by 1-way ANOVA with Tukey’s honestly significant difference (HSD) post hoc test or 2-way ANOVA where appropriate. Survival analysis was performed using log-rank (Mantel-Cox) tests, while correlations were assessed using Spearman’s method. All experiments were repeated at least 3 times independently, with quantitative data expressed as mean ± SD. In the box-and-whisker plots, the center line represents the median, the box edges represent the 25th (Q1) and 75th (Q3) percentiles, the whiskers extend to the smallest and largest values within 1.5 times the interquartile range (IQR) from the lower and upper quartiles, respectively, and dots beyond the whiskers represent outliers. A *P* value of less than 0.05 was considered significant.

### Study approval

The patients with GC in our study were diagnosed with pathologically confirmed gastric adenocarcinoma through surgical resection, and complete follow-up information was available for all cases. The acquisition of human tissue specimens for this study was approved by the Ethics Committee of Guangzhou First People’s Hospital (approval no. K-2021-140), with informed consent obtained from all donors. All animal experiments were conducted in accordance with the approval from the Laboratory Animal Ethics Committee, School of Medicine, South China University of Technology (approval date: August 24, 2020).

### Data availability

The single-cell data used in this study are available in the NCBI GEO database (GSE167297, GSE183904, and GSE206785). Gastric adenocarcinoma expression data and clinical information were obtained from the TCGA-STAD project and the GEO database (GSE14210 and GSE15459). The next-generation RNA-seq data generated in this study have been deposited in the ArrayExpress database under the accession number E-MTAB-14963. All quantitative data points underlying the graphs and statistical analyses are provided in the Supporting Data Values file submitted with this manuscript. All other data supporting the findings of this study are included in the article and its supplemental material, or are available from the corresponding authors upon reasonable request.

## Author contributions

LL and ZXX conceived and designed the study. PZ and WW performed the experiments and analyzed the data. PZ and HX designed and performed the animal experiments, analyzed the resulting data, and interpreted the findings. YZ contributed to the statistical analysis of experimental data and assisted in animal model validation. QP participated in data collection, performed bioinformatics analysis, and supported the interpretation of results. LL, PZ, and WW drafted the manuscript. GL and ZXX critically reviewed the manuscript. LL and GL supervised the study. All authors approved the final version of the submitted manuscript.

## Supplementary Material

undefined

Supplemental data

Unedited blot and gel images

Supporting data values

## Version 1 05/22/2025

Electronic publication

## References

[1] Sung H (2021). Global Cancer Statistics 2020: GLOBOCAN estimates of incidence and mortality worldwide for 36 cancers in 185 countries. CA Cancer J Clin.

[2] Bray F (2024). Global cancer statistics 2022: GLOBOCAN estimates of incidence and mortality worldwide for 36 cancers in 185 countries. CA Cancer J Clin.

[3] Chen Y (2022). Anti-PD-1 combined with targeted therapy: theory and practice in gastric and colorectal cancer. Biochim Biophys Acta Rev Cancer.

[4] Jin X (2022). Recent progress and future perspectives of immunotherapy in advanced gastric cancer. Front Immunol.

[5] Liu S (2014). Breast cancer stem cells transition between epithelial and mesenchymal states reflective of their normal counterparts. Stem Cell Reports.

[6] Wu Y (2023). Molecular mechanisms of tumor resistance to radiotherapy. Mol Cancer.

[7] Brungs D (2016). Gastric cancer stem cells: evidence, potential markers, and clinical implications. J Gastroenterol.

[8] Nishikawa S (2013). Aldehyde dehydrogenase high gastric cancer stem cells are resistant to chemotherapy. Int J Oncol.

[9] Takebe N (2015). Targeting notch, hedgehog, and Wnt pathways in cancer stem cells: clinical update. Nat Rev Clin Oncol.

[10] Hu Y (2016). Therapeutic efficacy of cancer stem cell vaccines in the adjuvant setting. Cancer Res.

[11] Lu L (2015). Cancer stem cell vaccine inhibits metastases of primary tumors and induces humoral immune responses against cancer stem cells. Oncoimmunology.

[12] Viale A (2014). Oncogene ablation-resistant pancreatic cancer cells depend on mitochondrial function. Nature.

[13] Tanabe A, Sahara H (2020). The metabolic heterogeneity and flexibility of cancer stem cells. Cancers (Basel).

[14] Kuntz EM (2017). Targeting mitochondrial oxidative phosphorylation eradicates therapy-resistant chronic myeloid leukemia stem cells. Nat Med.

[15] Amaya ML (2022). The STAT3-MYC axis promotes survival of leukemia stem cells by regulating SLC1A5 and oxidative phosphorylation. Blood.

[16] Sessions DT, Kashatus DF (2021). Mitochondrial dynamics in cancer stem cells. Cell Mol Life Sci.

[17] Naik PP (2019). Mitophagy-driven metabolic switch reprograms stem cell fate. Cell Mol Life Sci.

[18] Sancho P (2015). MYC/PGC-1α balance determines the metabolic phenotype and plasticity of pancreatic cancer stem cells. Cell Metab.

[19] Valle S (2020). Exploiting oxidative phosphorylation to promote the stem and immunoevasive properties of pancreatic cancer stem cells. Nat Commun.

[20] Praharaj PP (2021). Mitochondrial rewiring through mitophagy and mitochondrial biogenesis in cancer stem cells: A potential target for anti-CSC cancer therapy. Cancer Lett.

[21] Alcalá S (2020). ISG15 and ISGylation is required for pancreatic cancer stem cell mitophagy and metabolic plasticity. Nat Commun.

[22] Nazio F (2019). Autophagy and cancer stem cells: molecular mechanisms and therapeutic applications. Cell Death Differ.

[23] Feng X (2021). Mitophagy promotes the stemness of bone marrow-derived mesenchymal stem cells. Exp Biol Med (Maywood).

[24] Yang M (2015). Blockade of autophagy reduces pancreatic cancer stem cell activity and potentiates the tumoricidal effect of gemcitabine. Mol Cancer.

[25] Xiao YY (2022). Metformin-induced AMPK activation promotes cisplatin resistance through PINK1/Parkin dependent mitophagy in gastric cancer. Front Oncol.

[26] Wang X (2022). Gamma-glutamyltransferase 7 suppresses gastric cancer by cooperating with RAB7 to induce mitophagy. Oncogene.

[27] Wang Q (2021). RNA binding protein DAZAP1 promotes HCC progression and regulates ferroptosis by interacting with SLC7A11 mRNA. Exp Cell Res.

[28] Chen Y (2020). Starvation-induced suppression of DAZAP1 by miR-10b integrates splicing control into TSC2-regulated oncogenic autophagy in esophageal squamous cell carcinoma. Theranostics.

[29] Yu M (2019). Genome-wide profiling of prognostic alternative splicing pattern in pancreatic cancer. Front Oncol.

[30] Choudhury R (2014). The splicing activator DAZAP1 integrates splicing control into MEK/Erk-regulated cell proliferation and migration. Nat Commun.

[31] Nadeu F (2020). Genomic and epigenomic insights into the origin, pathogenesis, and clinical behavior of mantle cell lymphoma subtypes. Blood.

[32] Deng J (2022). DAZAP1 overexpression promotes growth of HCC cell lines: a primary study using CEUS. Clin Transl Oncol.

[33] Zhang J (2024). DAZAP1 phase separation regulates mitochondrial metabolism to facilitate invasion and metastasis of oral squamous cell carcinoma. Cancer Res.

[34] Song C (2022). Mitophagy: a novel perspective for insighting into cancer and cancer treatment. Cell Prolif.

[35] Novak I (2012). Mitophagy: a complex mechanism of mitochondrial removal. Antioxid Redox Signal.

[36] Liu L (2023). Crosstalk between mitochondrial biogenesis and mitophagy to maintain mitochondrial homeostasis. J Biomed Sci.

[37] Lin MG, Hurley JH (2016). Structure and function of the ULK1 complex in autophagy. Curr Opin Cell Biol.

[38] Hung CM (2021). AMPK/ULK1-mediated phosphorylation of Parkin ACT domain mediates an early step in mitophagy. Sci Adv.

[39] Palikaras K (2018). Mechanisms of mitophagy in cellular homeostasis, physiology and pathology. Nat Cell Biol.

[40] Maquat LE (2004). Nonsense-mediated mRNA decay: splicing, translation and mRNP dynamics. Nat Rev Mol Cell Biol.

[41] García-Moreno JF, Romão L (2020). Perspective in alternative splicing coupled to nonsense-mediated mRNA decay. Int J Mol Sci.

[42] Temaj G (2024). Advances in molecular function of UPF1 in Cancer. Arch Biochem Biophys.

[43] Zhou Y (2022). DAZAP1 facilitates the alternative splicing of KITLG to promote multiple myeloma cell proliferation via ERK signaling pathway. Aging (Albany NY).

[44] Goina E (2008). Binding of DAZAP1 and hnRNPA1/A2 to an exonic splicing silencer in a natural BRCA1 exon 18 mutant. Mol Cell Biol.

[45] Choudhury R (2014). The splicing activator DAZAP1 integrates splicing control into MEK/Erk -regulated cell proliferation and migration. Nat Commun.

[46] Goina E (2008). Binding of DAZAP1 and hnRNPA1/A2 to an exonic splicing silencer in a natural BRCA1 exon 18 mutant. Mol Cell Biol.

[47] Lee KM (2017). MYC and MCL1 cooperatively promote chemotherapy-resistant breast cancer stem cells via regulation of mitochondrial oxidative phosphorylation. Cell Metab.

[48] Deng J (2023). Exosomal transfer leads to chemoresistance through oxidative phosphorylation-mediated stemness phenotype in colorectal cancer. Theranostics.

[49] Li Y (2023). PINK1-mediated mitophagy promotes oxidative phosphorylation and redox homeostasis to induce drug-tolerant persister cancer cells. Cancer Res.

[50] Pei S (2018). AMPK/FIS1-mediated mitophagy is required for self-renewal of human AML stem cells. Cell Stem Cell.

[51] Luo J (2024). Enhanced mitophagy driven by ADAR1-GLI1 editing supports the self-renewal of cancer stem cells in HCC. Hepatology.

[52] Karimi P (2014). Gastric cancer: descriptive epidemiology, risk factors, screening, and prevention. Cancer Epidemiol Biomarkers Prev.

[53] Chandanos E, Lagergren J (2008). Oestrogen and the enigmatic male predominance of gastric cancer. Eur J Cancer.

[54] Bagheri V (2018). Isolation and identification of chemotherapy-enriched sphere-forming cells from a patient with gastric cancer. J Cell Physiol.

[55] Jiang Z (2016). The effects and mechanisms of SLC34A2 on tumorigenicity in human non-small cell lung cancer stem cells. Tumour Biol.

[56] Chen D (2024). Sangerbox 2: enhanced functionalities and update for a comprehensive clinical bioinformatics data analysis platform. Imeta.

