# Supplementary material for: DAZAP1 maintains gastric cancer stemness by inducing mitophagy
Source: JCI Insight. 2025 May 22;10(10):e175422. doi: 10.1172/jci.insight.175422 (PMC12128983; doi:10.1172/jci.insight.175422)
Supplement: undefined [file jciinsight-10-175422-s080.pdf]

SUPPLEMENTARY METHODS

1. **Western Blotting:** Tumor cells were collected and lysed on ice for 15 minutes using RIPA lysis buffer. Cellular proteins were quantified using the Bradford method, separated by 10% or 15% SDS-PAGE, and transferred to a nitrocellulose membrane. After blocking with 5% skim milk, membranes were incubated with diluted primary antibodies overnight at 4 °C, according to the manufacturer's instructions. Membranes were then washed with 1x TBST and incubated with secondary antibodies at room temperature for 2 hours. Protein bands were visualized using a chemiluminescence assay and captured for quantitative analysis. Details regarding all antibodies and their working concentrations are provided in Supplementary Table 2.
2. **Real-Time Quantitative PCR**: Tumor cells in 6-cm dishes were collected, lysed with 1 ml TRIzol, and incubated at room temperature for 5 minutes. Chloroform (200 μl) was added, shaken vigorously, and incubated for 10 minutes. Total RNA was extracted following precipitation with isopropanol and 70% ethanol. cDNA was synthesized using a reverse transcription kit and quantified using real-time quantitative PCR with the SYBR Green master mix. All primer sequences used are listed in Supplementary Table 1.
3. **CCK8 Assay**: Approximately 2×10^3 gastric cancer (GC) cells per well were seeded in 96-well plates and cultured for 4 days. Each day, 10 μl of CCK8 working buffer was added to 100 μl of medium and incubated at 37 °C for 2 hours. Absorbance was measured at 450 nm using a multifunctional microplate reader (Biotek Cytation5, USA). All experiments were performed in triplicate.
4. **Wound Healing Assay**: GC cells were grown in a 24-well plate to a confluency of over 90%, and the cell surface was scratched using a 200 μl pipette tip. After washing with PBS, DMEM medium without FBS was used. The scratch width was measured under a

microscope, and cells were incubated at 37 °C for 24 hours before photographing again. The scratch area was compared to evaluate cell motility.

5. **Transwell Assay**: The invasive and migratory abilities of the cells were examined using Transwell chambers. For migration assays, 0.5×10^5 GC cells were resuspended in 200 μl of serum-free medium and placed in the upper chamber, while 600 μl of medium containing 10% fetal bovine serum was added to the lower chamber. For invasion assays, the upper chamber was coated with Matrigel (BD, USA) and incubated for 1 hour at 37 °C. GC cells (2×10^5) were added to the upper chamber, and 600 μl of complete medium was added to the lower chamber and incubated at 37 °C for 24-48 hours. The cells were fixed with 4% paraformaldehyde, stained with 0.1% crystal violet, and counted for analysis
6. **Extreme Limiting Dilution Assay (ELDA)**: GC cells were resuspended in complete DMEM/F12 medium and seeded at varying densities (50, 20, 10, 5, and 1 cell per well) into 96-well ultra-low attachment plates in 20 replicates. After 14 days, the number of spheres was counted and analyzed using the ELDA website (http://bioinf.wehi.edu.au/software/elda/).
7. **Mass Spectrometry**: Tumor cells grown in 10-cm dishes were collected into centrifuge tubes after washing with PBS. The cells were lysed and processed for mass spectrometry according to the manufacturer's instructions (BestBio, China). A label-free proteomic analysis was performed using the Orbitrap high-resolution mass spectrometer (Thermo Fisher, USA). Differentially expressed proteins were subjected to KEGG functional enrichment analysis based on the latest KEGG annotated gene set.
8. **Immunohistochemistry**: Paraffin-embedded tissue sections (approximately 5 μm thick) were dewaxed, hydrated, antigen-retrieved, and blocked. DAZAP1 and ULK1 antibodies (1:100) were applied and incubated overnight at 4 °C, followed by secondary antibody

incubation at room temperature for 1 hour. DAB and hematoxylin staining were then performed. The stained sections were analyzed using QuPath software to calculate the H-score, which quantifies the proportion and intensity of positively stained cells, ranging from 0 to 300 (1). Spearman's correlation analysis was performed to evaluate the relationship between gene expression levels based on these scores. Samples were categorized into high and low expression groups based on the mean H-score of protein.

9. **Immunofluorescence**: To observe Parkin localization on the mitochondrial surface, mitochondria were labeled using the MTDR probe. Cells were washed with PBS, fixed with 4% paraformaldehyde for 10 minutes, and permeabilized with 0.5% Triton X-100 for 10 minutes at room temperature. Cells were blocked with 3% BSA for 1 hour and incubated overnight at 4 °C with a diluted Parkin antibody (1:200). After washing, an Alexa Fluor 488-labeled secondary antibody was added and incubated for 2 hours at room temperature. Samples were mounted with a fluorescence medium, and Parkin localization was observed under a fluorescence microscope.
10. **scRNA-seq Data Analysis**: We downloaded four scRNA-seq data of gastric cancer, including GSE134520 (2), GSE158631 (3), GSE163558 (4), and GSE167297 (5) for further analysis. The "Seurat" package was used to conduct quality control, filtering, normalization, and scaling on the raw data (6). The "Harmony" package was then applied for data integration and dimensionality reduction, with visualization performed using PCA or t-SNE algorithms (7). Cell clusters were annotated into three major categories based on specific markers: epithelial/cancer (EPCAM, KRT19, PROM1, ALDH1A1, CD24), immune (CD45+, PTPRC), and stromal (MME, PECAM1). The "ggpubr" package was utilized for gene expression analysis and visualization. The "monocle" package was used to analyze the transcriptional trajectories of cells, facilitating the exploration of DAZAP1's dynamic changes during gastric cancer

progression (8-10). The CytoTRACE algorithm, developed by Gulati et al., was used to score individual cells based on a specific gene set, reflecting their level of stemness (11). CytoTRACE scores range from 0 to 1, with higher scores indicating higher stemness (less differentiation), and vice versa. The gene set 'c2.all.v2023.1.Hs.symbols.gmt' was obtained from the MSigDB website (http://www.gsea-msigdb.org/gsea/msigdb). Gene Set Enrichment Analysis (GSEA) was performed on the DAZAP1 gene using the "GSEABase" package in scRNA-seq data to identify enriched signaling pathways.

11. **Bioinformatics Analysis:** We analyzed the differential expression of DAZAP1 in acquired drug-resistant tissues using the GSE14210 dataset and compared it to expression levels before chemotherapy treatment. The Kaplan-Meier Plotter online platform was used to examine the correlation between DAZAP1 expression and survival status in gastric cancer (GC) patients treated with 5-FU. Based on the tumor stemness index derived from the OCLR algorithm developed by Malta et al., we integrated the stemness index with the gene expression data of the samples for differential expression analysis (12). For Gene Set Enrichment Analysis (GSEA), samples were divided into two groups based on the median DAZAP1 value, and GSEA (version 3.0) and Perl software were used for functional enrichment analysis. A P-value of < 0.05 and an FDR of < 0.25 were considered statistically significant.

SUPPLEMENTARY FIGURES

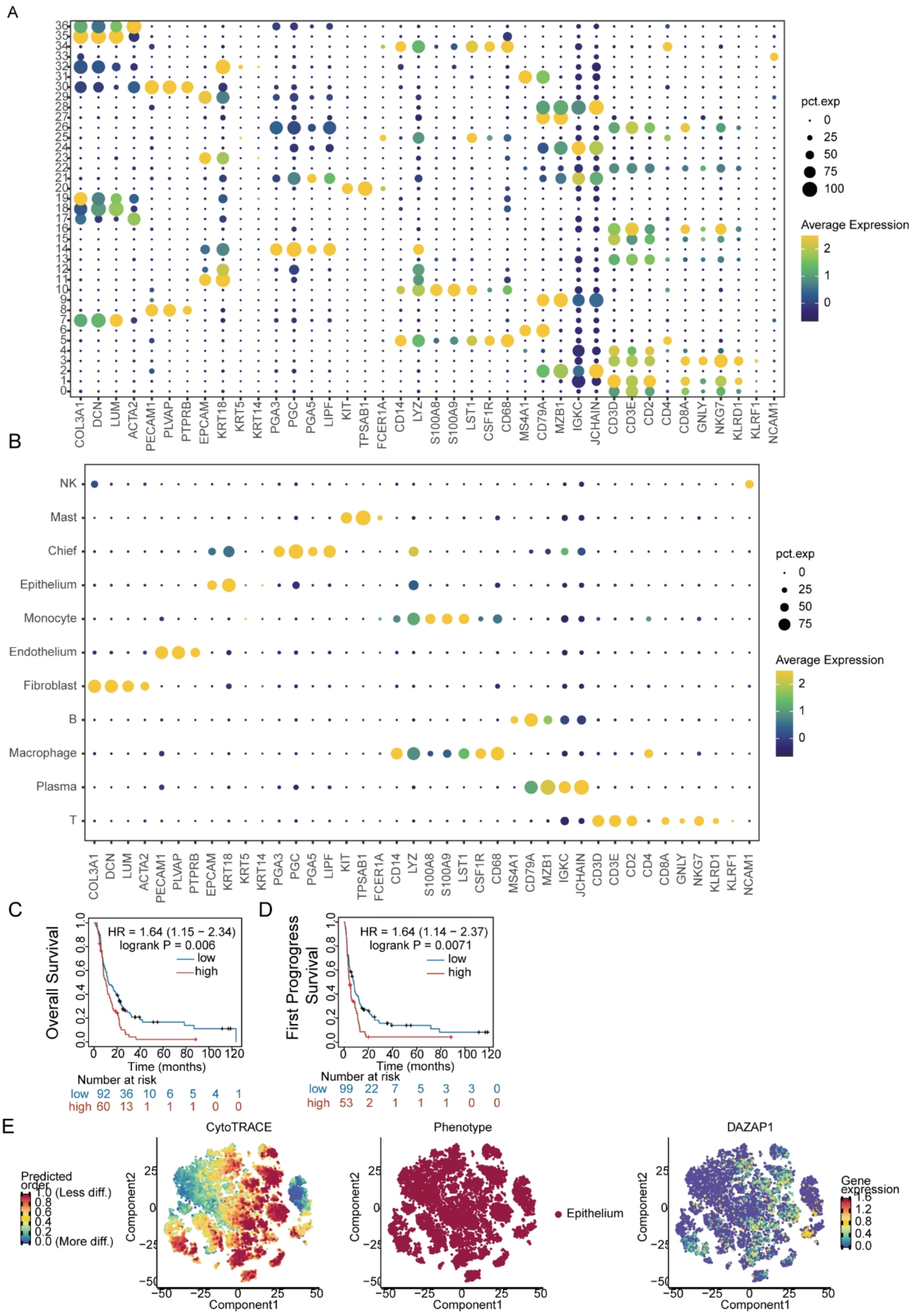

Figure S1. DAZAP1 Expression is Upregulated in GCSCs. (Related to Figure 1)

(A) KNN algorithm categorizing cells into 37 clusters, annotated into 11 major cell types based on marker gene expression, including epithelial cells (non-malignant and cancer cells), fibroblasts, endothelial cells, chief cells, T lymphocytes, B lymphocytes, plasma cells, NK

cells, monocytes, macrophages, and mast cells. (B) Heatmap of marker gene expression for major cell types identified in gastric cancer samples. (C-D) Kaplan-Meier survival curves from the GEO database (GSE14210 and GSE15459 cohorts) showing that GC patients with elevated DAZAP1 expression have worse overall survival (P=0.006) and progression-free survival (P=0.007) compared to the low DAZAP1 expression group. (E) CytoTRACE analysis predicting the differentiation potential of single cells based on their differentiation state, showing the regulatory activity in gastric cancer evolution. *P < 0.05; **P < 0.01; ***P < 0.001.

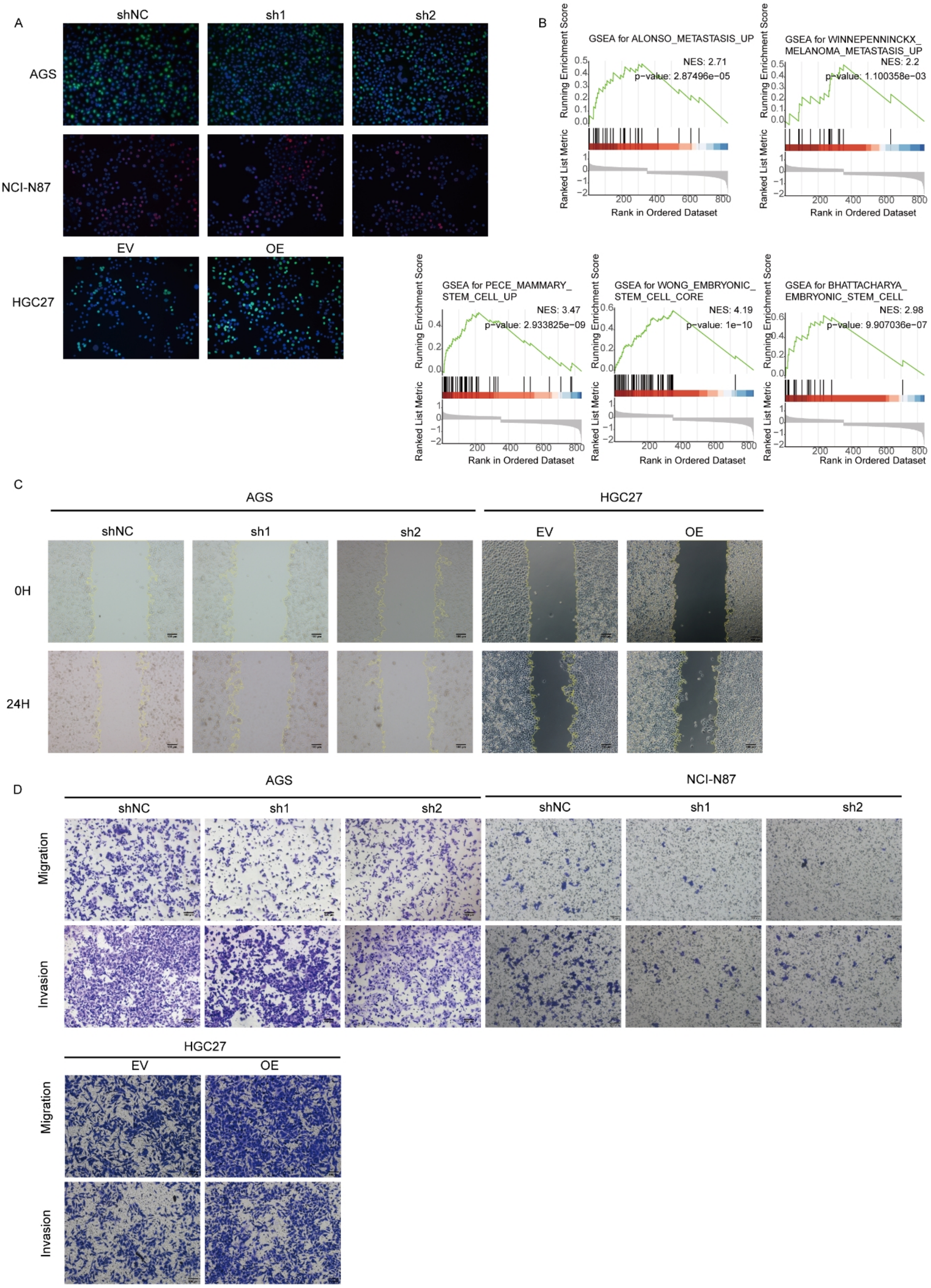

Figure S2. DAZAP1 Regulates Cancer Cell Proliferation and Metastasis. (Related to Figure 3)

(A) EdU assays showing that DAZAP1 knockdown inhibits cell proliferation, while DAZAP1 overexpression significantly enhances it. (B) Gene Set Enrichment Analysis (GSEA) of scRNA-seq data revealing that DAZAP1 is enriched in metastatic and stemness-related pathways, including ALONSO_METASTASIS_UP, WINNEPENNINCKX_MELANOMA_METASTASIS_UP, PECE_MAMMARY_STEM_CELL_UP, WONG_EMBRYONIC_STEM_CELL_CORE, and BHATTACHARYA_EMBRYONIC_STEM_CELL. (C) Microscopic images of the wound healing assay (0/24 hours, scale bar: 100 μm). (D) Representative images of Transwell migration and invasion assays (24 h; scale bar: 100 μm).

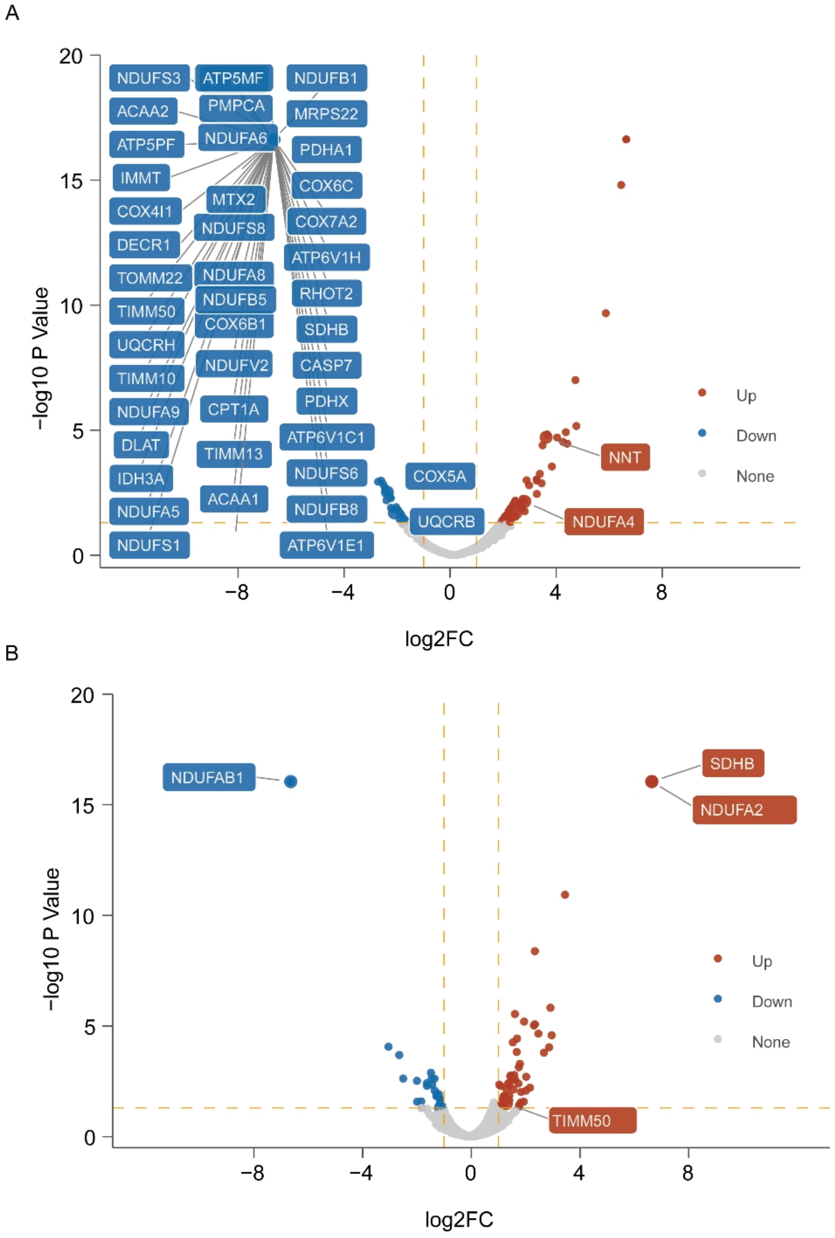

Figure S3. DAZAP1 modulates OXPHOS activity. (Related to Figure 5)
(A) Downregulation of OXPHOS-related genes in DAZAP1-knockdown cells. (B) Upregulated expression of SDHB, TIMM50, and NDUFA2 in DAZAP1-overexpressing cells.

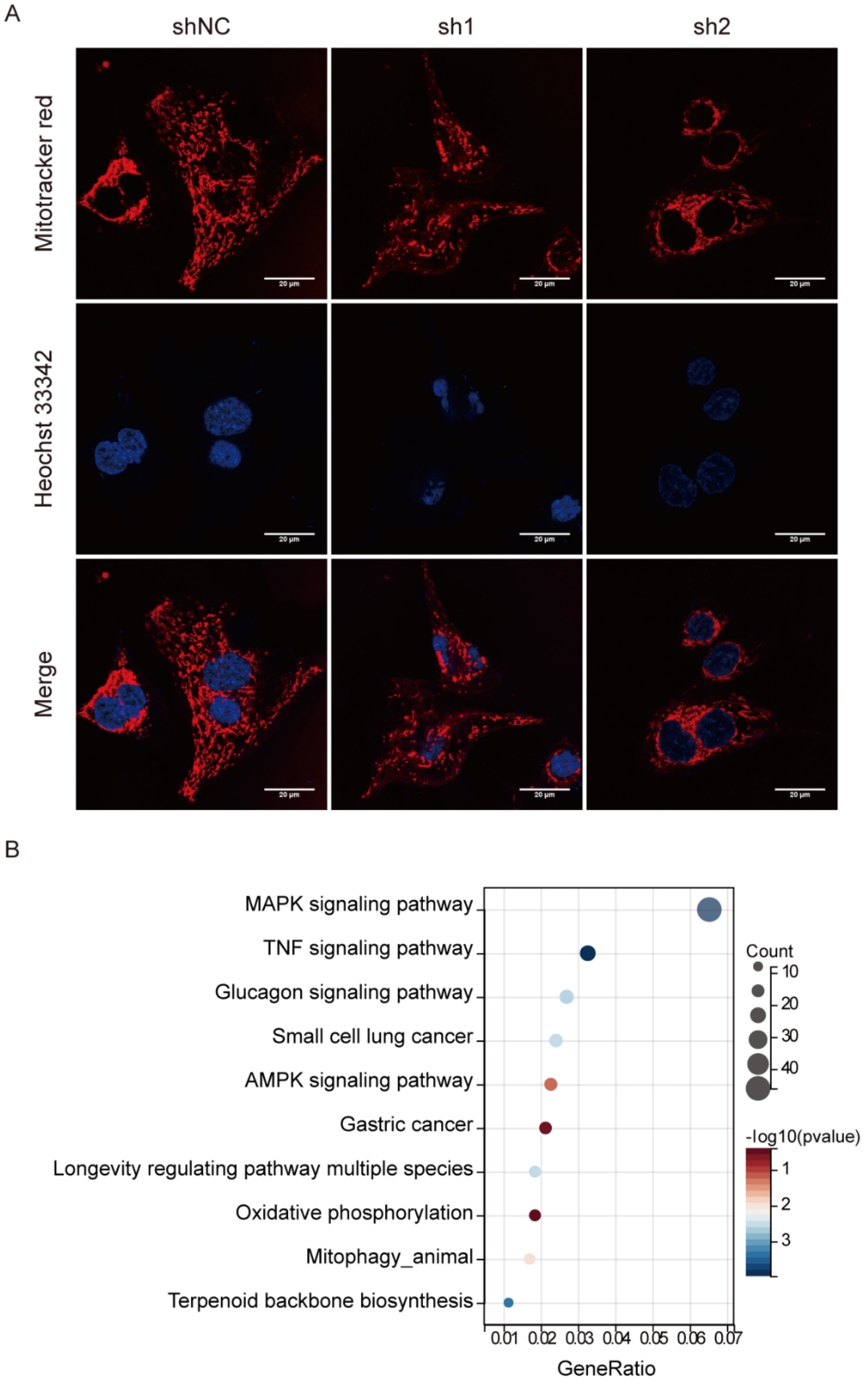

Figure S4. DAZAP1 is Involved in Mitophagy Regulation. (Related to Figure 6)
(A) Representative images of mitochondria labeled with the MitoTracker probe (scale bar: 20 μm). (B) RNA sequencing analysis suggests that mitophagy is a downstream pathway of DAZAP1.

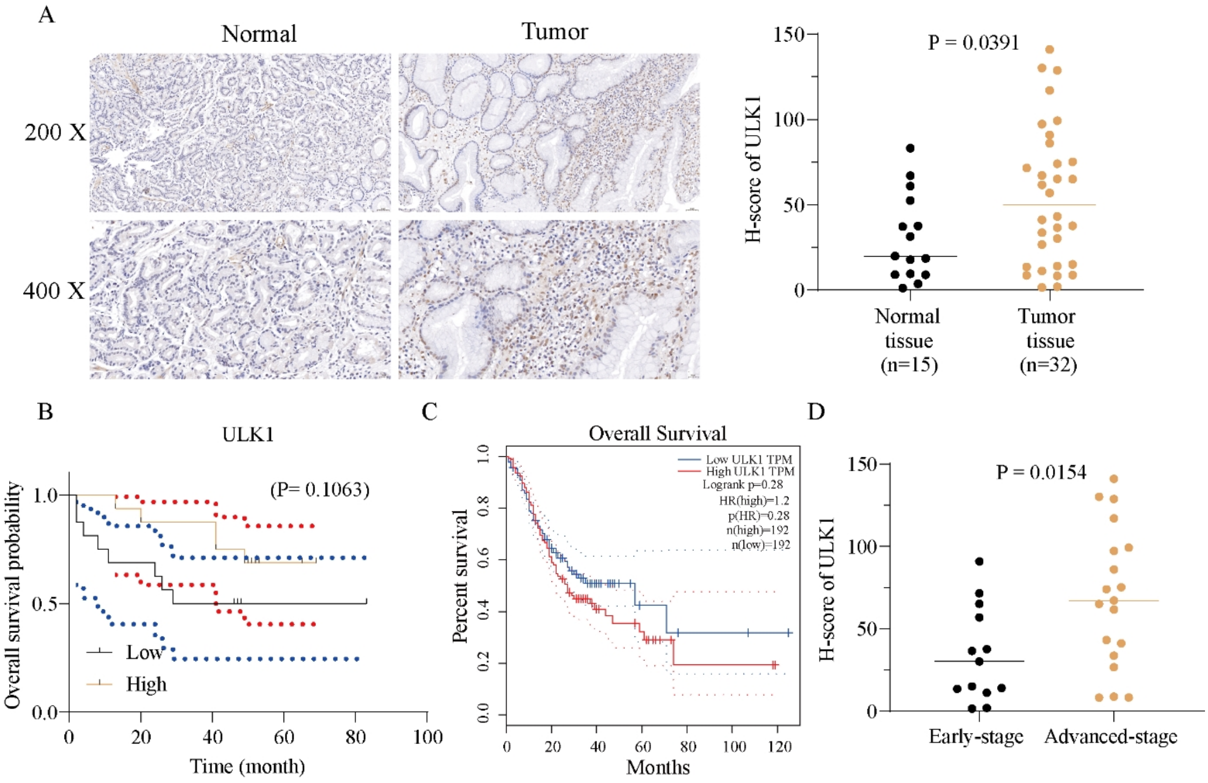

Figure S5. ULK1 Expression in Gastric Cancer Tissues and Its Clinical Relevance. (A) ULK1 is significantly upregulated in gastric cancer tissues compared to normal tissues (P = 0.0391). (B-C) Kaplan-Meier analysis shows no significant association between ULK1 expression and overall survival in our gastric cancer cohort (B) and the TCGA-STAD cohort (C). (D) Analysis of the TCGA-STAD dataset reveals that ULK1 expression is significantly higher in advanced-stage (Stage III+IV) gastric cancer patients (P = 0.0154). Statistical analysis in (A and D) by unpaired Student's t test, and (B and C) by Log-rank (Mantel-Cox) test. *P < 0.05, **P < 0.01, and ***P < 0.001.

Supplementary Table 1. Pairwise Analysis of Stem Cell Frequency Differences.

Group	CSC frequency	P.value (vs ctrl)
AGS-shNC	1/83.7	
AGS-sh1	1/376.2	0.00000156
AGS-sh2	1/218	0.00139
HGC27-EV	1/18.61	
HGC27-OE	1/9.46	0.0143

Supplementary Table 2. Analysis of Stem Cell Frequency Differences In Vivo

Supplementary Table 2.1

Counter	Dose	Tested	Response	Group
1	100000	20	12	NCI-N87-shNC
2	50000	20	4	NCI-N87-shNC
3	10000	20	1	NCI-N87-shNC
4	100000	20	3	NCI-N87-sh1
5	50000	20	1	NCI-N87-sh1
6	10000	30	1	NCI-N87-sh1

Supplementary Table 2.2

Group	1/(stem cell frequency)			P.value (vs ctrl)
	Lower	Estimate	Upper	
NCI-N87-shNC	229221	142318	88362	
NCI-N87-sh1	1500007	623128	258858	0.00141

Supplementary Table 3. Reagents and kits used in this study.

Kit	Company (Cat. No.)
CCK8	GLPBIO (GK10001)
DAB	BOSTER (AR1022)
Recombinant Human FGF-basi	PeproTech (100-18B)
Recombinant Human EGF	PeproTech (100-47)
50X B27	Gibco (A1486701)
Insulin	Beyotime (P3376-100IU)
Gboxin	MCE (HY-111651)
Mdivi-1	MCE (HY-15886)
ALDEFLUOR Stem Cell Identification Kit	Stemcell (01700)
Seahorse XF Cell Mito Stress Test Ki	Agilent (103015-100)
JC-1 Kit	Beyotime (C2006)
MitoTracker Deep Red	ThermoFisher (M22426)
MitoTracker Red CMXRos	ThermoFisher (M7512)
LysoSensor Green DND-189	ThermoFisher (L7535)
RIP Kit	BersinBio (Bes5101)

Supplementary Table 4. Sequences of primers used in qRT-PCR and PCR analysis

Gene	Forward Primer(5'-3')	Reverse Primer(5'-3')
DAZAP1	AGGACCCAGGAGCGATAACA	CTCCGTGACCACTCCGAAC
NANOG	AAGGTCCCGGTCAAGAAACAG	CTTCTGCGTCACACCATTGC
SOX2	TACAGCATGTCCTACTCGCAG	GAGGAAGAGGTAACCACAGGG
OCT4	CTGGGTTGATCCTCGGACCT	CCATCGGAGTTGCTCTCCA
UQCRC1	GGGGCACAAGTGCTATTGC	GTTGTCCAGCAGGCTAACC
UQCRC2	TTCAGCAATTTAGGAACCACCC	GGTCACACTTAATTTGCCACCA A
SDHA	TGGCATTTCTACGACACCGTG	GCCTGCTCCGTCATGTAGTG
SDHB	GACACCAACCTCAATAAGGTCT C	GGCTCAATGGATTTGTACTGTG C
ATP5A1	ATGACGACTTATCCAAACAGGC	CGGGAGTGTAGGTAGAACACAT
ATP5F1 B	AAACAATTTGCTCCCATTCATGC	GACAACCTTGATACCAGTCACC
NDUFA1	GCGTACATCCACAGGTTCACT	GCGCCTATCTCTTTCCATCAGA
β-actin	CATGTACGTTGCTATCCAGGC	CTCCTTAATGTCACGCACGAT
ULK1	GGCAAGTTCGAGTTCTCCCG	CGACCTCCAAATCGTGCTTCT
ULK1-primer-1	ACCCAGTTCCAAACACCTCG	GCAGAGGAGCCTGGACGA
ULK1-primer-2	CTTCCACGGCTCCCATAGAG	ACCTTGAGGAGATGGCGTGT

Supplementary Table 5. Primary antibodies used in this study.

Antibody	Company (Cat. No.)	Working Concentration Dilutions
DAZAP1	Zenbio(R24078)	WB: 1/2000; IHC:1/500; IP:5ug; IF:1/200
ULK1	Zenbio(381887)	WB: 1/1000
OCT4	Abclonal(A25315)	WB: 1/1000
SOX2	Abclonal(A11501)	WB: 1/1000
NANOG	Abclonal(A22625)	WB: 1/1000
hnRNPA1	Abclonal(A11564)	WB: 1/1000
P62	Zenbio(R25788)	WB: 1/1000
LC3B	Zenbio(350140)	WB: 1/300
β-actin	Proteintech (20536-1-AP)	WB: 1/3000
a-Tubulin	Proteintech (11224-1-AP)	WB: 1/3000
IgG control	Proteintech (30000-0-AP)	IP:5ug

REFERENCE

1. Bankhead P, et al. QuPath: Open source software for digital pathology image analysis. Scientific Reports. 2017;7(1):16878.
2. Zhang P, et al. Dissecting the Single-Cell Transcriptome Network Underlying Gastric Premalignant Lesions and Early Gastric Cancer. Cell reports. 2019;27(6):1934-47.e5.
3. Wang B, et al. Comprehensive analysis of metastatic gastric cancer tumour cells using single-cell RNA-seq. Scientific reports. 2021;11(1):1141.
4. Jiang H, et al. Revealing the transcriptional heterogeneity of organ-specific metastasis in human gastric cancer using single-cell RNA Sequencing. Clinical and translational medicine. 2022;12(2):e730.
5. Jeong HY, et al. Spatially Distinct Reprogramming of the Tumor Microenvironment Based On Tumor Invasion in Diffuse-Type Gastric Cancers. Clinical cancer research : an official journal of the American Association for Cancer Research. 2021;27(23):6529-42.
6. Hao Y, et al. Integrated analysis of multimodal single-cell data. Cell. 2021;184(13):3573-87.e29.
7. Korsunsky I, et al. Fast, sensitive and accurate integration of single-cell data with Harmony. Nature methods. 2019;16(12):1289-96.
8. Trapnell C, et al. The dynamics and regulators of cell fate decisions are revealed by pseudotemporal ordering of single cells. Nature biotechnology. 2014;32(4):381-6.
9. Qiu X, et al. Single-cell mRNA quantification and differential analysis with Census. Nature methods. 2017;14(3):309-15.
10. Qiu X, et al. Reversed graph embedding resolves complex single-cell trajectories. Nature methods. 2017;14(10):979-82.
11. Gulati GS, et al. Single-cell transcriptional diversity is a hallmark of developmental potential. Science (New York, NY). 2020;367(6476):405-11.
12. Malta TM, et al. Machine Learning Identifies Stemness Features Associated with Oncogenic Dedifferentiation. Cell. 2018;173(2):338-54.e15.
