# Supplementary material for: DAZAP1 maintains gastric cancer stemness by inducing mitophagy
Source: JCI Insight. 2025 May 22;10(10):e175422. doi: 10.1172/jci.insight.175422 (PMC12128983; doi:10.1172/jci.insight.175422)

Full unedited gel for Figure 1G

DAZAP1

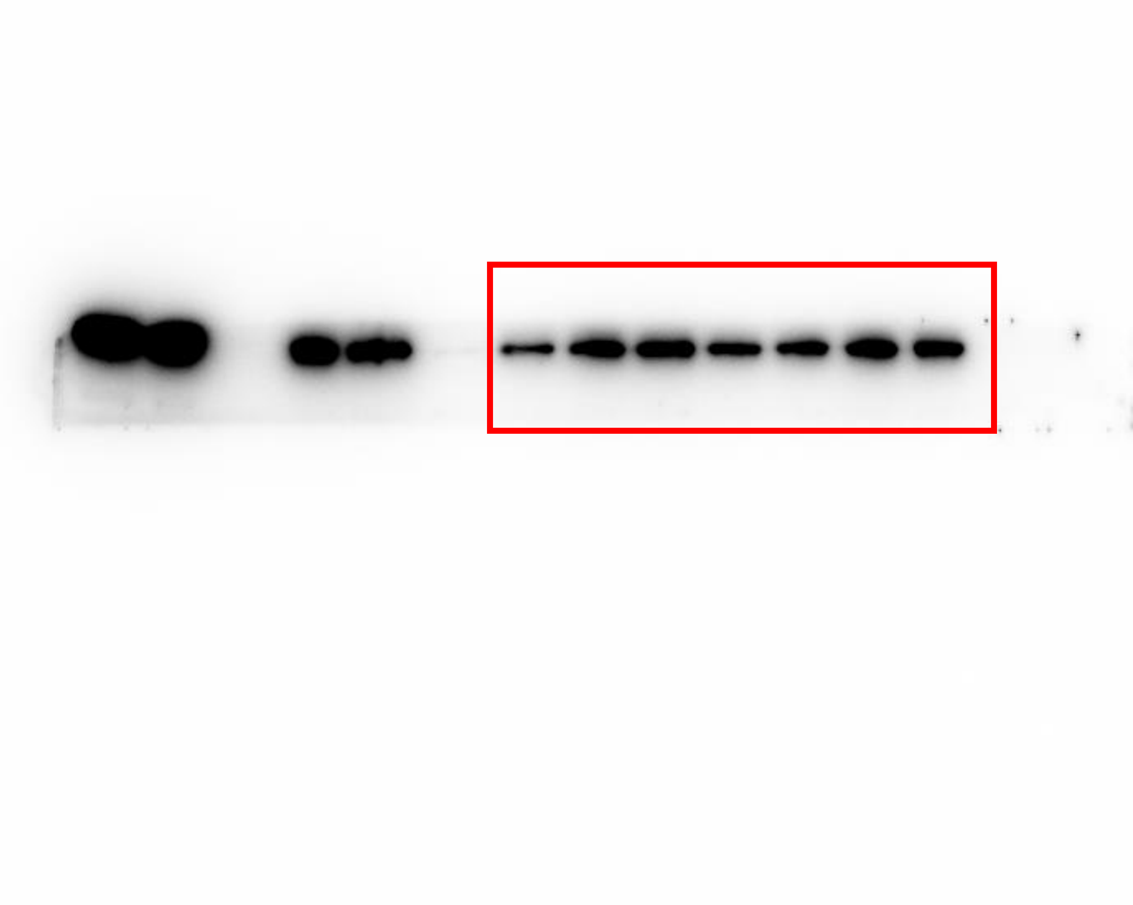

a-Tubulin

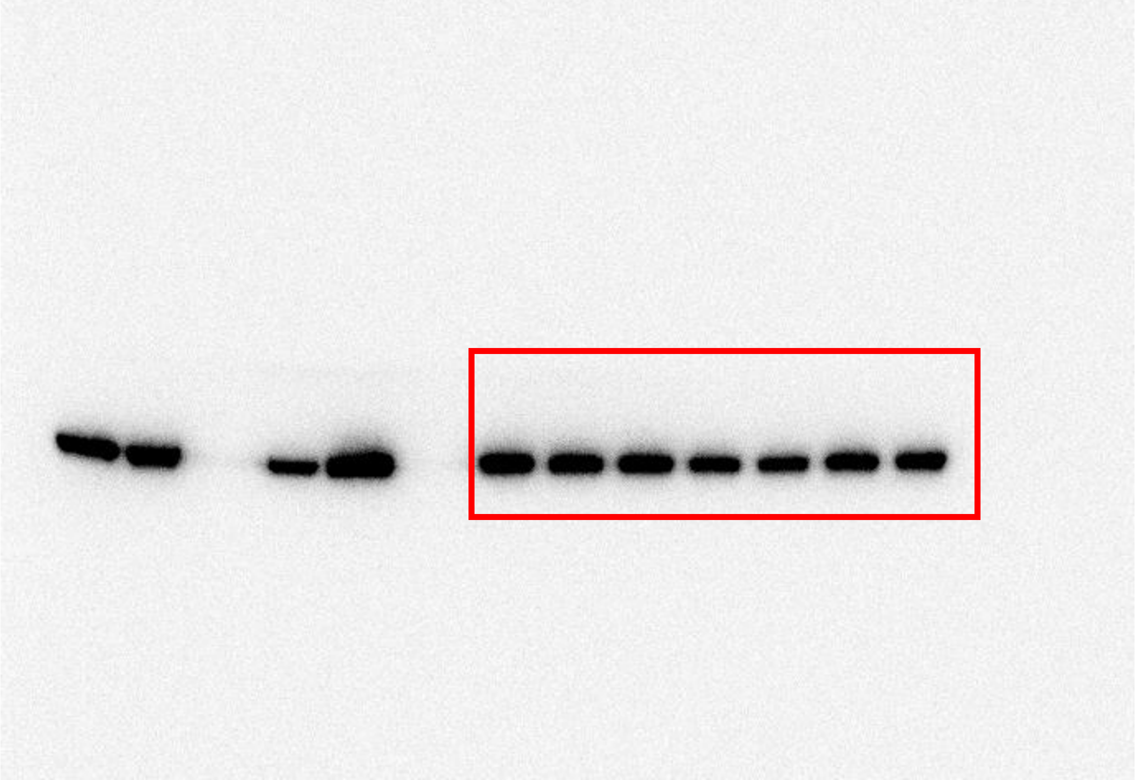

Full unedited gel for Figure 2H

DAZAP1

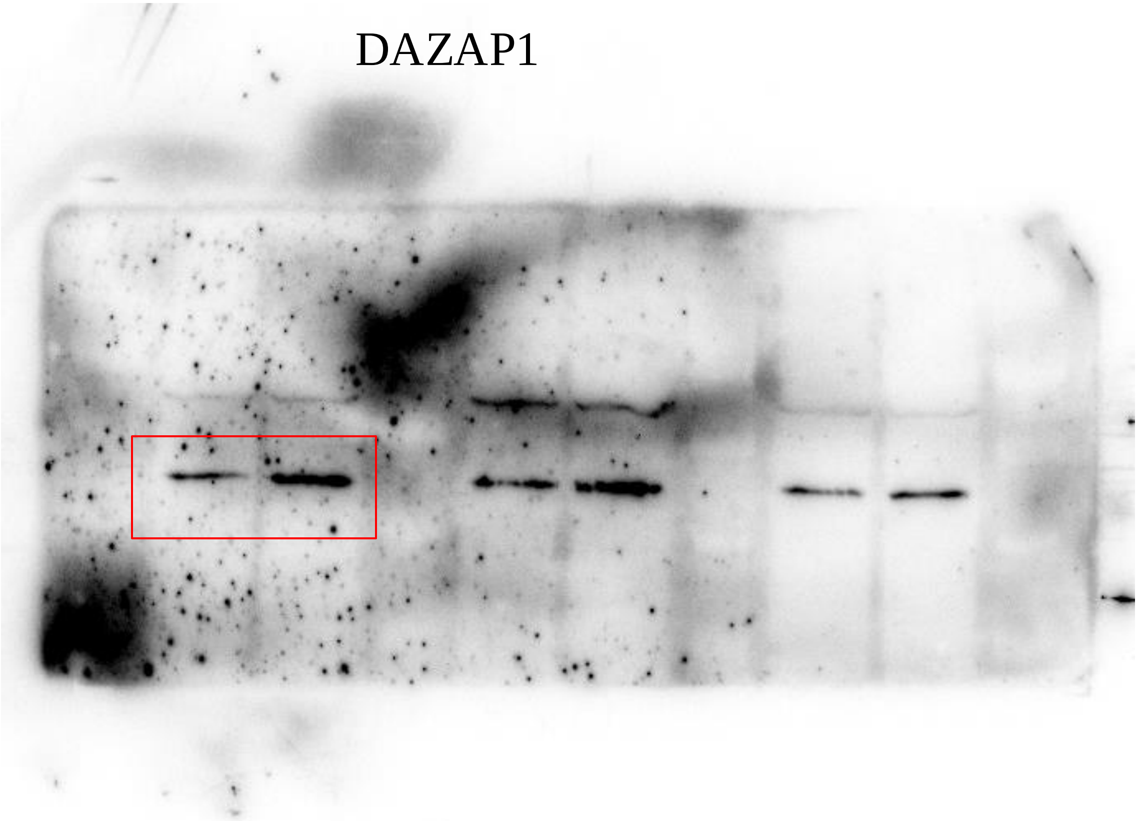

a-Tubulin

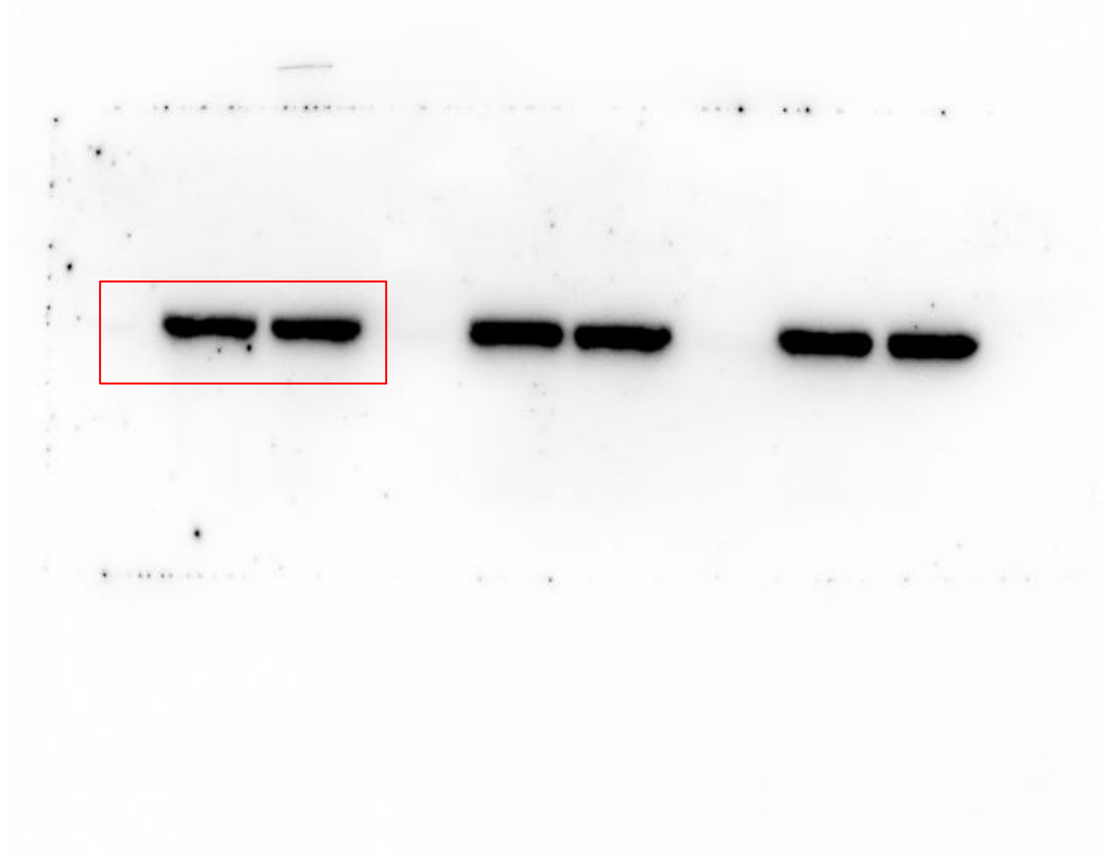

Full unedited gel for Figure 2H

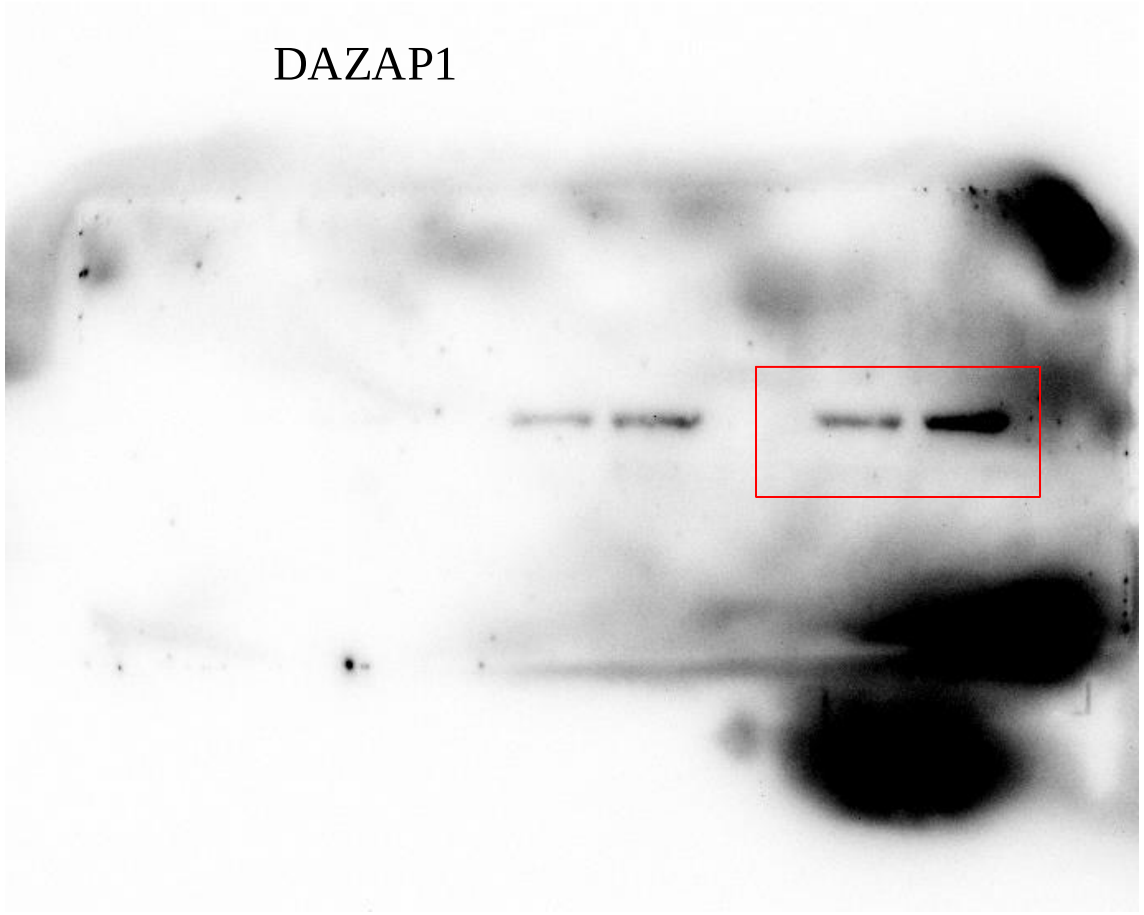

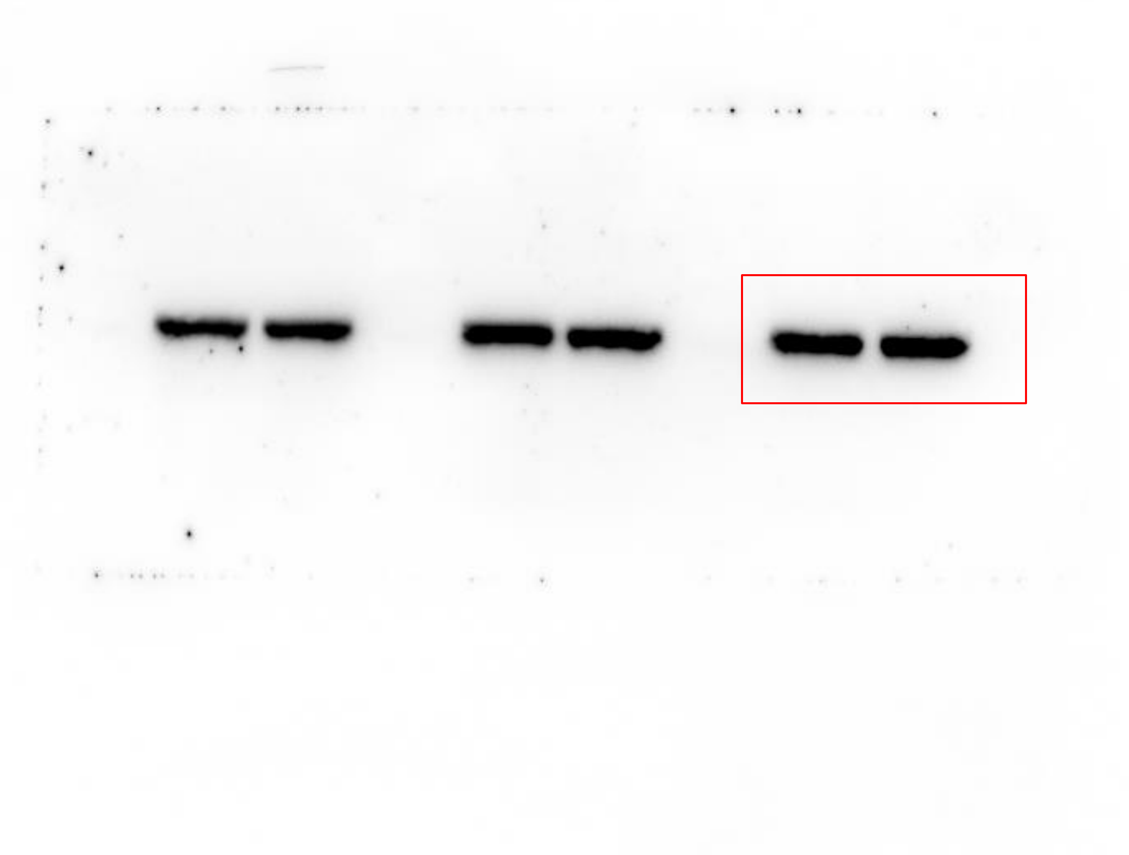

Full unedited gel for Figure 2H

DAZAP1

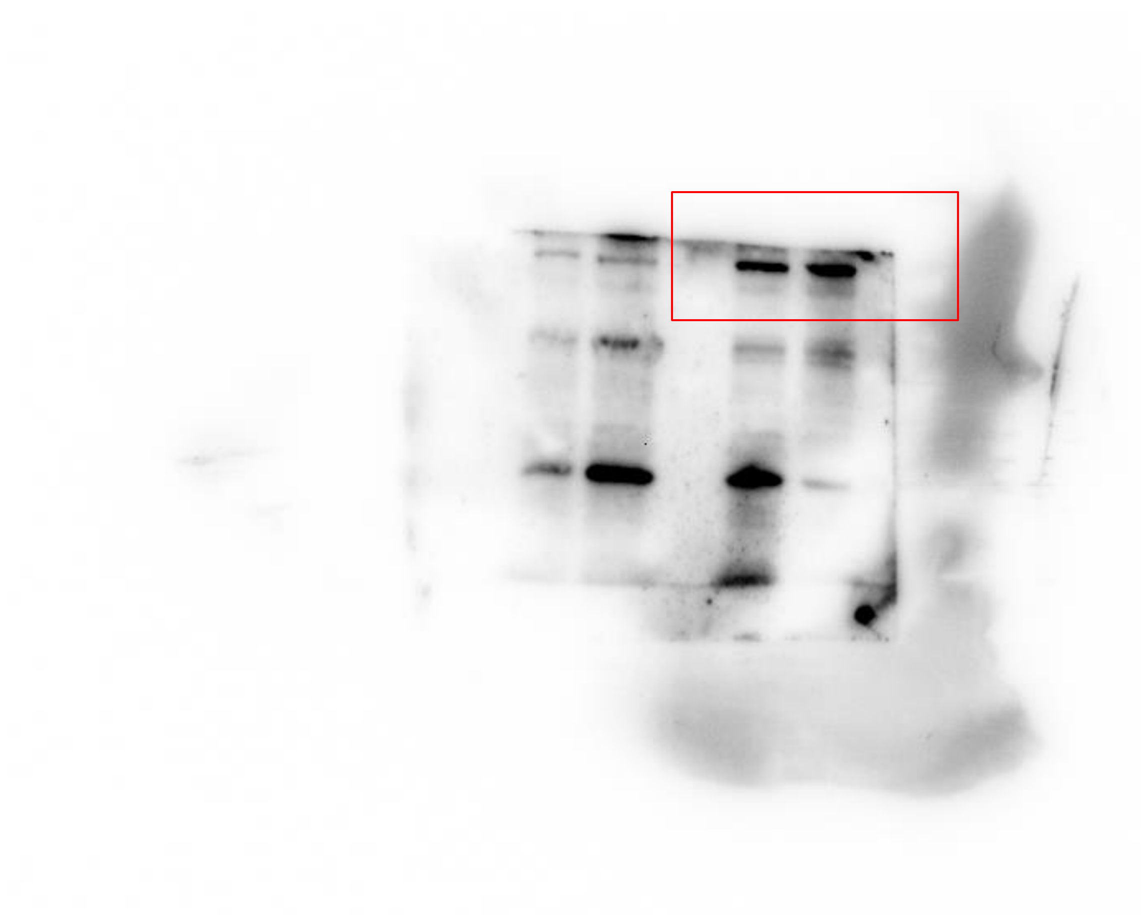

a-Tubulin

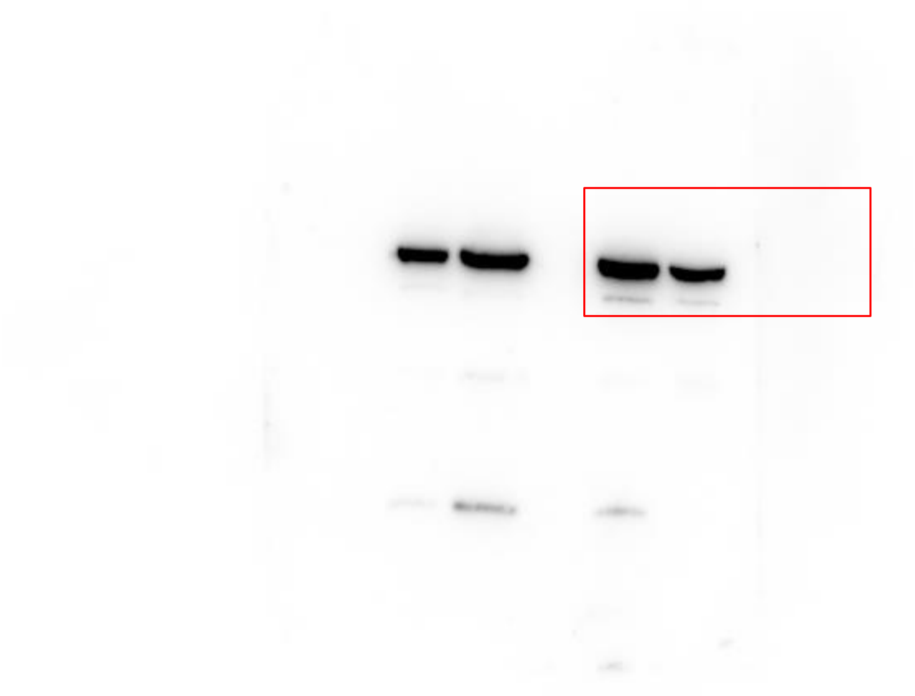

Full unedited gel for Figure 2H

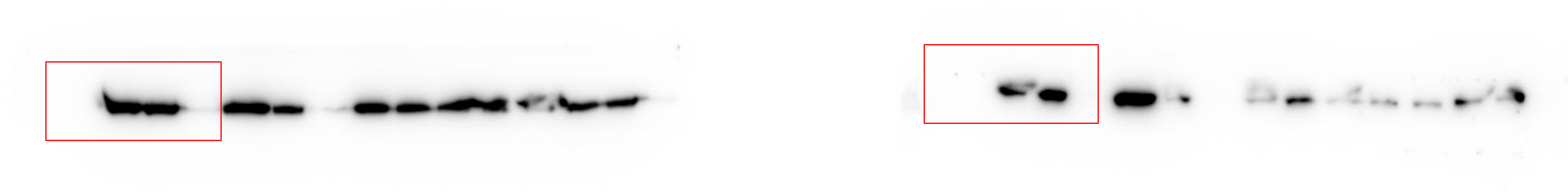

Full unedited gel for Figure 2H

DAZAP1

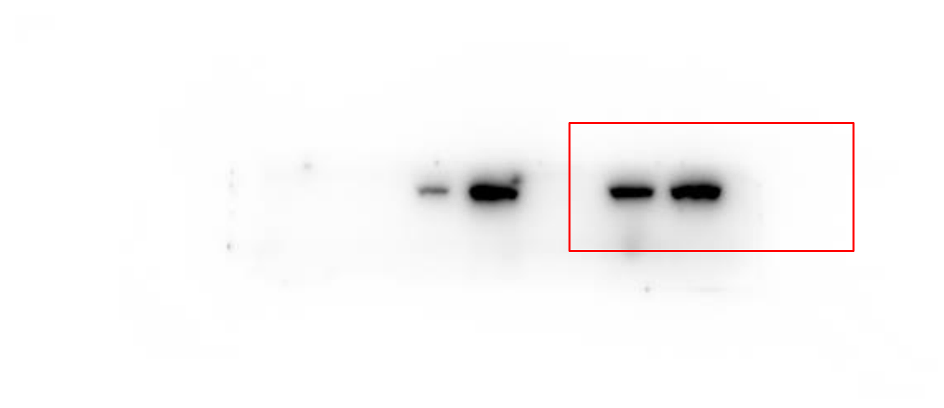

a-Tubulin

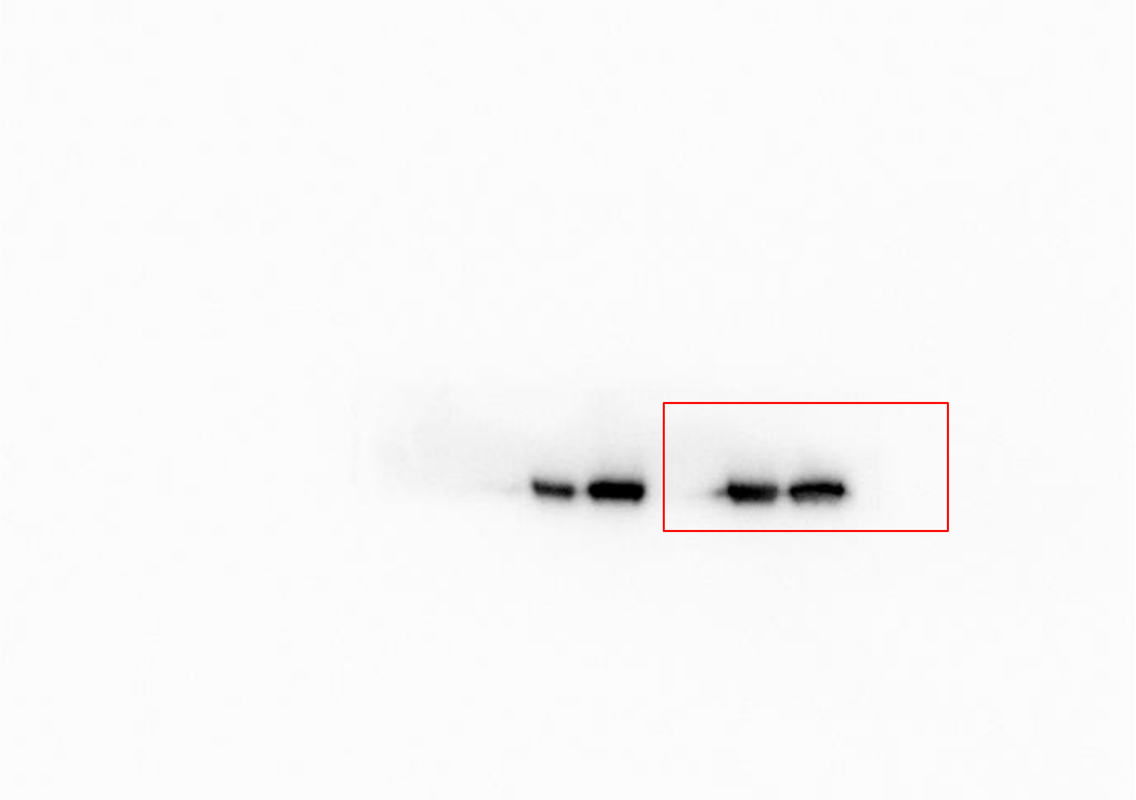

Full unedited gel for Figure 3A

DAZAP1

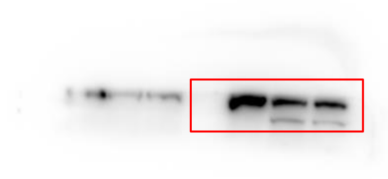

a-Tubulin

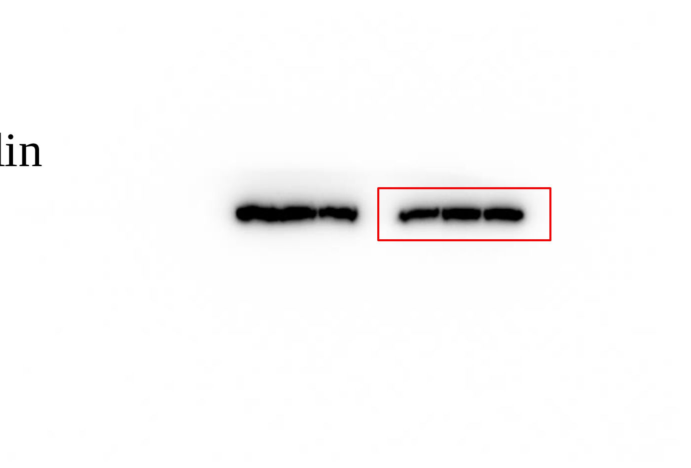

Full unedited gel for Figure 3A

DAZAP1

a-Tubulin

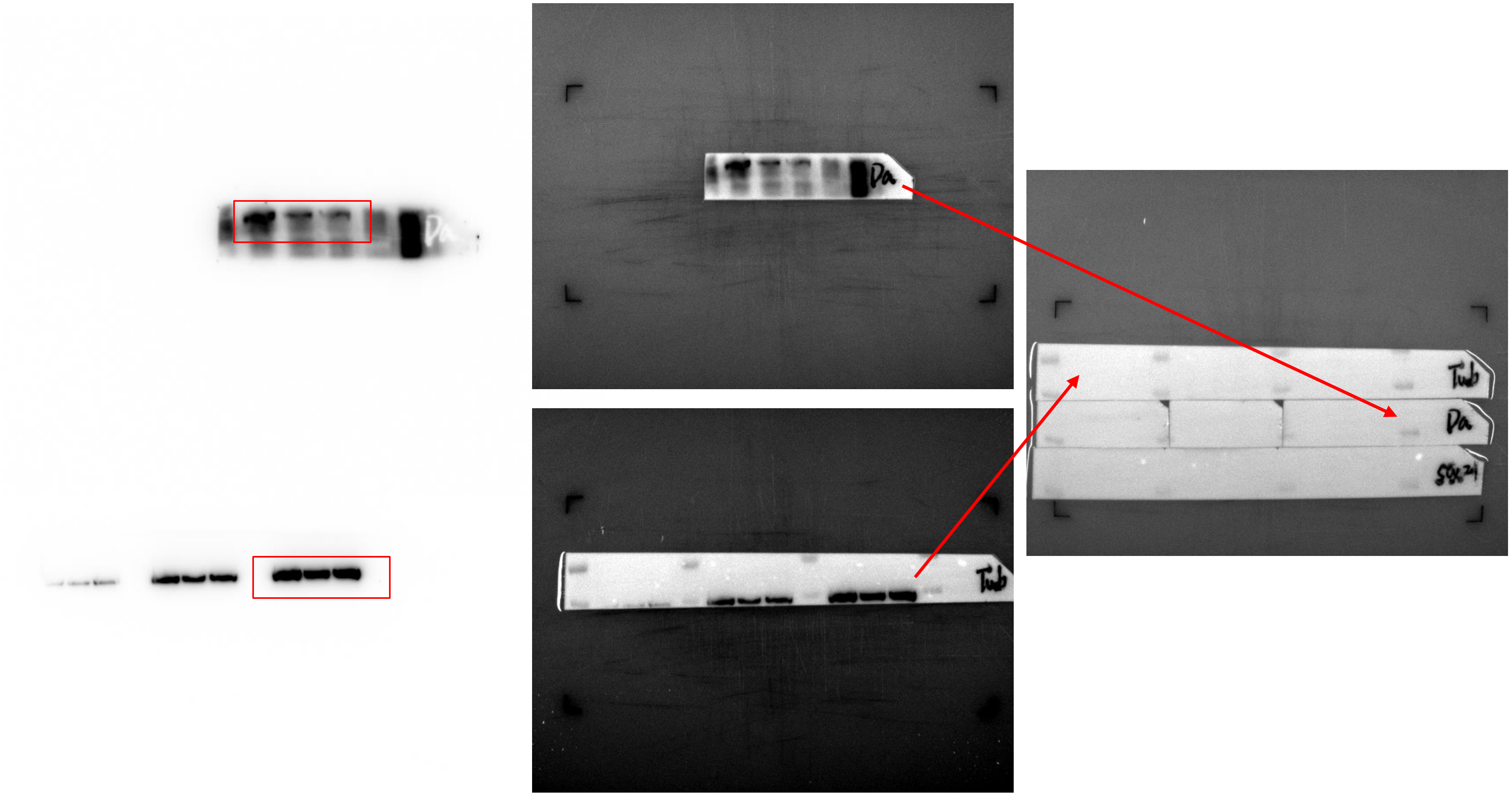

Full unedited gel for Figure 3A

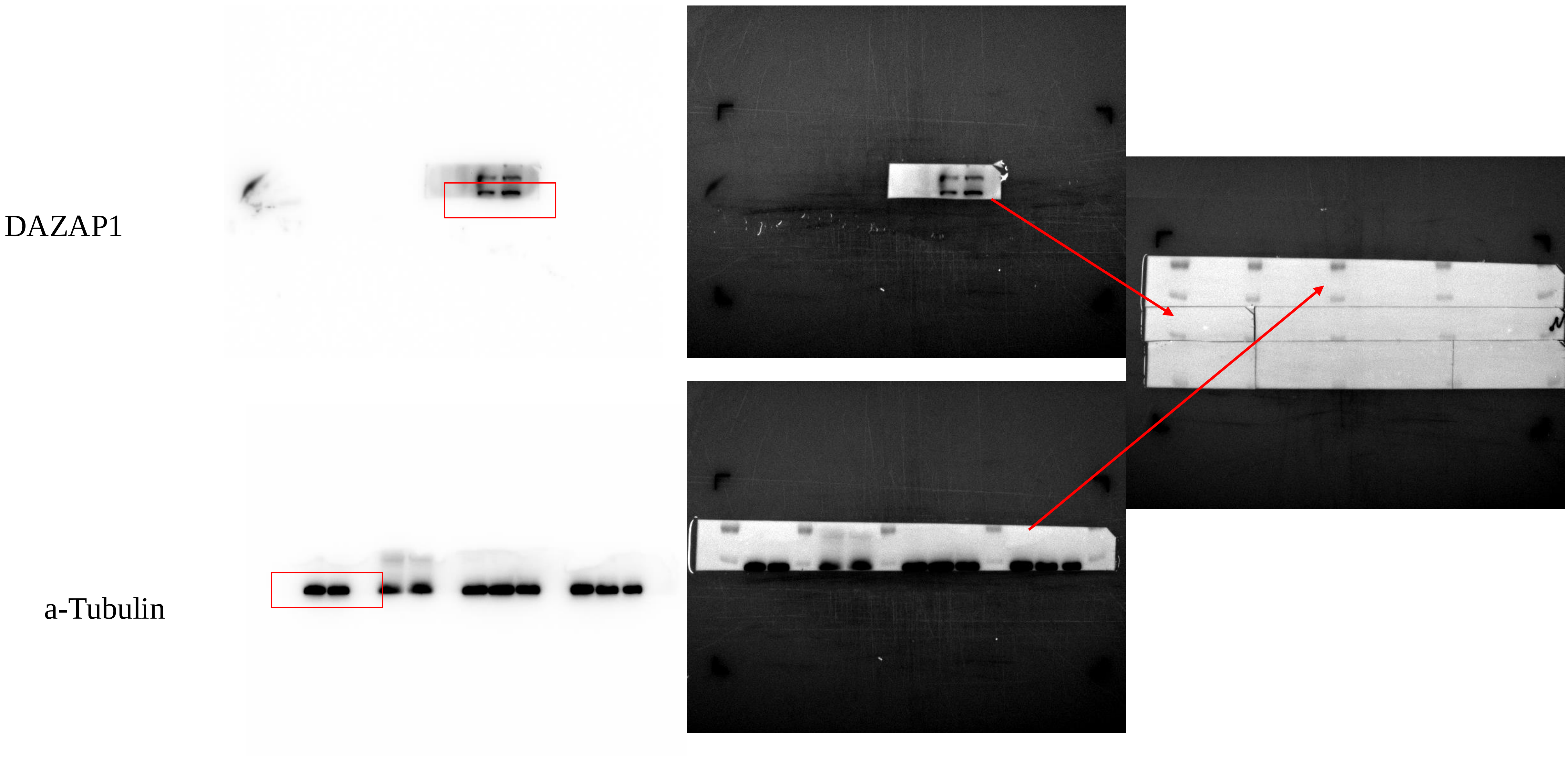

Full unedited gel for Figure 4G

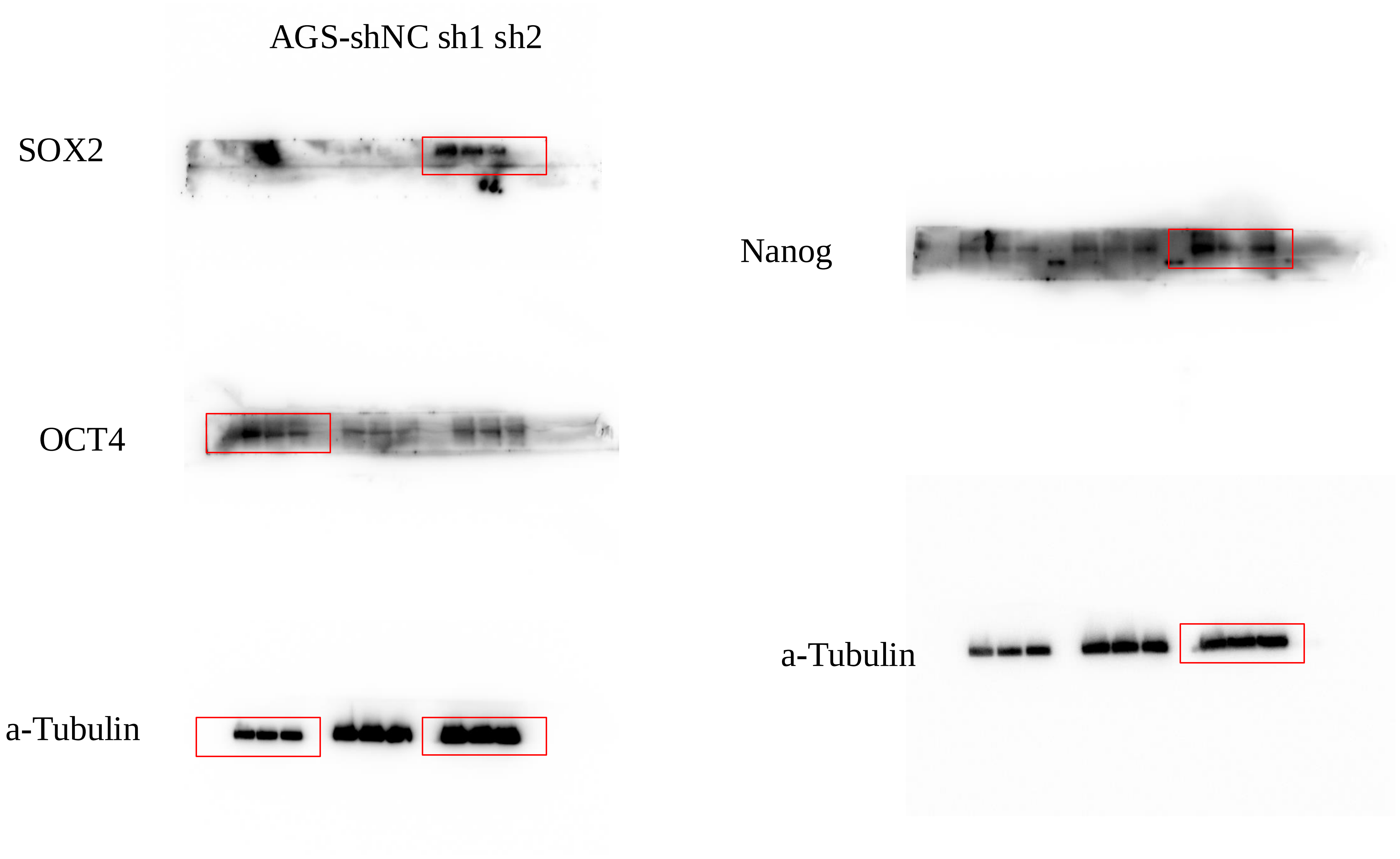

Full unedited gel for Figure 4G

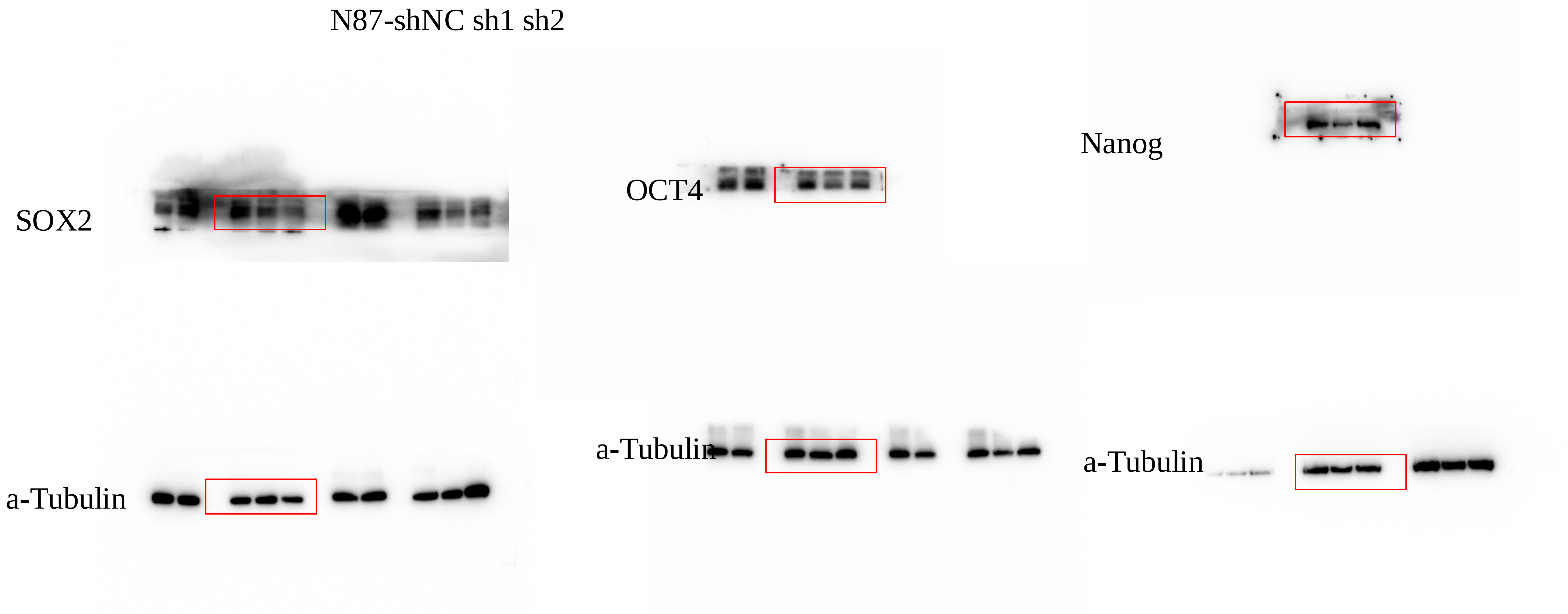

Full unedited gel for Figure 4G

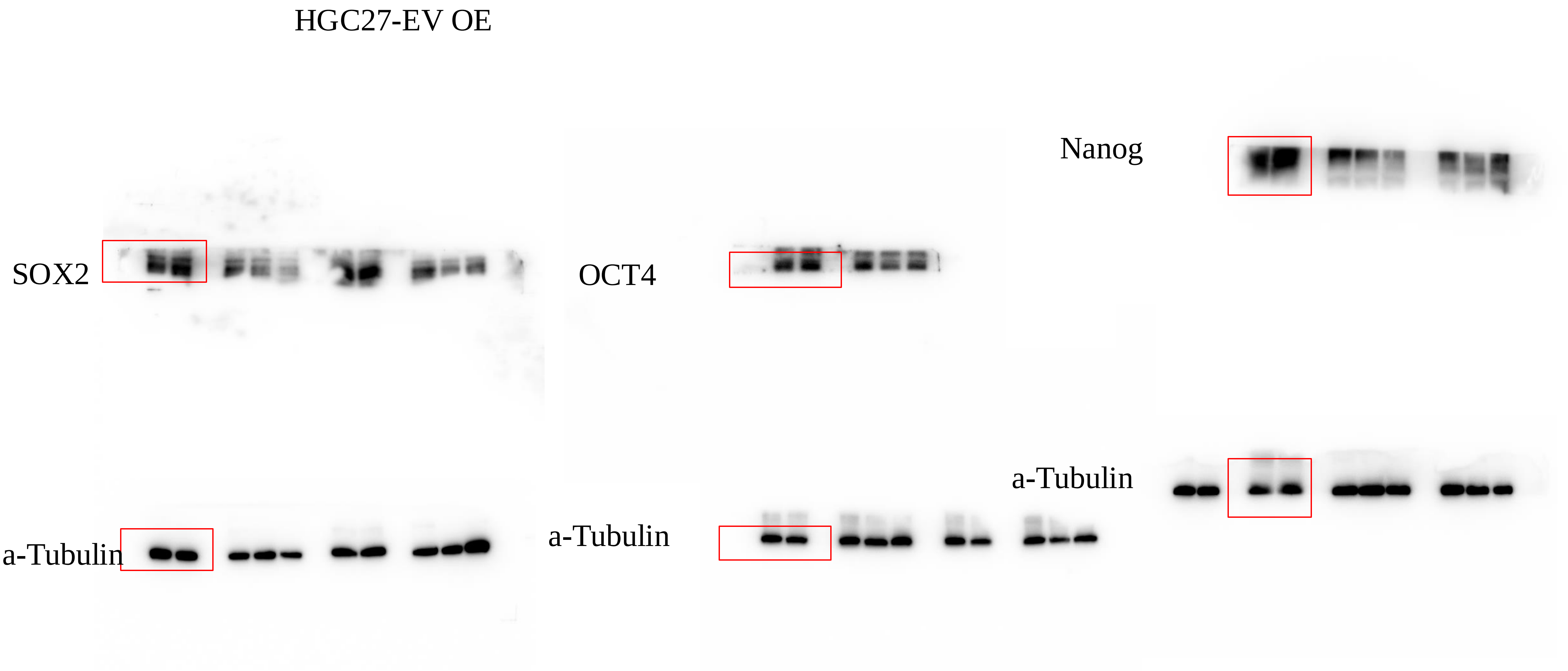

Full unedited gel for Figure 5N

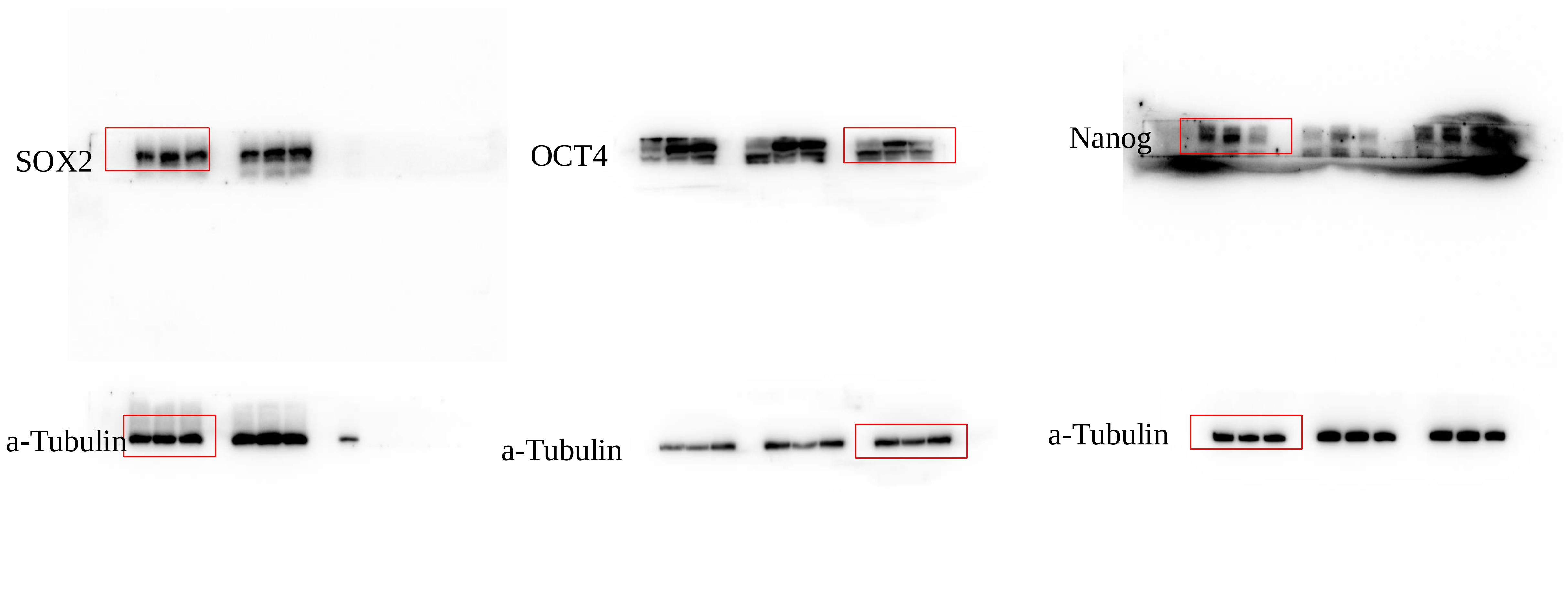

Full unedited gel for Figure 6K

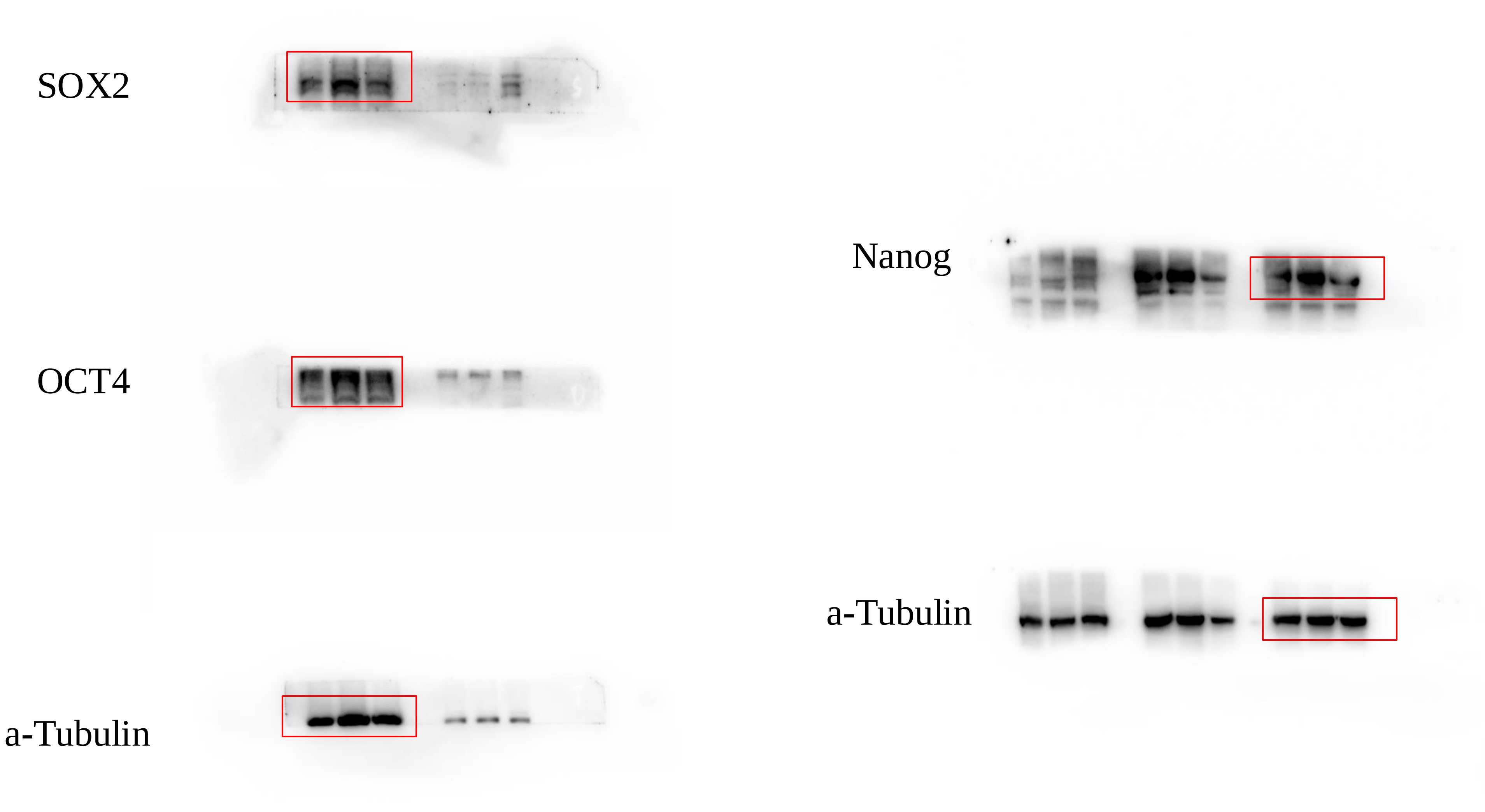

Full unedited gel for Figure 7B

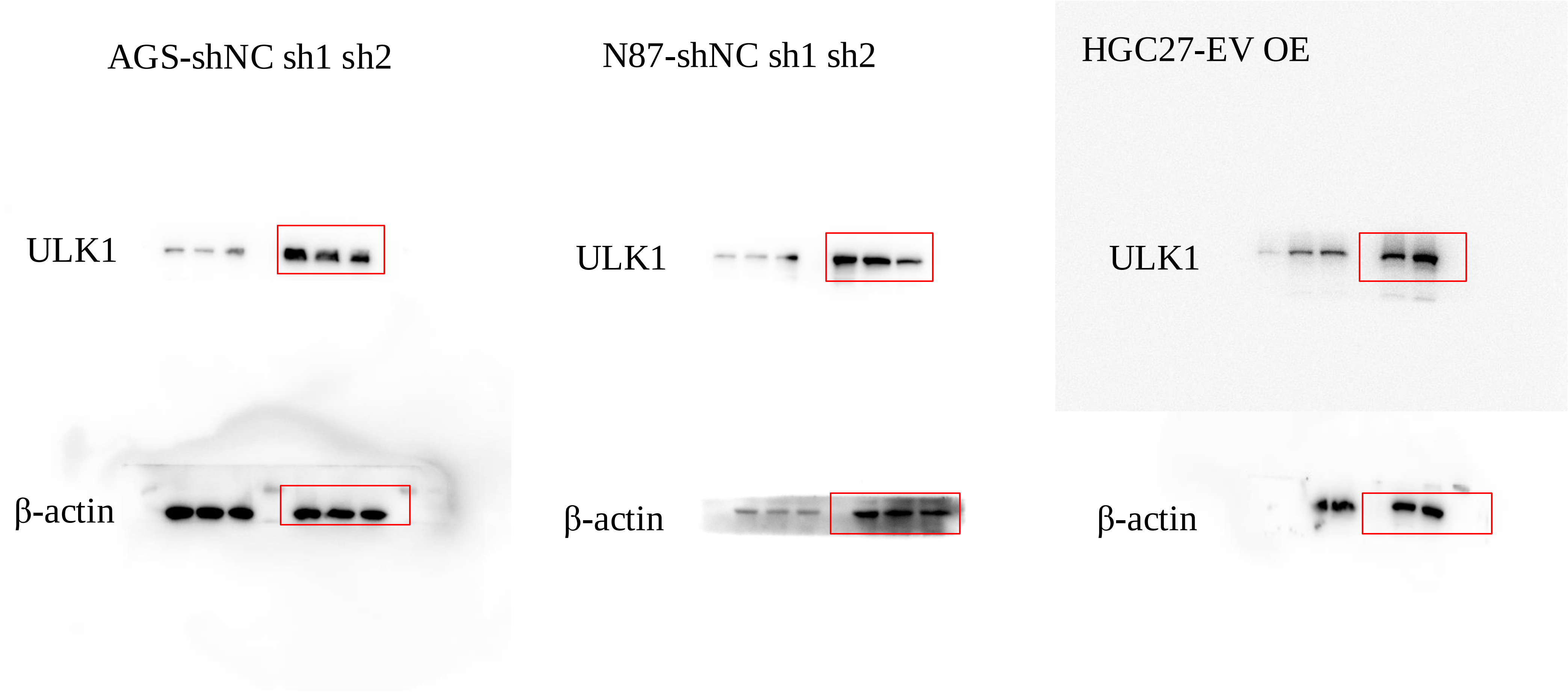

Full unedited gel for Figure 7K

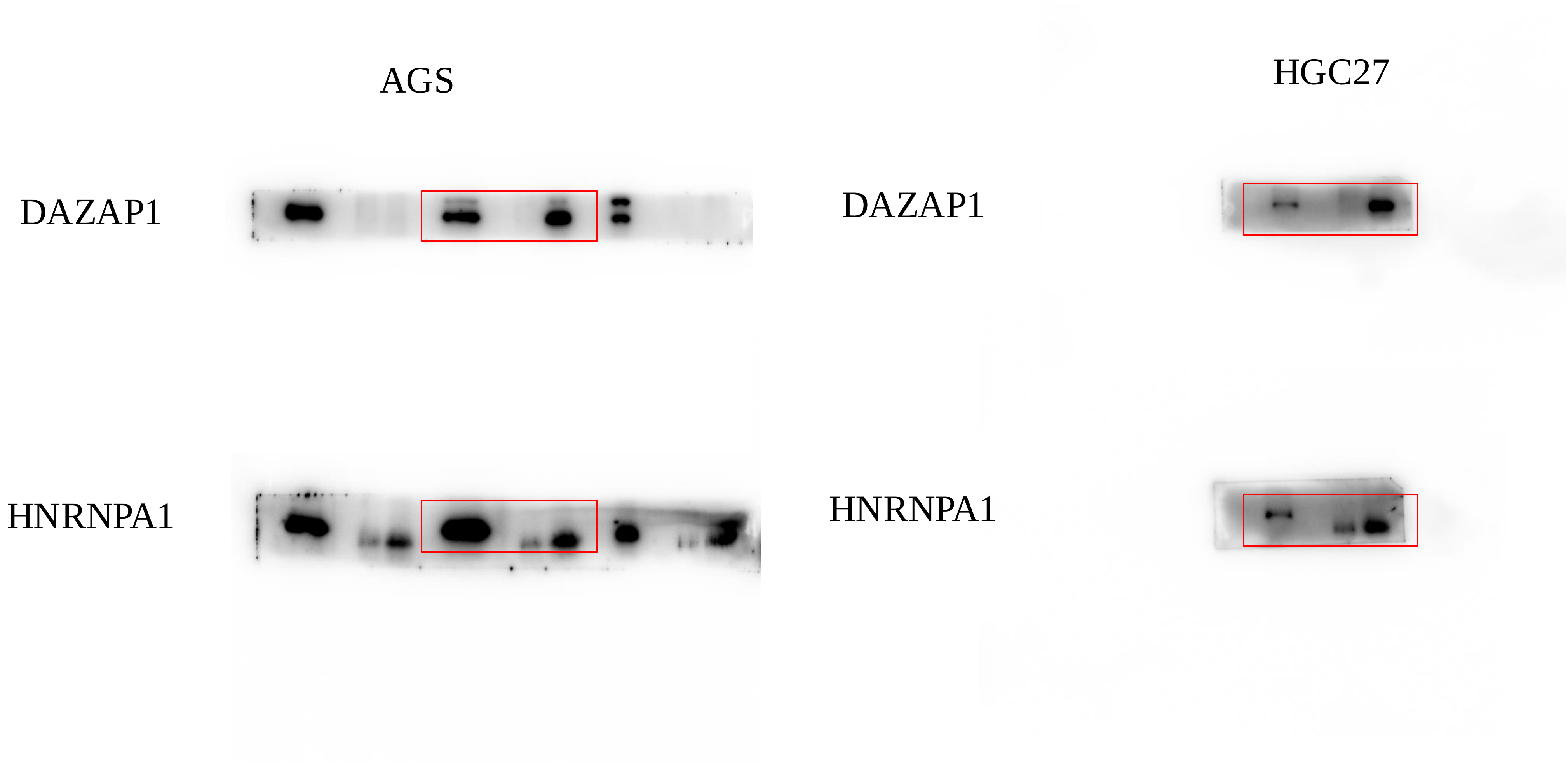

Full unedited gel for Figure 7N

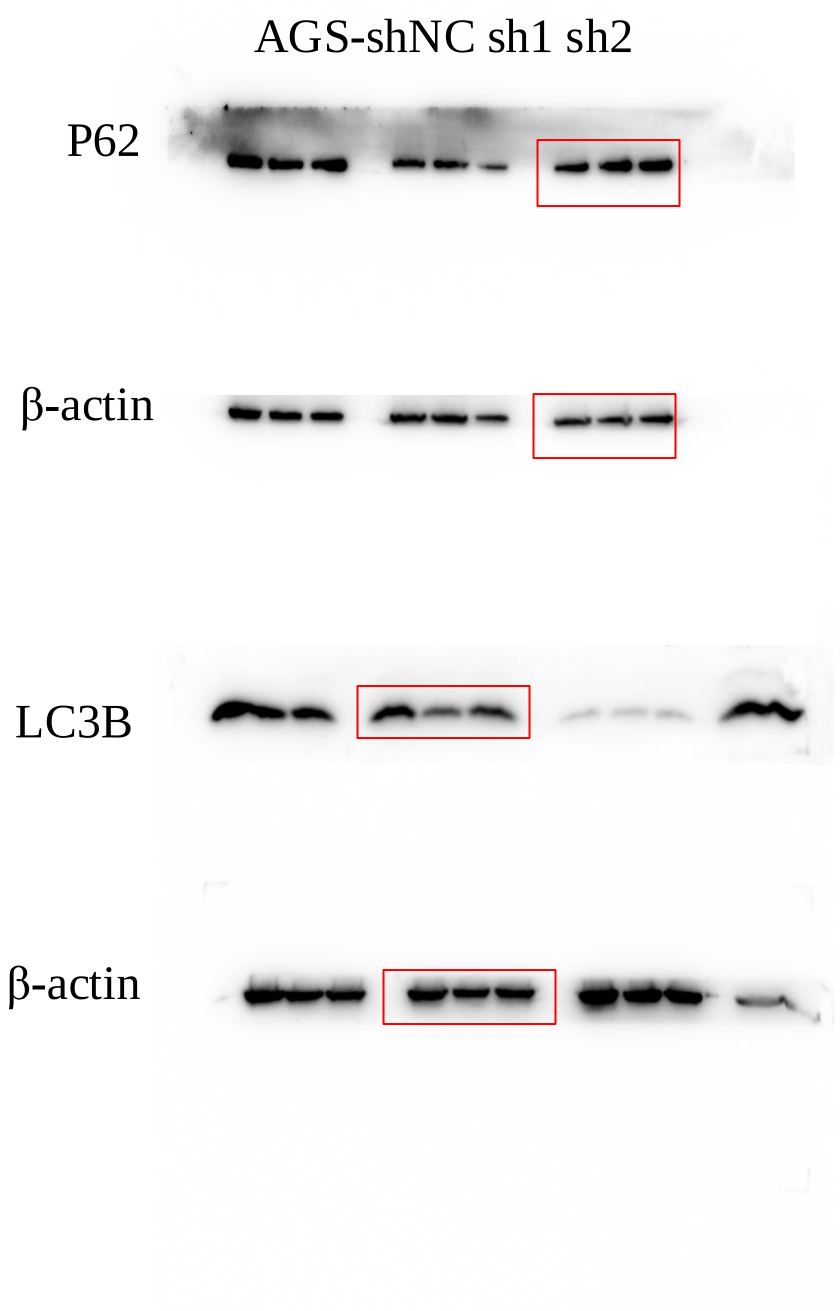

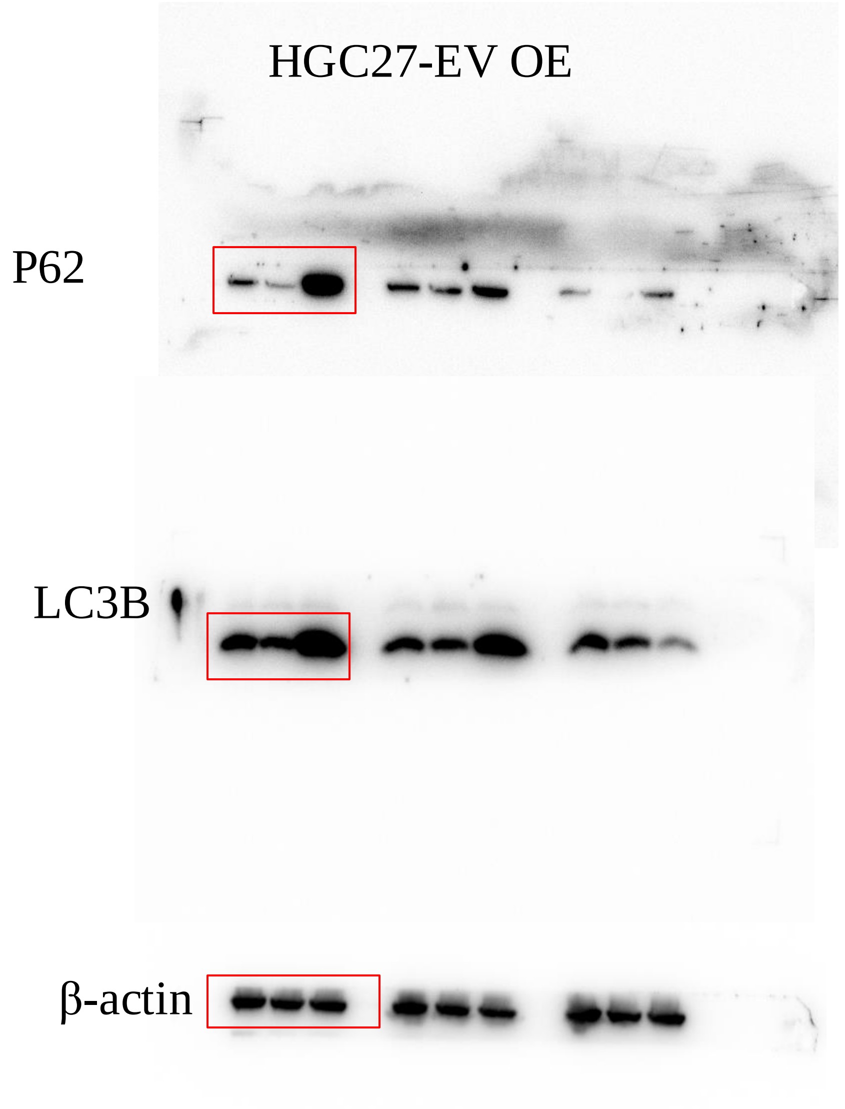

Full unedited gel for Figure 8A

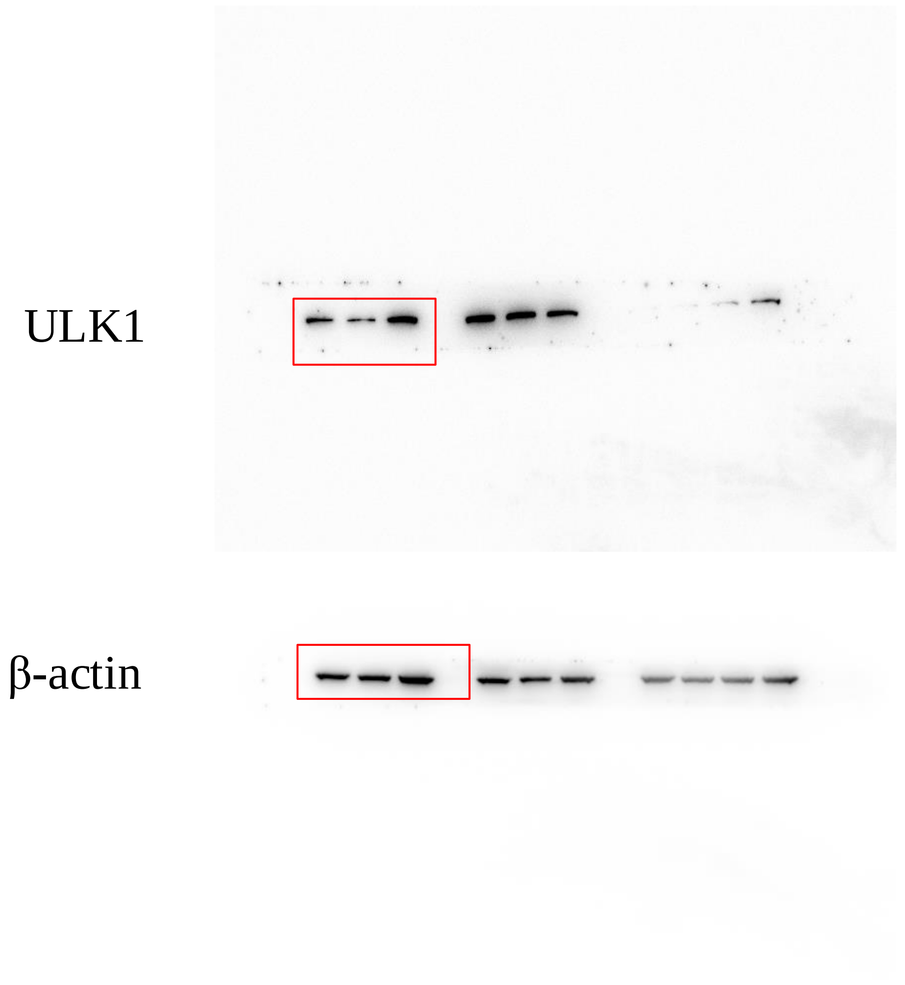

Full unedited gel for Figure 8B

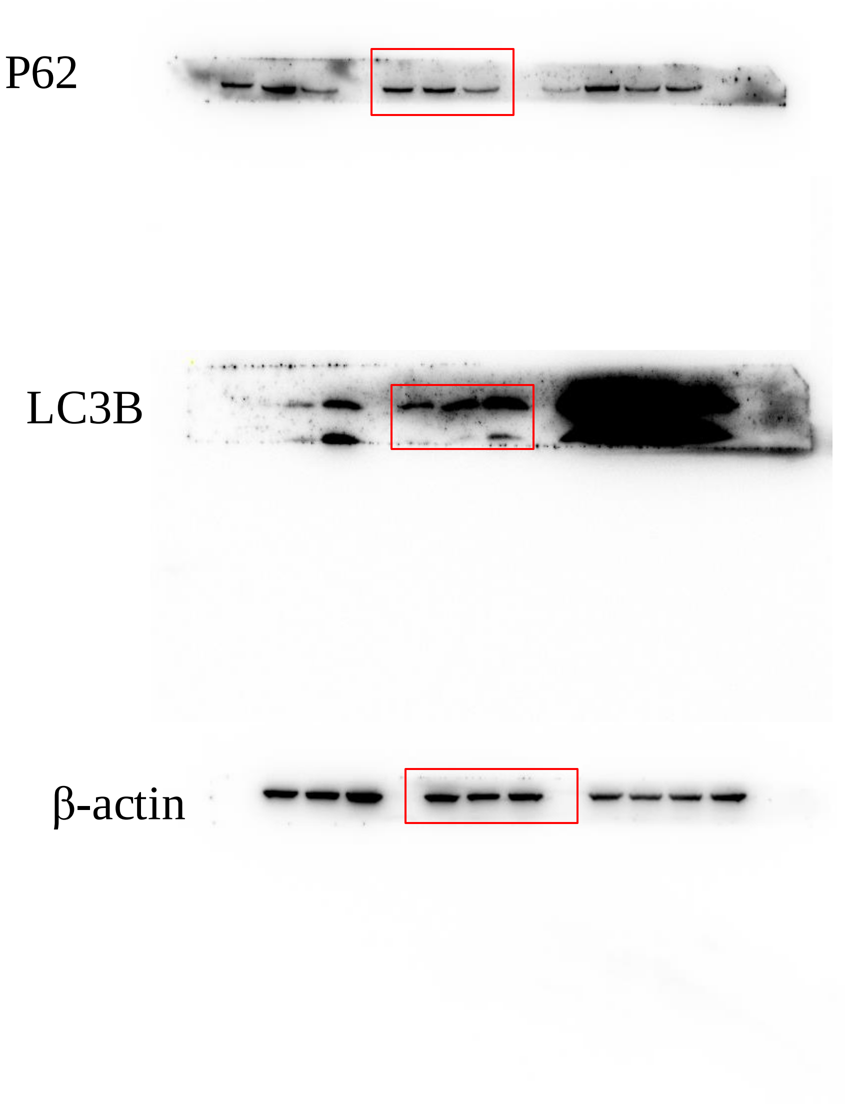

Full unedited gel for Figure 8K

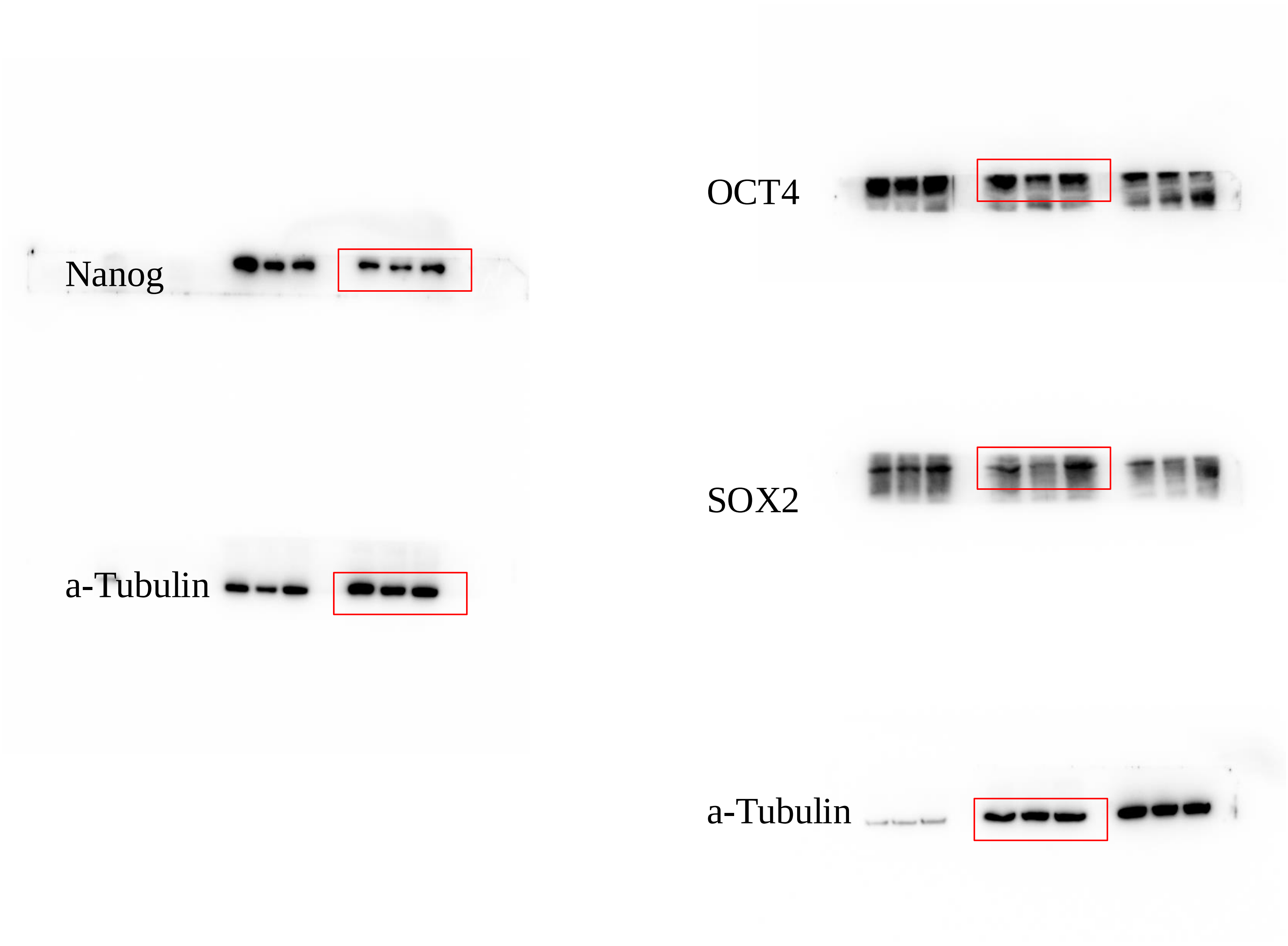

Supplement: Unedited blot and gel images [file jciinsight-10-175422-s082.pdf]
